# Comparative functional genomics analysis of bHLH gene family in rice, maize and wheat

**DOI:** 10.1186/s12870-018-1529-5

**Published:** 2018-11-29

**Authors:** Kaifa Wei, Huiqin Chen

**Affiliations:** 1 School of Biological Sciences and Biotechnology, Minnan Normal University, 36 Xian-Qian-Zhi Street, Zhangzhou, 363000 Fujian China; 2 School of Life Sciences, Tsinghua University, Beijing, 100084 China

**Keywords:** bHLHs, Gramineous crops, Expression regulation, Growth and development, Stress responses

## Abstract

**Background:**

The basic helix-loop-helix transcription factors play important roles in diverse cellular and molecular processes. Comparative functional genomics can provide powerful approaches to draw inferences about gene function and evolution among species. The comprehensive comparison of bHLH gene family in different gramineous plants has not yet been reported.

**Results:**

In this study, a total of 183, 231 and 571 *bHLH*s were identified in rice, maize and wheat genomes respectively, and 1154 *bHLH* genes from the three species and Arabidopsis were classified into 36 subfamilies. Of the identified genes, 110 *OsbHLH*s, 188 *ZmbHLH*s and 209 *TabHLH*s with relatively high mRNA abundances were detected in one or more tissues during development, and some of them exhibited tissue-specific expression such as *TabHLH454–459*, *ZmbHLH099–101* and *OsbHLH037* in root, *TabHLH559–562*, − *046*, − *047* and *ZmbHLH010*, − *072*, − *226* in leaf, *TabHLH216–221*, − *333*, − *335*, − *340* and *OsbHLH005*, − *141* in inflorescence, *TabHLH081*, *ZmbHLH139* and *OsbHLH144* in seed. Forty five, twenty nine and thirty one differentially expressed *bHLH*s were respectively detected in wheat, maize and rice under drought stresses using RNA-seq technology. Among them, the expressions of *TabHLH046*, − *047*, *ZmbHLH097*, − *098*, *OsbHLH006* and − *185* were strongly induced, whereas *TabHLH303*, − *562*, *ZmbHLH155*, − *154*, *OsbHLH152* and − *113* showed significant down-regulation. Twenty two *TabHLH*s were induced after stripe rust infection at 24 h and nine of them were suppressed at 72 hpi, whereas 28 and 6 *TabHLH*s exhibited obviously down- and up-regulation after powdery mildew attack respectively. Forty one *ZmbHLH*s were differentially expressed in response to *F. verticillioides* infection. Twenty two co-expression modules were identified by the WGCNA, some of which were associated with particular tissue types. And GO enrichment analysis for the modules showed that some *TabHLH*s were involved in the control of several biological processes, such as tapetal PCD, lipid metabolism, iron absorption, stress responses and signal regulation.

**Conclusion:**

The present study identifies the bHLH family in rice, maize and wheat genomes, and detailedly discusses the evolutionary relationships, expression and function of *bHLH*s. This study provides some novel and detail information about bHLHs, and may facilitate understanding the molecular basis of the plant growth, development and stress physiology.

**Electronic supplementary material:**

The online version of this article (10.1186/s12870-018-1529-5) contains supplementary material, which is available to authorized users.

## Background

The basic helix-loop-helix (bHLH) transcription factors constitute one of the largest transcription factor families in plants and are involved in a wide and diverse array of biological processes. A series of evidences showed that bHLHs participated in the regulation of plant growth and development including morphogenesis [1–3], iron homeostasis [4], root vascular cell proliferation [5], shoot branching [6], stomatal initiation [7], flowering time [8], pollen, gynoecium and fruit development [9, 10], and grain yield [11]. Previous studies revealed that bHLHs played very important roles in response of plants to abiotic stresses such as drought, salt and cold. *AtbHLH068* and *OsbHLH148* overexpressing in transgenic Arabidopsis and rice respectively conferred plant tolerance to drought stress via ABA- and JA-mediated signaling pathway [12, 13]. OsbHLH062, OsJAZ9 and OsNINJA formed a transcriptional regulation complex to fine tune the expression of JA-responsive genes involved in salt stress tolerance in rice, such as *OsHAK21* [14]. AtICE1, AtICE2, ZmmICE1, TaICE41 and TaICE87, the five MYC-like bHLHs, functioned as key regulators at the upstream of CBF (C-repeat binding factor) transcriptional cascade controlling cold tolerance [15–17]. TabHLH1 can mediate tobacco adaptation to osmotic stress via ABA-dependent pathway [18], and improve tolerance to Pi and N deprivation through transcriptional regulation of phosphate transporter, nitrate transporter and antioxidant enzyme encoding genes [19]. In rice, *OsPTF1* overexpression resulted in significantly higher total root length and surface area in response to Pi starvation [20]. And repression of *OsIRO2* led to lower mugineic acid family phytosiderophores (MAs) secretion and hypersensitivity to Fe deficiency [21]. Also, plant bHLHs involve in pathogen stress adaptation and resistance development. *OsDPF* was induced in rice leaves by blast infection, and *DPF* overexpressing and *DPF* knockdown rice led to remarkably increased and decreased accumulation of momilactones and phytocassanes, respectively [22]. And wheat bHLH060 overexpression negatively regulated plant resistance to *Pseudomonas syringae* through jasmonic acid (JA) and ethylene (ET) signaling in transgenic Arabidopsis [23]. Although some functions of bHLHs have been characterized, the biological functions of most plant bHLHs remain unclear, especially in gramineous crops such as rice, maize and wheat.

The bHLHs are characterized by the signature domain which consists of two functionally distinctive regions, the basic and helix-loop-helix (HLH) regions. The basic region located at the N-terminus contains approximately 17 residues, which is typically rich in basic amino acids, and the region with at least five basic amino acids is expected to recognize and bind specific DNA sequence. In the region, Glu-13 and Arg-16 are essential in E-box-binding recognition, and two additional residues His/Lys-9 and Arg-17 provide DNA-binding specificity for G-box, a specific type of E-box [24]. According to the sequence information in the region, plant bHLHs can be divided into two categories: DNA- and non DNA-binders. The HLH region includes two amphipathic α-helices separated by a loop of variable length and sequence, allowing the formation of homodimers or heterodimers. Additionally, the bHLH domain is composed of around 60 amino acids, of which 25 are conserved residues, including five in the basic region, six in the first helix, two in the loop, and 12 in the second helix [25]. Based on phylogenetic analysis, 638 *bHLH*s, including 167 from Arabidopsis, 177 from rice and the rest from poplar, moss and algae, were classified into 32 subfamilies [25]. The more species genomes had been sequenced, the more studies about bHLH gene family were reported, such as 230 genes organized into 24 subfamilies in the Chinese cabbage [26], 152, 159 genes separated into 21 or 26 subfamilies in tomato [27, 28], 117 genes assigned to 23 subfamilies in *Nelumbo nucifera* [29], 127 genes grouped into 25 subfamilies in *Salvia miltiorrhiza* [30], 155 genes clustered into 21 subfamilies in common bean [31], and 197 genes divided into 24 subfamilies in maize [32]. Also, conservative motifs outside the domain region were identified, and most of them were conserved within a subfamily.

Rice, maize and wheat are the three leading food crops in the world, and the grain yield is severely affected by adverse environmental conditions. Uncovering the molecular mechanism underlying the roles of bHLHs in plant growth, development and stress responses may contribute to genetics and molecular breeding. It’s indispensable to perform whole-genome identification and expression analysis for wheat bHLH gene family referencing new hexaploid bread wheat (*Triticum aestivum*) genome. The rice pseudomolecules were reconstructed and the new annotations were released in 2011, and therefore, it is necessary to perform a genome-scale analysis for rice *bHLH*s based on new genome assembly and expression data. Similarly, the identification of atypical *bHLH*s and expression analysis of *bHLH*s were not reported in maize. In this study, we aim to build connections between genetic variation and phenotypic evolution for Arabidopsis, rice, maize and wheat. For this purpose, full-genome identification and comparative evolutionary analysis of bHLH family in *Arabidopsis thaliana* (TAIRv10), *Oryza sativa* (MSUv7.0), *Zea mays* (AGPv3) and *Triticum aestivum* genome (TGACv1, updated in September 2016) were performed. The expression patterns and functions of *TabHLH*s, *ZmbHLH*s and *OsbHLH*s during plant life cycle and under biotic and abiotic stresses were systematically investigated. Then, WGCNA (weighted gene co-expression network analysis) and the Gene Ontology (GO) enrichment analysis were conducted to identify wheat tissue-specific and stress-responsive genes.

## Results

### Identification and prediction of DNA-binding ability for bHLH proteins

A total of 571, 183 and 231 *bHLH* genes were identified in wheat, rice and maize, respectively (Additional file 1: Table S1). Of the 571 *TabHLH*s, 180 genes may be the same as those previously reported by Xiao-Jiang Guo et al. [33], as listed in Additional file 2: Table S2. Other 45 out of 225 *bHLHs* identified in the previous study did not match any of the 571 *TabHLHs* in the current study. Obviously, there are some great differences between the old and new genome assembly versions. It may be resulted from complicated genome assembly of wheat with large genomes, polyploidy and a high proportion of repetitive elements. For maize and rice, 36 and 10 *bHLH*s were novel. All *TabHLH*s and *ZmbHLH*s and 10 novel *OsbHLH*s (*OsbHLH179–188*) were renamed. For multi-transcript genes, a putative transcript with fewer mismatches from bHLH consensus motif and longest sequence length was chosen to represent each of them. As shown in Additional file 1: Table S1, *bHLH*s account for approximately 0.55, 0.47, 0.59, and 0.61% of the wheat (103,539), rice (39,045), maize (39,469) and Arabidopsis protein-coding genes (27,655), respectively, constituting one of the largest transcription factor families in the species. The TabHLH domains with the highest number (361, 63.22%) are composed of 60 amino acids, and those with the second (87, 15.23%) and third highest numbers (52, 9.11%) consist of 61, 62 residues, respectively. The loop regions within most of TabHLH domains (364, 63.75%) are six residues long but divergent in terms of amino acid composition. Based on information of gene annotation, 176, 193 and 183 *TabHLH*s are non-randomly distributed in the A, B and D sub-genomes respectively, while 19 *TabHLH*s are located on scaffolds. The minimum of 12, 13 and 14 *TabHLH*s are localized on chromosome 1A, 1B and 1D, while the maximum of 42, 42 and 35 on chromosome 5A, 4B and 4D, respectively (Additional file 3: Table S3).

**Table 1:** Predicted DNA-binding categories based on the bHLH domain

	> = 5 basic amino acids			< 5 basic amino acids			Total
	G binder	E non G	non E binder	E-box	G-box	non DNA binder	
Arabidopsis	88	20	38	4	1	18	169
Rice	89	18	30	4	2	40	183
Wheat	291	85	84	8	3	100	571
Maize	117	25	38	5	2	44	231
Total	585	148	190	21	8	202	1154

Using the criteria proposed by Xiaoxing Li et al. [34], all the 571 TabHLHs and 231 ZmbHLHs were respectively divided into two major groups based on sequence information of the basic region within bHLH domains Table 1 (i) a large group of 460 TabHLHs or 180 ZmbHLHs containing five to eleven basic residues within their basic region were expected to bind DNA, and (ii) a smaller group of 111 TabHLHs or 51 ZmbHLHs with low basic region were tentatively predicted to be non DNA binders. The DNA-binding bHLHs were further classified into two subgroups: E-box binders (376 TabHLHs or 142 ZmbHLHs) and non E-box binders (84 TabHLHs or 38 ZmbHLHs), depending on whether both Glu-13 and Arg-16 are present in the basic region. Of E-box binder subgroup, 291 TabHLHs and 117 ZmbHLHs were predicted to bind G-boxes, which contain additional residues His/Lys-9 and Arg-17 at the basic region. Additionally, according to the criteria suggested by Carretero-Paulet et al. [25], a subset of non DNA binders containing the essential residues in E-box- (eight TabHLHs and five ZmbHLHs) and G-box-binding (three TabHLHs and two ZmbHLHs) recognition motifs were potential DNA-binding bHLHs. Our prediction results of the DNA-binding ability of four AtbHLHs (AtbHLH026, AtbHLH047, AtbHLH109 and AtbHLH142) and 20 OsbHLHs (OsbHLH006, OsbHLH007, OsbHLH012, OsbHLH042 and so on) were different from that reported by Carretero-Paulet et al., as can be seen in Additional file 1: Table S1.

### Multiple sequence alignments and phylogenetic tree construction of *bHLH*s

**Fig. 1:**
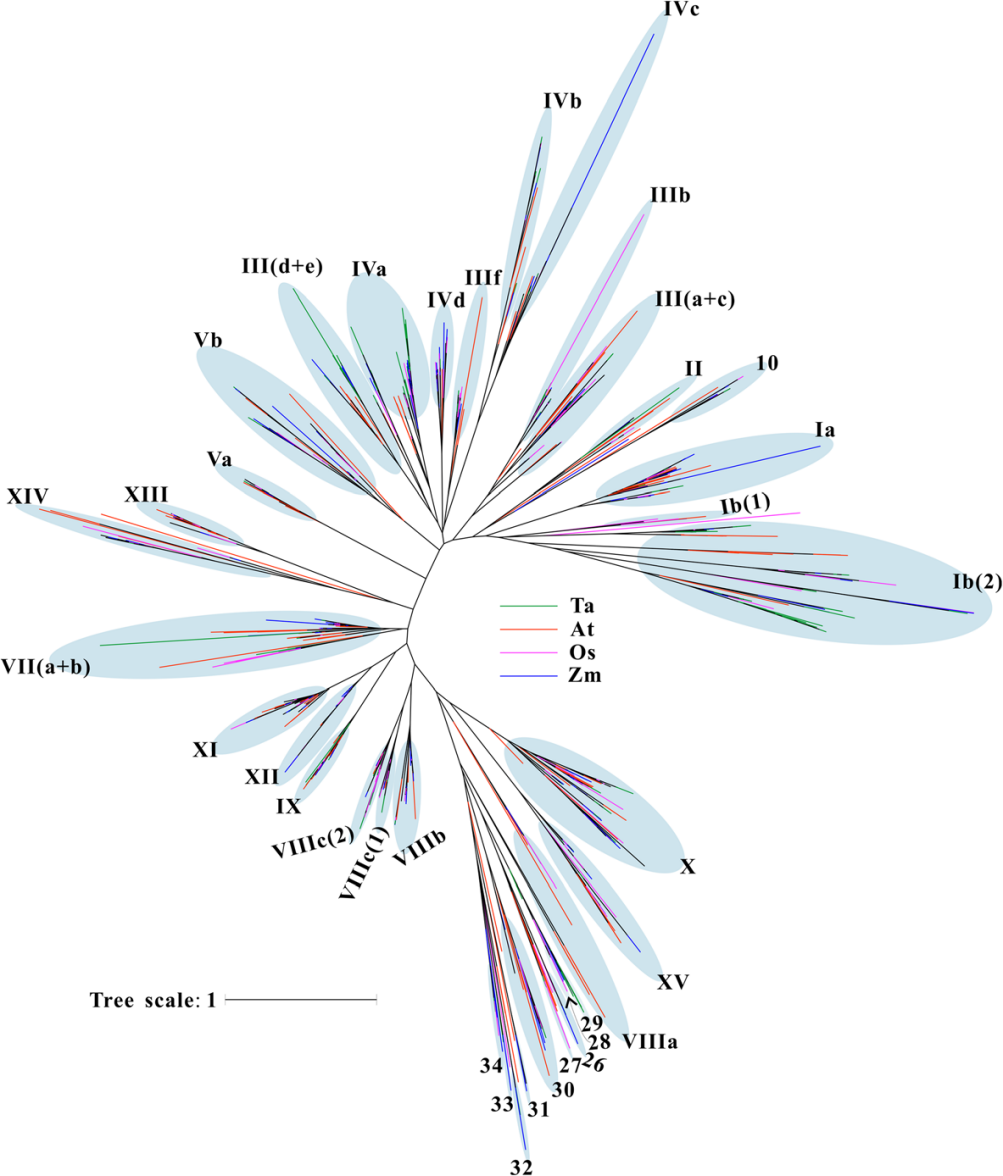
Phylogenetic relationship among Arabidopsis, rice, maize, and wheat bHLH proteins. An unrooted cladogram shows the phylogenetic relationships among 1154 bHLHs from *Arabidopsis thaliana* (At), *Oryza sativa* (Os), *Zea mays* (Zm) and *Triticum aestivum* (Ta). The blue balloons delineate the 36 subfamilies of bHLH proteins. Colored lines symbolize the species to which the proteins belong (red: *Arabidopsis thaliana*; purple: *Oryza sativa*; blue: *Zea mays*; green: *Triticum aestivum*). A full tree with protein names, proportional branch lengths, and clade support values is given in Additional file 5: Figure S2

As the flanking sequences of the bHLH proteins from independent subfamilies are generally too divergent to be reliably aligned, the bHLH domain was used for this analysis. From the alignment, we identified 31 residues that are conserved in at least 50% of the 1154 bHLH domains from Arabidopsis, rice, maize and wheat (Additional file 4: Figure S1, indicated at the bottom of the alignment). An unrooted NJ phylogenetic tree was constructed using the alignment of the bHLH domain sequences with bootstrap analysis (1000 replicated) to observe the evolutionary relationship of bHLHs in four species (Fig. 1 and Additional file 5: Figure S2). A total of 1136 *bHLH*s were grouped into 36 subfamilies according to the clades with at least 50% support, topology of the tree and the classification of the Arabidopsis and rice [34–36]. The internal nodes have low support and therefore the evolutionary relationships between different bHLH subfamilies could not be inferred. The remaining 18 *bHLH*s were considered as orphans, likely representing highly diverged lineage-specific genes. Among the 36 subfamilies, 26 subfamilies are consistent with previously defined groups [36]. And 10 new subfamilies (10 and 26–34) are formed by 73 *bHLH*s which are from monocotyledons except for *AtbHLH151* (previously considered member of subfamily IVd by Nuno Pires and Liam Dolan [36]), *AtbHLH147*, *AtbHLH148*, *AtbHLH149* and *AtbHLH150* (previously classified as orphans). As shown in Additional file 6: Table S4, 28 subfamilies are common to the four species, while the remaining 8 subfamilies are from wheat and maize and/or rice, indicating that these *bHLH*s might have formed after the divergence of the monocotyledon and dicotyledon, and be required for monocotyledon-specific traits. Interestingly, we noted 29 *TabHLH*s along with 42 *PIFs* and *PIF-likes* (*PILs*) identified in Arabidopsis (15), rice (13) and maize (14) clustered in subfamily VII(a + b) [32, 37, 38]. Twenty four, fifteen, and twelve TabHLHs were respectively clustered into three subfamilies III(d + e), IIIf and XIII, which MYB-interacting-region (MIR) containing proteins AtTT8 (AtbHLH042), AtEGL3 and AtLHW belong to. TabHLH183 and − 184 share high sequence similarities with AtMYC2 at amino acid level. While only 19 ZmbHLHs are found in the three subfamilies (ten in subfamily III(d + e), two in subfamily IIIf and seven in subfamily XIII), of which ZmbHLH103 and − 104 are homologous to OsMYC2 (OsbHLH009).

### Intron distribution pattern within the bHLH domain

To better understand the gene structural features of *TabHLH*s and *ZmbHLH*s, their intron/exon organization and splicing phase were analyzed based on their phylogenetic relationships (Additional files 7 and 8: Figures S3 and S4). In full-length genes, the exon number of *TabHLH*s varies from 1 to 12; and 64 genes are intronless. Contrastingly, *ZmbHLH*s contain up to 14 exons; and 45 genes are intronless. It was observed that the structures of genes within a subfamily show high similarity. To name a few, all members of subfamilies XIV, 26, 27, 32 and 34 are intronless, and most genes in subfamilies VIIIb, Vb, IVc contain one, two and five exons, respectively. Additionally, a great number of exons are symmetric with phase zero which is likely to facilitate gene assembly via exon shuffling and recombination [39].

**Fig. 2:**
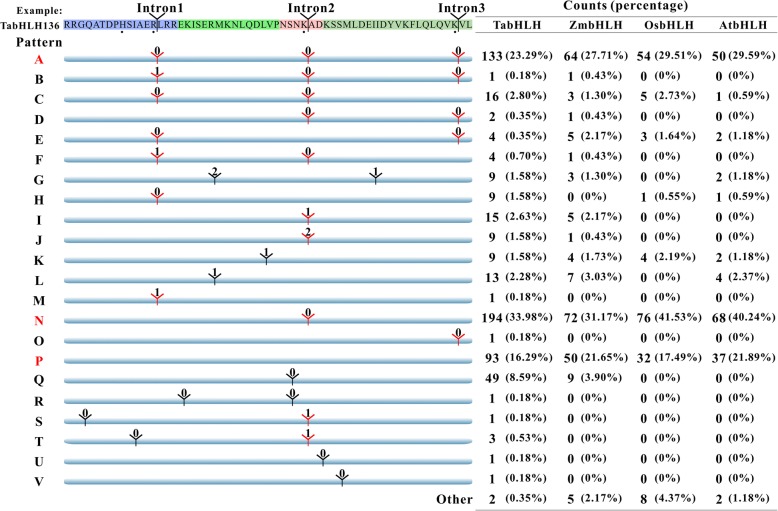
Intron distribution within the bHLH domain of the AtbHLH, OsbHLH, ZmbHLH, TabHLH proteins. Scheme of intron distribution patterns (designated A to V) within the bHLH domains. Position of introns is indicated by “丫” based on the bHLH region of TabHLH136, which is shown at the top, and the number corresponds to the intron phase. The count and percentage of bHLHs displaying each pattern in wheat (Ta), Arabidopsis (At), rice (Os) and maize (Zm) are given in the table at right

Furthermore, we analyzed the intron distribution, relative position and phase within the coding sequence of bHLH domains for each gene, and found 22 different intron patterns designed as A to V (Additional file 1: Table S1). Six patterns B, D, F, I, J and Q were not found in rice and Arabidopsis, and M, O, R-V were found only in wheat. The intron number varies from zero to three and their lengths are quite different even at the same position (Additional files 7 and 8: Figures S3 and S4). As can be seen in Fig. 2, 93 *TabHLH*s, 50 *ZmbHLH*s, 32 *OsbHLH*s and 37 *AtbHLH*s do not contain intron within their bHLH domain encoding regions, forming the third common pattern P. Pattern A containing three phase-zero introns at three highly conserved positions (indicated by red “丫”) is the second common pattern in the four species. The most common pattern N has only one phase-zero intron at loop region in the species, which widely distributed across 18 subfamilies (Additional file 6: Table S4). Intron pattern distribution within most subfamilies was almost absolutely conserved, which provide a reliable support to our phylogenetic analysis. For example, pattern A was observed in 93.55% (87 out of 93) and 91.76% (78 out of 85) of XII and X subfamily members, respectively. Pattern P is presented in each member of subfamilies XIV, VIIIb, 26–28, 30, and 32–34.

### Conserved motifs in most bHLH subfamilies

We searched for amino-acid sequence patterns in our data set of bHLH proteins according to their evolutionary relationships, and then each motif was characterized and named as motif 1 to 38 (Additional file 9: Table S5). The relative position of most motifs is conserved, as shown in the Additional file 10: Figure S5. bHLH domain includes motif 1 (21 amino acids in length) covering partial DNA-binding and complete helix 1 regions and motif 2 (21 amino acids) identified as a part of the loop and helix 2 regions. Outside the domain, subfamily-specific motifs were found, and some of them have been characterized as defining additional functional properties. Motifs 6, 5, and 10 observed in the majority members of subfamilies Ia, IVa and II have been reported to form a high conserved C-terminal domain (the SMF domain) of AtSPCH, AtMUTE and AtFAMA [7]. Additionally, motifs 6 and 5 were also detected in a great many proteins in subfamilies IIIf and III(d + e), such as TabHLH239, AtMYC2, TabHLH184 and ZmbHLH103, and we found they were significantly matched with an ACT domain that contributed to the recruitment of the C1 R2R3-MYB factor to the C1 binding sites located in the promoters of flavonoid biosynthetic genes [40]. Motif 7, observed in all members of subfamilies IVb and IVc, was unequivocally characterized as a ZIP dimerization domain. Motifs 13, 27, 11, 32 and 15, conserved among most members of subfamilies IIIf and III(d + e), overlap with the MIR and MYC_N domain which can interact with JAZs [41]. Interestingly, motifs 13, 27, 33, 32 and 15 form the N-terminal region of subfamily XIII proteins, which was likely responsible for activating transcription [42]. Then we discussed a detailed analysis of the structure of N-terminal fragment of proteins in the three subfamilies (IIIf, III(d + e) and XIII) through mapping secondary structure elements of N-termini of MYC3 (PDB code: 4RRU) onto the sequence alignment, as illustrated in Additional file 11: Figure S6. The five motifs (13, 27, 11, 32 and 15) respectively correspond to distinct regions: α2-helix to β1-sheet, β2, α4 to α5, α7 to β5 and β6 to α8, and the last one overlaps the transcription activation domain (TAD). Motif 12 shared by members (except OsbHLH188) of subfamily XIII has been characterized in AtLHW as necessary for homodimerization [42]. Motif 38 is present in several proteins of subfamily VII(a + b) and overlaps with the N-terminal of active phytochrome binding (APB) motif [43]. Besides, some other motifs demonstrate subfamily-specificity, yet their functions are still unclear. For instance, motif 4 is observed in almost all members of subfamily XI (except ZmbHLH048) and X (except TabHLH360 and OsbHLH179). bHLHs in subfamilies XV and XII have a motif 8 immediately C-terminal to the second helix. Motif 9 is adjacent to the N-terminal of motif 1 in subfamily Ia.

### *Cis*-regulatory elements analysis

The *cis*- regulatory elements (CREs) are essential for gene expression, which participate in the control of plant growth, development and stress responses. In order to investigate the possible biological functions and regulation network of *TabHLH*s involved in, CREs in the 1500 bp nucleotide sequences upstream of the 5′-UTR of these genes were identified. A total of 128 distinct CREs were found in that region of *TabHLH*s (Additional file 12: Table S6). Among them, light response elements, such as G-box, ACE, AE-box, ATCT-motif and Box I, were abundantly presented in the promoter region of *TabHLH*s. A great number of CREs required for the regulation of particular tissue development were detected, such as AACA, GCN4 and skn-1 motifs and prolamin-box for endosperm, RY-element for seed, as1 for root, and HD-Zip 1 and 2 for leaf. Fifteen types of stress responsive element were found, e.g. box E, C-repeat/DRE, W-box, JERE and LTR. At least one hormone-responsive element, particularly ABRE (ABA-responsive), TCA-element (SA-responsive), CGTCA- and TGACG-motifs (JA-responsive), is presented in the promoter region of most genes. Seventy one genes contain at least one cell cycle-related regulatory motif, including E2Fa, E2Fb and MSA-like. Twenty eight genes have MBSI and/or MBSII, which are the MYB binding sites involved in flavonoid biosynthetic genes regulation. The different types of CREs presenting in *TabHLH*s indicate functional diversity and complexity of the regulatory networks.

### Expression profiles of *bHLH*s in different tissues and developmental stages

**Fig. 3:**
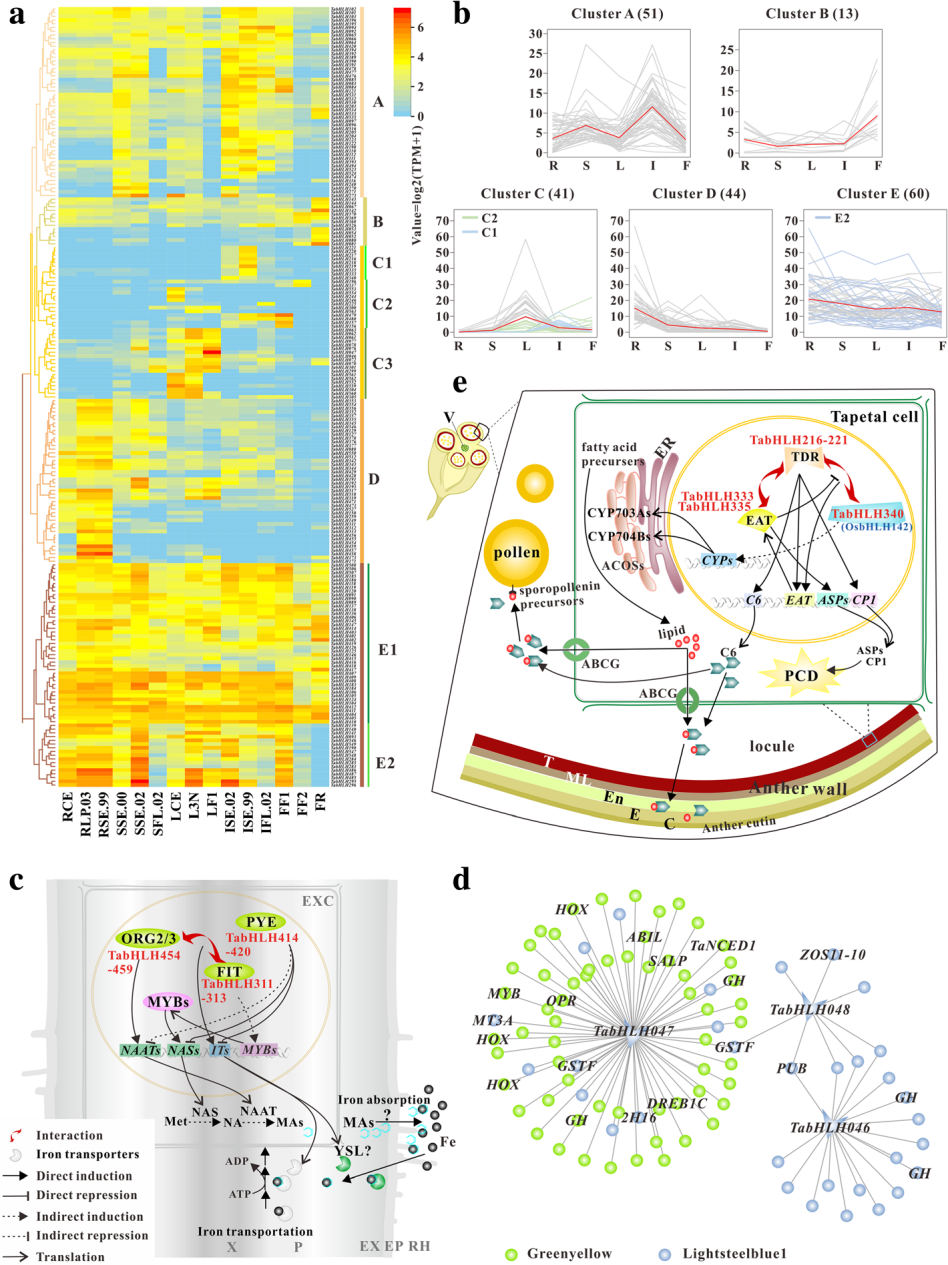
Expression profiles of *TabHLHs* in five organs at three developmental stages. **a** Hierarchical cluster analysis of *TabHLH* gene expression in 15 tissues. **b** Expression profiles of five clusters in five organs (R, root; S, steam; L, leaf; I, inflorescence; F, fruit) are shown. The gray, light-green and light-blue lines represent the expression changes of genes, and the red lines represent the mean expression trend of bHLHs belonging to each cluster. **c** Proposed model for the roles of several TabHLHs in Fe uptake. X, xylem; P, phloem; EX, exodermis; EP, epidermis; RH, rhizosphere. **d** The co-expression subnetwork constructed using *TabHLH046–048* as guide genes in modules “Lightsteelblue1” and “Greenyellow” with edge weight > 0.25. Other genes (except for *TaNCED1*) in the subnetwork are labeled according to their homologous genes in rice. GH, glycosyl hydrolase; GSTF, glutathione S-transferase; OPR, 12-oxophytodienoate reductase; SALP stress associated little protein; ZOS10–11, zinc-finger transcription factor; ABIL, A type 2C protein phosphatase; HOX, Homeobox-leucine zipper protein; MT3A, type 3 metallolthionein isoform; PUB, TPR and U-box domain containing protein. **e** Proposed model for the roles of TabHLHs in anther development. V, vascular bundle; T, tapetum layer; ML, middle layer; En, endothecium; E, epidermis; C, cuticular wax and cutin; ER, endoplasmic reticulum

To investigate the gene expression alterations in the development of bread wheat, deep transcriptome sequencing was performed in duplicates in 15 samples corresponding to five different organs (root, stem, leaf, spike and grain) at three development stages each [44]. After removing reads with low-quality, a total of about 2.82 billion paired-end reads were generated, with average of 93.56 million filtered reads for each library, as indicated in Additional file 13: Table S7. Eventually, approximately 96.08% of the reads were mapped onto the bread wheat genome, of which 79.46% were mapped uniquely in each library. In our study, 209 *TabHLH*s with expression values greater than 10 TPM (transcripts per million) in one or more tissues were selected for expression analysis (Additional file 14: Table S8). A total of 188 *ZmbHLH*s and 110 *OsbHLH*s were used for comparative analysis, which were expressed at medium and high levels (FPKM ≥5) in one or more tissues (Additional files 15 and 16: Tables S9 and S10). These wheat, maize and rice *bHLH*s were respectively grouped into five (A to E), four (F to I) and five (J to N) clusters according to the hierarchical clustering of gene expression data (Fig. 3a, Additional files 17 and 18: Figures S7 and S8). The 51 *TabHLH*s in cluster A were expressed with intermediate levels in stem at elongation stages, inflorescence at each stage and grain at early formation stage, particularly, *TabHLH273*, *− 094*, *− 084* and *− 121.* And some genes with very low expression values were found in stem and leaf at different reproductive stages. Cluster B consists of 13 members, of which eight had the highest expression levels in grain at ripening stage, such as *TabHLH142, − 081* and *− 143*. Cluster C with 41 genes can be further divided into three subclusters, of which nine in cluster C1 exhibited inflorescence-specific expression. In cluster C2, *TabHLH553, − 554*, *− 244* and *− 246* presented a relatively high expression levels in leaf at cotyledon emergence stage, while four genes *TabHLH479*, *− 480*, *− 156*, *− 157* were highly expressed in grain at early formation stage. And *TabHLH337* was specifically expressed in the grains at filling stages, which may be a homologue of endosperm-specific *ZHOUPI*s of Arabidopsis and maize. Several *ZmbHLH*s in cluster G were intensely expressed in embryo (*ZmbHLH090* and − *161*), endosperm and seed (*ZmbHLH139* (*ZmZHOUPI*), − *093* (*ZmmICE1*) and − *094*). *OsbHLH144* and − *001* respectively homologous to *ZmZHOUPI* and *ZmmICE1* were highly expressed in seed, which belong to cluster M where several genes showed higher transcript levels in panicle (*OsbHLH170*, *− 091*, *− 157* and *− 095*) and seed (*OsbHLH177*, *− 002* and − *147*). However, *OsbHLH160–162* and − *166* sharing high sequence similarities to *ZmbHLH161* showed root-specific expression. Members in cluster C3 showed higher transcript abundance in leaf than in other organs, including eight predicted *PIFs/PILs* (*TabHLH061–063*, *− 070*, − *072* and *− 076-078*) in subfamily VII(a + b), five (*TabHLH552* and *− 559-562*) in subfamily Ib(2), four (*TabHLH299*, *− 301*, − *304* and *− 305*) in subfamily III(a + c) and two (*TabHLH047* and *− 046*) in subfamily VIIIb. *OsBU1*/*ILI4* (*OsbHLH172*) was reported to function in controlling rice lamina inclination [45], while mainly expressed in callues in our analysis, which belongs to cluster K that comprises of callus-specific genes, such as *OsbHLH035*, − *042* and − *047*. Genes in cluster D were expressed with high levels in root, especially for *TabHLH454*–*459* and − *311*–*313*. *ZmHLH155* and *ZmbHLH101*, showing high sequence similarities to *TabHLH455* and *TabHLH312* respectively, belong to cluster I which contained a large number of tissue-preferentially expressed genes, such as *ZmHLH057* and *− 058* in germinating seed and embryo, *ZmbHLH096, − 110, − 111, − 121, − 130, − 145, − 146, − 149, − 150* and *− 206* in leaf, *ZmbHLH138* in seed and *ZmbHLH081*, − *099*, − *100*, − *117*, − *120*, − *123*, − *125*, − *180*, − *188*, − *208*, − *209* and − *222* in root. Whereas, *OsIRO2* (*OsbHLH056*), the rice homologue of *TabHLH454*–*459*, was strongly expressed not only in root but also in shoot. All genes in cluster E exhibited relatively high transcript abundance in each organ, with different expression patterns during development stages. Most *TabHLH*s within the subfamilies IVb and IVc were found in subcluster E1. In contrast, each *ZmbHLH* (except *ZmbHLH169* in cluster H) and *OsbHLH* in the two subfamilies respectively fell into clusters F and J in which genes were widely expressed in most tissues. As a member of subfamily IVb, *OsIRO3* (*OsbHLH063*) was expressed at higher levels in shoot and root than in other tissues. Interestingly, 17 genes in subcluster E2 were expressed at very low levels in grain at ripening stage, e.g., *TabHLH295*, − *296* and − *485*-*487*. Most of *ZmbHLH*s in cluster H were expressed at higher levels in vegetative organs than in reproductive organs.

### Expression profiles of *TabHLH*s, *ZmbHLH*s and *OsbHLH*s under drought stress

**Fig. 4:**
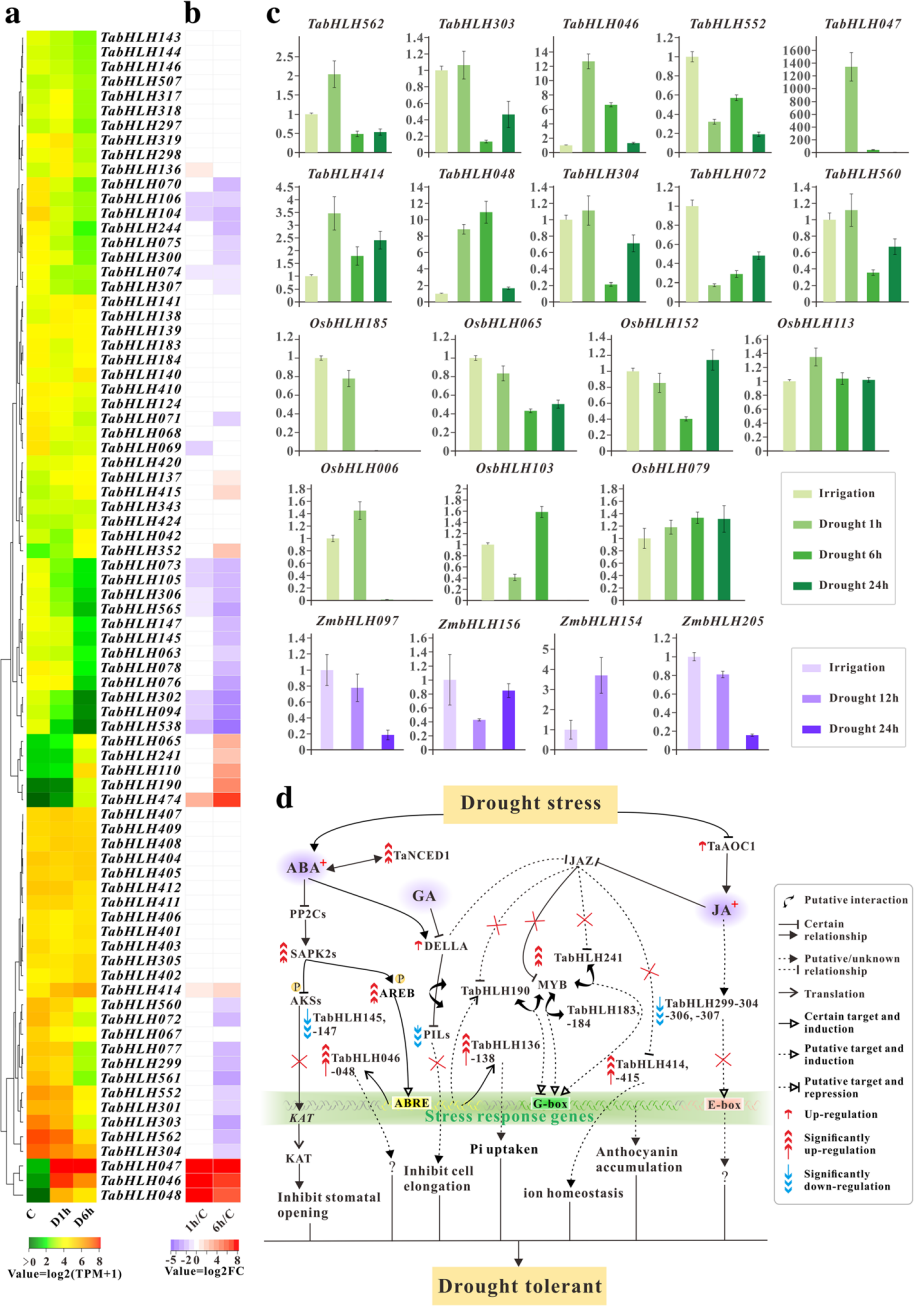
Expression profiles of *TabHLH*s under drought stress. **a** Heatmap shows expression profile of *TabHLHs* in drought tress and irrigation (**c**) conditions. **b** Heatmap presents statistically significant fold changes (log2-tranformed) calculated between each drought stress and irrigation condition. **c** qPCR analysis of selected genes in drought tress and irrigation conditions. **d** Proposed model for the role of several TabHLHs in response to drought

In this study, we focused on 80 *TabHLH*s that were expressed at levels above 10 TPM in one or more conditions, out of which 45 were identified as differentially expressed genes (DEGs) with fold change ≥2 and adjusted *P* values ≤0.05 in at least one condition compared to control (Fig. 4a, and Additional file 19: Table S11). Thirty two (including 11 for D1h, and 31 for D6h) of the 45 DEGs in wheat were significantly down-regulated, and the rest (including 6 for D1h, and 12 for D6h) were up-regulated under drought stress, as shown in Fig. 4b. While 78 *ZmbHLH*s expressed at levels ≥5 FPKM in at least one sample were selected for downstream analysis, of which eight and 20 were significantly affected by ≥2 folds down-regulation and up-regulation, respectively (Additional file 20: Table S12). In rice, 59 *bHLH*s were chosen for subsequent analysis due to their expression levels greater than 5 RPKM, of which 31 were considered as DEGs (Additional file 21: Table S13) including 11 down-regulated and 20 up-regulated genes. The transcript abundance of *TabHLH562*, *− 552*, − *304* and *− 303* were relatively high in control group and gradually decreased along with the enhancement of drought degree. Intriguingly, *ZmbHLH205* and − *206* that are likely to be homologs of *TabHLH562* and − *552* were up-regulated under drought stress. *ZmbHLH097*, − *098* and *OsbHLH006* (*OsRERJ*) that belong to the same subfamily III(a + c) along with *TabHLH304* and − *303* were up-regulated also. Several members of subfamily VII(a + b), *TabHLH063* and *− 069-078*, were significantly down-regulated, only *TabHLH065* was up-regulated. Analogously, *ZmbHLH054* and *OsbHLH109* orthologous to *TabHLH065* were up-regulated whereas others (especially *ZmbHLH051*, − *059,* and *OsbHLH104*, − *103*, − *102*, − *113* and − *152*) in this subfamily were down-regulated. *TabHLH047*, *− 046* and *− 048* were strongly up-regulated after drought stress treatment, and their rice homolog *OsqRT9* (*OsbHLH120*) showed increased expression as well. It’s worth noting that MYC-like genes *ZmbHLH103*, − *104* and *OsMYC2* (*OsbHLH009*) were up-regulated, which are different from *TabHLH183* and − *184* showing relatively stable expression. Additionally, *ZmbHLH155* and *− 154* expressions were inhibited, and levels of *ZmbHLH156* and its rice homologues *OsbHLH148* and − *185* were significantly increased.

The expression patterns of several selected *TabHLH*s, *OsbHLH*s and *ZmbHLH*s were validated by qPCR analysis. The primers used in this analysis were listed in Additional file 22: Table S14. Figure 4c showed that *TabHLH046*, *− 047*, *− 048, − 414* and *− 562* were rapidly up regulated within 1 h of drought stress, and *TabHLH562*, *− 552*, *− 303*, *− 304*, *− 072* and *− 560* were down regulated after 6 h of drought treatment. *TabHLHs* expression patterns tested in this assay consistent with that found in RNA-seq data. *OsbHLH006*, *− 185*, − *152* and *− 065* were significantly down-regulated after 6 h of drought treatment, while *OsbHLH113* did not show significant decrease in mRNA levels. Lower transcription abundances of *ZmbHLH205*, *− 097* and − *156* were detected 12 h after stopping the daily watering, and *ZmbHLH154* were significantly up-regulated.

### Expression profiles of *TabHLH*s and *ZmbHLH*s during fungal infection

**Fig. 5:**
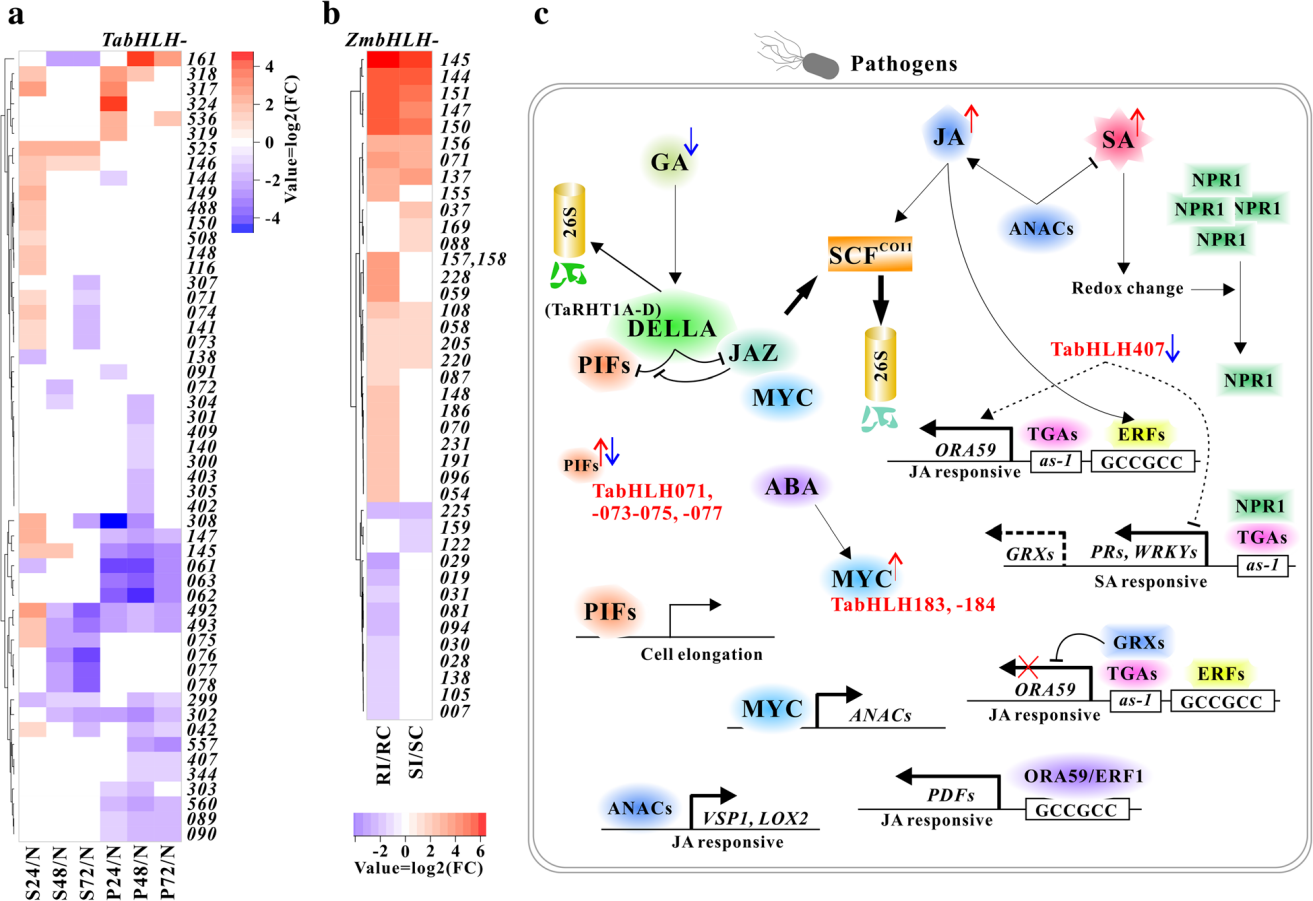
Expression profiles of *TabHLH*s and *ZmbHLHs* under different fungal stresses. **a** Heatmap depicts statistically significant fold changes (log2-tranformed) of expression after *Bgt* and *Pst* inoculation in wheat. **b** Heatmap illustrates statistically significant fold changes (log2-tranformed) of expression after *F. verticillioides* infection in resistant and susceptible maize genotypes. RI and SI represent the resistant and susceptible infection groups respectively; RC and SC represent the resistant and susceptible control groups, respectively. **c** Proposed model for the role of several TabHLHs in JA, SA, ABA and GA signaling pathways in response to pathogen stress

Stripe rust (*Puccinia striiformis* f. sp. *tritici*; *Pst*) and powdery mildew (*Blumeria graminis* f. sp. *tritici; Bgt*) are devastating diseases of wheat (*Triticum aestivum*). An RNA-Seq experiment with three biological replicates in each of seven conditions was performed, and *Pst* and *Bgt* fungus-inoculated wheat leaves were collected at 0, 24, 48, and 72 h post-inoculation (hpi) [46]. *Fusarium verticillioides* causes ear rot in maize and accumulation of mycotoxins affecting human and animal health. A deep sequencing data was generated for *F. verticillioides* inoculated and uninoculated resistant CO441 and susceptible CO354 maize genotypes at 72 hpi to study transcriptional changes [47]. To explore the roles of maize and wheat bHLHs in the stress responses of the fungal pathogens, the raw sequencing data was processed as described in method. A total of 85 *TabHLH*s were singled out for detailed analysis, which were expressed with levels ≥10 TPM in one or more conditions (Fig. 5a and Additional file 23: Table S15). And 223 RefGen_v3 IDs of *ZmbHLH*s were converted into RefGen_v4 IDs by the Maize Inflorescence Project (http://www.maizeinflorescence.org/v4/convert/index.php), of which 147 genes with expression levels ≥5 RPKM in one or more conditions were selected for further analysis (Additional files 24 and 25: Tables S16 and S17). As illustrated in Fig. 5a and b, our count-based differential expression analysis showed that 53 *TabHLH*s and 41 *ZmbHLH*s were significantly differentially expressed. Twenty two *TabHLH*s were up-regulated under *Pst* attack at 24 h, whereas nine of them decreased their expression levels at 72 hpi. To name but a few, the transcript abundance of *TabHLH492* and − *493* showed nearly 5.0- and 3.0-fold increase at 24 hpi, respectively, and then all decreased to less than the normal levels at 48 and 72 hpi. Under *Bgt*-induced stress, six genes were up-expressed, and 28 were down-expressed. Interestingly, *TabHLH144, − 145* and *− 147* were up-expressed under stripe rust stress and down-expressed under powdery mildew stress, whereas *TabHLH161* exhibited opposite expression pattern. Four genes (*TabHLH304*, *− 299*, *− 061* and *− 302*) were down-regulated and two (*TabHLH317* and *− 318*) were up-regulated in response to both *Bgt*- and *Pst*-induced stress. The transcript levels of *ZmbHLH144*, − *145*, − *147*, − *148*, − *150* and − *151* homologous to *TabHLH317* and *− 318* increased up to 4.39- to 86.74-fold in two genotypes. Of another 23 up-regulated *ZmbHLH*s, 12 genes were significantly induced in resistant genotype, such as, *ZmbHLH096*, *− 155*, *− 054* and *− 059*, and three (*ZmbHLH037*, *− 169* and *− 088*) were found in susceptible line. The remaining 13 genes were down-regulated, of which three (*ZmbHLH225*, *− 159* and *− 122*) were found in the susceptible. In addition, *TabHLH071–078* belonging to PIF-like subfamily were significantly induced by *Pst* infection at 24 hpi and repressed at 48 hpi, while gradually up-regulated in response to *Bgt* infection, particularly *TabHLH077*. Similarly, *ZmbHLH058*, − *059* and − *054* in the subfamily were induced remarkably in the resistant line.

### Gene co-expression module generation and functional enrichment analysis

**Fig. 6:**
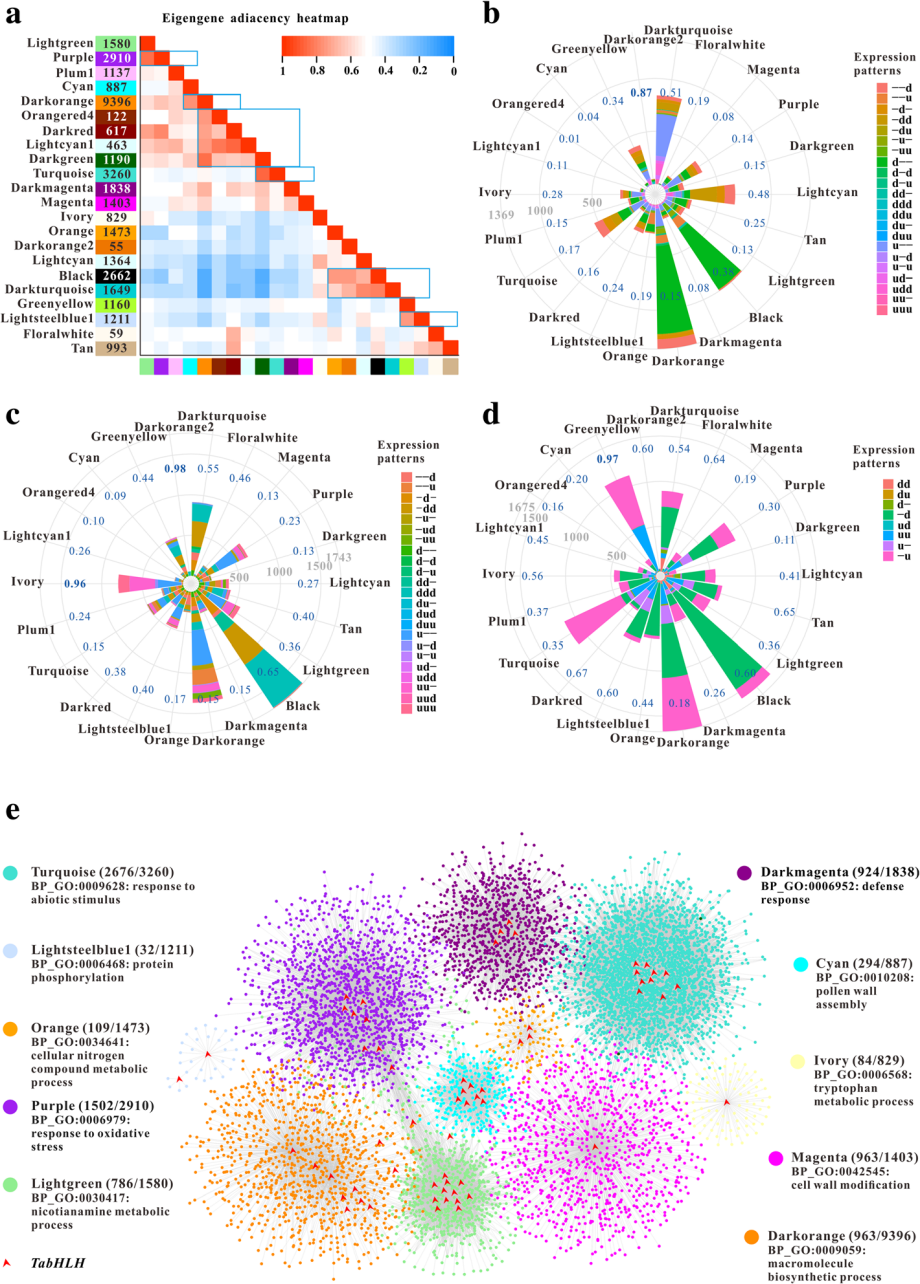
Weighted gene co-expression network analysis of wheat genes. **a** Heatmap of eigengene adjacencies. Blue represents negative correlation and red represents a positive correlation. The colored box on the left side of the heatmap illustrates the corresponding module and gene number. **b-d** Nightingale rose diagrams show the distribution of DEGs of each module under stripe rust, powdery mildew and drought stresses. The sectors with different colors indicate the distinct expression patterns over time after stress treatment. The larger the radius of a sector, the greater number of DEGs is included. “-”, non-significant; “d”, significant down-regulation; “u”, significant up-regulation. The deep-blue number indicates the percentage of differentially expressed genes in corresponding module. **e** Ten co-expression subnetworks constructed using several *TabHLHs* as guide genes. The red notes represent *TabHLHs.* The numbers in bracket correspond to genes annotated for the top GO term and genes in the module

Recently, co-expression network analysis became an effective approach for gene functional annotations [48]. In this study, we performed WGCNA which introduces a soft-threshold method. According to approximate scale free-topology criterion, a suitable soft-threshold value of 12 was employed to construct gene co-expression modules (Additional file 26: Figure S9). A total of 36,258 genes were parsed into 22 gene modules ranging from 55 (darkorange2) to 9396 (darkorange) genes and represented by color classifiers (Additional file 27: Figure S10). As shown in Fig. 6a, some modules share high-positive correlation, such as cyan and darkorange, greenyellow and lightsteelblue1, darkgreen and turquoise. Considering high volume data, GO enrichment analysis were used to investigate the functions of co-expressed genes within a module (Additional file 28: Table S18). Cyan module contains anther-specific terms associated with pollen wall assembly, pollen exine formation, sporopollenin biosynthetic and fatty acid metabolic processes (GO:0010208, GO:0010584, GO:0080110 and GO:0006631). Lightgreen module reflects gene functions related with nicotianamine metabolic, nicotianamine biosynthetic and tricarboxylic acid biosynthetic processes (GO:0030417, GO:0030418 and GO:0072351). For lightcyan module, significant terms were enriched in phosphorus metabolic process, protein modification and phosphorylation process (GO:0006793, GO:0036211 and GO:0016310). Then the module-tissue correlation analysis was conducted (Additional file 27: Figure S10). Cyan module is significantly correlated with inflorescence at the maximum stem length reached stage (ISE.99). The lightgreen module show higher correlation to the development of root than that of others. The correlation coefficients between turquoise module and each tissue (expect FR, fruit at whole plant fruit ripening stage) are significantly high, ranging from 0.39 to 0.53. Further GO enrichment analysis showed the genes in this module are implicated not only in the response to abiotic and biotic stresses (GO:0009628 and GO:0006952) but also in various biological functions. As is evident from the Nightingale Rose Diagrams, darkorange and black modules contain the largest numbers of DEGs with different expression patterns during *Pst* and *Bgt* infection and drought stress (Fig. 6b-d). However, the percentages of DEGs within darkorange2, ivory and greenyellow modules were higher than those in others. Both tryptophan metabolic and indolalkylamine metabolic processes (GO:0006568 and GO:0006586), which contribute to the capacity for chemical defense against microbes [49], are the significantly enriched GO terms for genes in ivory module. Greenyellow module showed significant enrichment of water, inorganic substance, oxygen-containing compound and abiotic stimulus responses (GO:0009415, GO:0010035, GO:1901700, and GO:0009628). To further understand the biological roles of *TabHLH*s, ten subnetworks composed of guide genes (*bHLH*s) along with their first-degree neighbors with edge weight ≥ 0.4 were extracted (Fig. 6e), which are associated with each other possibly due to a common biological process. The general function of these co-expressed modules was given according to the most enriched GO term and *P* value (Additional file 29: Table S19).

## Discussion

### Comparative evolutionary analysis of *bHLH*s

The gene and protein characteristics, expression patterns and function of some *bHLH*s have already been unfolded in many plants, whereas the relative research has still not been performed in maize and wheat, two of the most important crops worldwide. The genome sequencing projects in wheat are still in infancy compared to other plants, and currently available wheat genome sequences are relatively fragmented [50], which makes it difficult to study tandem and segmental gene duplication events during the evolution of a gene family. In this study, based on the latest draft genome sequence of hexaploid bread wheat, we systematically identified the bHLH gene family including 571 members (Additional file 1: Table S1). The percentage of the number of *bHLH* genes in wheat protein-coding genes was only higher than that in rice. Comparative evolutionary analyses among four species, including phylogenetic tree reconstruction, gene structure investigation and conserved motif detection, served as the first step in a comprehensive functional characterization of bHLH transcription factors.

Previous phylogenetic analyses of plant bHLH gene family provided a helpful phylogenetic framework for the classification of *bHLH*s*,* but it varied between different studies, which probably due to different methods and sequences adopted [25, 26]. Based on the phylogenetic tree generated, *bHLH*s in Arabidopsis, rice, maize and wheat can be classified into 36 subfamilies, of which 28 subfamilies include genes from the four species and eight subfamilies (40 genes) are likely to be monocotyledon-specific. Some subfamilies clustered together with a high bootstrap probability (> 0.5) in the tree constructed with the sequences of bHLH domains from four species among the 36 subfamilies were not assigned to a subgroup, such as IVb and IVc, XI and XII, VIIIc(1) and VIIIc(2) due to their distinct gene structures and protein motifs in full-length sequences (Additional files 7, 8 and 10: Figures S3-S5). In the phylogenetic tree of maize *bHLH* genes, subfamilies 26, 28 and 30–34 clustered with a high bootstrap probability, while in that of wheat only 26–28 and 31 subfamilies clustered together. And some “orphan” fell into one subfamily in phylogenetic tree constructed for single species, such as *TabHLH505*, *ZmbHLH140*, *ZmbHLH228* and *ZmbHLH230*. From the results, we speculated that the high degree of sequence divergence of *bHLH*s is presented in the four species probably due to species-specific specializations. And a lot of small clusters having exactly one representative member from each subgenome were regarded as homologous triplets (Additional file 5: Figure S2).

The members with conserved non-bHLH motifs might play a similar role. The MIR in the N-terminal region of MYC-like bHLHs is responsible for interaction with R2R3-MYB proteins [51]. By interacting with MYB proteins, three MIR containing proteins AtGL3, AtEGL3 and AtTT8 acted in a partially redundant manner to specify root and leaf epidermal cell fates and mediate JA-induced anthocyanin accumulation [52–54]. In addition, AtMYC2, AtMYC3, and AtMYC4 can interact directly with GS-related MYBs to promote glucosinolate (GS) biosynthesis gene expression in response to JA-related defense [55]. Several MYC-like *bHLH*s have been reported in Arabidopsis, rice, and maize, but none was studied in wheat. Here were 33 *TabHLH*s in subfamilies III(d + e), IIIf and XIII considered as MYC-like *bHLH*s based on protein sequence analyses. Heterodimer formed by AtbHLH156/LHW in subfamily XIII and AtbHLH032/TOM5 or AtbHLH030/T5 L1 or SACLs (AtbHLH142–145) coupling with auxin and cytokinin signaling pathways participates in the regulation of Arabidopsis root vascular initial population [56]. bHLHs have multiple functions due to the complex structural characteristics, and future experimental evidence should help to determine whether they can interact with MYBs.

### Spatio-temporal expression dynamics of *bHLH*s during development

In this study, the expression patterns of *bHLH*s in different tissues and developmental stages were systematically investigated to understand their potential function during the life cycle of rice, maize and wheat. As seen in Fig. 3b, Additional files 17 and 18: Figures S7 and S8, most of genes were expressed in specific organ. Several genes in cluster D were selectively expressed at high levels in root. Among them, *TabHLH454–459* have the high sequence similarity with *OsIRO2* which positively controlled the expression of key genes involved in Fe absorption, such as *OsNAS1*, *OsNAS2*, *OsNAAT1* and *OsYSL15* [21]. The relatively high-abundance transcript levels of *ZmbHLH155* homologous to *OsIRO2* were observed in root, stem and leaf. The above mentioned *bHLH*s display high degree of sequence similarity to four *AtbHLH* genes which encode AtbHLH038 (ORG2), AtbHLH039 (ORG3), AtbHLH100 and AtbHLH101 that functioned downstream of AtbHLH034-AtbHLH104-AtbHLH105 complex and separately interacted with FIT1 (AtbHLH029) to constitutively regulate the expression of *IRT1* (high-affinity ferrous iron transporter) and *FRO2* (ferric–chelate reductase) [57]. *TabHLH311*–*313* and *ZmbHLH099–101*, homologs of *FIT1*, were expressed with high levels in root as well, whereas twelve *TabHLH*s (*TabHLH401*–*412*) and six *ZmbHLH*s (*ZmbHLH173*–*178*), homologs of *AtbHLH105* (*ILR3*), were widely expressed in various organs including root. Moreover, *TabHLH454*–*459* and − *311*–*313* were in lightgreen module that contains a number of genes involved in nicotianamine (NA) biosynthesis. NA constitutes the biosynthetic precursor of MAs that efficiently bind Fe(III) in rhizosphere, contributing to metal transport in graminaceous plant species. We identified 31 NA synthase encoding genes (*NAS*s), of which 18 were discovered in wheat genome assembly IWGSC1.0 by Julien Bonneau et al. [58], and 24 out of 31 were presented in the subnetwork constructed using these *bHLH*s (*TabHLH171*, − *311*–*313*, and − *454*-*459*) as guide genes (Additional file 30: Table S20). Six putative nicotianamine aminotransferase (NAAT) and three iron(III)-deoxymugineic acid transporter (YSL) encoding genes were also found in the subnetwork. In IVb subfamily, seven *TabHLH*s (*TabHLH414–420*) and four *ZmbHLH*s (*ZmbHLH169–172*) have higher sequence homologies to *AtPYE* (*AtbHLH047*) and *OsIRO3* that can negatively regulate the transcription of several metal absorption-related genes [59, 60], and are expressed in all organs, and only *TabHLH415* and *− 416* were found in lightgreen module. These results suggested that some *bHLH*s might participate in the regulation of metal absorption and homeostasis in root and other organs (Fig. 3c). Additionally, *ZmbHLH180*, *TabHLH471* and − *473* specifically expressed in root show homologies with *AtPRE1*/*TMO7*, *OsILI3* and *OsILI7* which may be involved in the regulation of cell elongation in response to BR, GA and auxin signals [61, 62]. Both *TabHLH047* and − *046* exhibiting significant sequence similarities to *AtHEC*s (*AtbHLH088*, *− 037* and *− 043*) and *OsqRT9* were relatively high expressed in leaf, and our co-expression network analysis showed that the former might be directly connected to the expression of *TaNCED1* homologous to *OsNCED3* involved in ABA biosyntheses, shaping leaf morphology and vascular bundle development (Fig. 3d) [63]. *ZmbHLH064* and − *065*, putative maize homologues of *AtHEC*s, were expressed in leaf and primary root. Earlier researches found that AtHECs can dimerize with SPT (AtbHLH024, belonging to subfamily VII(a + b)) to coordinately regulate gynoecium development and control stem cell fate by repressing the stem cell regulators WUS and CLAVATA3 (CLV3) [64, 65]. A recent study reported that AtHECs can not only interact with PIFs to fine-tune photomorphogenesis but also positively regulate cholorophyll and carotenoid biosynthesis [66]. Although eight putative *TaPIF*s/*PIL*s were expressed with relatively high levels in leaf, they were not presented in lightsteelblue1 module.

*TabHLH216*–*221*, orthologues of *OsTDR1* (*OsbHLH005*) that is a key regulator of tapetum development and degeneration by promoting the transcription of *OsCP1* and *OsC6* [67], showed inflorescence-specific expression. It was reported that OsEAT1 (OsbHLH141) acted downstream of OsTDR1 and regulated the expressions of *OsAP25* and *OsAP37* which encode aspartic proteases participating in tapetal programmed cell death (PCD) [68]. *TabHLH333* and *− 335*, sharing sequence similarity to *OsEAT1*, were also specifically expressed in inflorescence at maximum stem length reached stage. Additionally, *TabHLH340*, a homologue of *OsbHLH142* encoding a protein that can interact with OsTDR1 to regulate the expression of *OsEAT1* and be directly connected to the expressions of *OsCYP703A3*, *OsCYP704B2*, *OsMS2* and *OsC6* [9], was specifically expressed in inflorescence. From homology-based analysis of downstream-regulated genes *OsCP1*, *OsC6*, *OsAP25*, *OsAP37*, *OsCYP703A3*, *OsCYP704B2*, *OsMS2*, *OsACOS12* and *OsABCG15* which are required for regulating tapetal PCD and biosynthesis of sporopollenin and cutin precursors, we found that all of them have orthologs in wheat*.* Most of them showed inflorescence-specific expression, and were presented in cyan subnetwork (Additional file 30: Table S20). Hence, we speculate that *TabHLH216–221*, *− 333, − 335* and *− 340* may play essential roles in anther development (Fig. 3e). *TabHLH479*, *− 480, − 156* and *− 157* were expressed with significant levels not only in inflorescence but also in grain at early formation stage. The first two genes show homologies with *OsBU1/ILI4* (*OsbHLH172*) and *OsPGL2/ILI5* (*OsbHLH170*) that controlled bending of the lamina joint and regulated the grain length and weight [45, 69], and their maize homolog *ZmbHLH184* was highly expressed in immature leaf and developing seed. Our gene co-expression network analysis showed that *TabHLH479* and − *480* highly expressed in fruit formation stage (FF1) were directly connected to alpha-amylase- (TaAMY3.1–4), starch synthase- (TaSSIIa1–3 and TaGBSS1–3) and aldose 1-epimerase- (TaA1E1–4) encoding genes and so forth. *TabHLH142*, *− 081*, *− 052-054* and *− 080* were expressed significantly during grain ripening stage and directly connected to lots of genes encoding organic cation/carnitine transporters (TaOCT1–5), sugar transporters (TaGMST1–2 and TaSWEET1) and late embryogenesis abundant proteins (TaLEA1–26) in turquoise module. Each of them contains Skn-1 motif required for endosperm expression in their promoter regions, and RY-element involved in seed-specific regulation is present in *TabHLH081* and − *080*. Outstandingly, *ZmbHLH058*, an orthologue of *TabHLH081*, − *080*, *AtPIF4* and *AtPIF5* were expressed with extremely high levels in embryo and germinating seed. A series of experiments revealed that PIF4 can facilitate hypocotyl elongation through activating auxin biosynthesis gene *YUCCA8* and signaling gene *IAA29* in Arabidopsis [70, 71].

### Functional divergence of *bHLH*s in response to drought stress

The crops are often exposed drought conditions which drastically affect the growth and development through distressing various biochemical and physiological processes. Accumulating evidences suggested that *bHLH*s were involved in drought stress response [13, 72, 73], however the roles of *TabHLH*s and *ZmbHLH*s have scarcely been reported in response to water deficiency. In our study, 45 *TabHLH*s*,* 29 *ZmbHLH*s and 31 *OsbHLH*s were found to be differentially expressed under drought treatment. Drought responses are coordinated by complex signaling networks, and ABA acts as a global regulator. Recent study suggested that *TabHLH136* (TC307165) overexpression improved the tobacco growth and osmotic stress-associated traits through modulating ABA signaling [18], and overexpression of its homologue *OsPTF1* (*OsbHLH096*) in rice may increase root length and surface area resulting in higher instantaneous Pi uptake rate [20]. Putative homeologous triplet *TabHLH136–138* were up-regulated at 1 h after 20% PEG-6000 treatment, however only *TabHLH136* with ABRE in the promoter returned to normal levels at 6 h. And transcripts of *ZmbHLH047* orthologous to *TabHLH136–138* and *OsPTF1* were significantly increased by approximately 3.0-fold under drought treatment. The activation of AKS1/CFLAP1/FBH3 (AtbHLH122), AKS2 (AtbHLH128) and AKS3/FBH4 (AtbHLH130) was suppressed by ABA-induced phosphorylation, decreasing K+ channel (KAT1) expression in guard cells leading to stomatal closure [74]. *TabHLH145, − 147* and *OsbHLH143* homologous to *AtAKS*s were significantly down-regulated. Interestingly, there was a significant decrease in the expression levels of some of the genes after 6 h when compared to 1 h but still remained higher than the non stressed controls, such as *TabHLH048*, *− 303*, *− 304* and − *560*. While qPCR assays showed that mRNA accumulation of *TabHLH047* rather than *TabHLH048* was significantly reduced at 6 h. Coexpression analysis indicated that *TabHLH047* was directly linked to several stress-responsive genes encoding TaNCED1, homeobox-leucine zipper proteins (TaHOX22_A, TaHOX22_B and TaHOX22_D), glutathione S-transferases (TaGSTF1–2), glycosyl hydrolases (TaGH1–3) and 12-oxophytodienoate reductase (TaOPR1) and so on (Fig. 3e, Additional file 30: Table S20). *TabHLH190* and *ZmbHLH108*, the homologues of *AtJAM2* (*AtbHLH013*) which functioned mostly antagonistically to *AtMYC2* in JA signaling pathway [75], were differentially up-regulated. *ZmbHLH103* and *− 104* homologous to *AtMYC2* were significantly up-regulated, whereas the expressions of wheat homologs (*TabHLH183* and *− 184*) remained relatively constant across different sampling times. In contrast, *TabHLH241* having high sequence similarity to *AtEGL1/EGL3* which participated in JA-mediated anthocyanin accumulation [54], was up-regulated at 6 h, while its maize homologs *ZmbHLH124* and − *125* were expressed at very low levels. According to the above expression analysis combined with the data listed in Additional file 1: Table S1, we speculated that TabHLH190, − 241, ZmbHLH103 and − 104 might bind G-box element of downstream drought-responsive genes and regulate their transcriptions. The expression levels of *TabHLH414*, − *415* and *ZmbHLH171* were increased under drought stress, whose rice ortholog *OsbHLH062* encodes a interactor of JAZ9 to regulate the transcriptional activation of JA-, salt stress-responsive genes including ion transporter genes [14]. The higher *OsRERJ* mRNA levels was detected 1 h after drought treatment and the abundance was decreased rapidly in rice seedlings thereafter, as previously reported [76]. Similarly, the expression patterns of its homologous *ZmbHLH097*, *TabHLH303* and *− 304* were observed under drought stress during seedling period. We speculated that their mRNA accumulations peaked less than 1 h after 20% PEG treatment and 12 h drought condition. However, high *ZmbHLH097* and *− 098* mRNA levels were detected in fertilized ovary and basal leaf meristem tissue under drought stress, about 3 days after irrigation withheld. In addition, our qPCR assay verified that *ZmbHLH154* was strongly up-regulated, which is different from its expression profile at reproductive stage. And several putative wheat and maize *PIF*s*/PIL*s were down-regulated, with the exception of *TabHLH065* and *ZmbHLH054* that are homologues of OsPIL16/APG which have been reported to be a negative regulator of the grain length and weight [77], suggesting a role in regulation of the balance of growth and drought stress response. This analysis indicated some *bHLH*s may be associated with drought stress response through diverse regulation processes in different tissues. And it was a major challenge to analyse the mechanism of genes transcriptional regulation in response to water deficiency at different development stages.

### Involvement in immune responses against pathogens

In the expression analyses, we observed a differential response of *TabHLH*s for *Pst* and *Bgt* pathogens though both of them are biotrophic fungi (Fig. 5). Salicylic acid (SA) play a central role in the disease resistance against biotrophic pathogens, while JA is critical for activation of defense against necrotrophic pathogens [78]. SA- and JA-responsive signaling are interdependent, and SA antagonize JA signaling downstream of COI1, degrading ORA59 and then suppressing *PDF1.2* [79]. When SA levels are intermediate, NPR1 accumulates and interacts with TGA transcription factor, acting as a co-activator to activate SA responsive genes including pathogen-related protein (PR) genes [80]. One earlier study reported that *TabHLH407* (GenBank accession: ADC33137) overexpression resulted in the suppression of transcription levels of *PR1*, *PR2* and *PR5*, and the enhancement of the expression of *ORA59* (AP2/ERF domain transcription factor) and *PDF1.2* (*PLANT DEFENSIN1.2*) in transgenic Arabidopsis [23]. In our analysis, putatively homeologous triplet *TabHLH407*, *TabHLH408* and *− 409* were suppressed by *Bgt* and *Pst* attack. The transcripts of several *TaPR1*s (*TaPR1_1–4*, *TaPR1_14*, *TaPR1_18* and *TaPR1_20*) were highly abundant at 24 hpi and then gradually decreased following *Bgt* inoculation, and *TaPR1_1–4* had similar expression patterns after *Pst* infection (Additional file 30: Table S20). We identified 50 wheat homologues of *AtORA59*, of which five (*TaERF9*, − *16*, − *25*, − *33* and − *48*) were obviously induced by two pathogens and one (*TaERF31*) was differentially activated by *Bgt*. Of our identified 140 wheat defensin-like genes (*DEFL*s) using AtPDF1s and AtPDF2s sequences as queries for local BLAST search, three (*TaDEFL18*, − *19* and − *59*) were up-regulated after *Pst* incubation at 24 hpi, and one (*TaDEFL33*) was significantly induced by *Bgt* infection at 72 hpi (Additional file 31: Figure S11). *ZmbHLH174* sharing high sequence similarity with *TabHLH407* was down-regulated under *F. verticillioides* infection in resistant genotype, and *ZmPDF1* (LOC100280744) and *ZmPDF2* (LOC100280572) were intensely expressed in both genotypes. In comparison, normal mRNA expression levels in susceptible line are higher than those in resistant line. Conversely, higher fold changes were observed in resistant one. Interestingly, the mRNA levels of another five *TaDEFL*s (*TaDEFL57*, − *59* and − *80-82*) accumulated substantially in grain at ripening stage. As a transcriptional coactivator of TGAs in SA-mediated pathway, *TaNPR1_A1, TaNPR1_D1* and *TaNPR1_D2* were induced by the *Bgt*, which were not observed during *Pst* attack. MYC branch is another branch of JA signaling pathway, in which *AtMYC2* acts as the master regulator [81]. *TabHLH183* and *− 184* with ABRE-, CGTCA- and TGACG-motifs in promoter regions*,* the homologs of *OsMYC2* and *AtMYC2*, were slightly up-regulated. For their homologous genes in maize, *ZmbHLH103* and − *104* expressions were increased by *F. verticillioides* infection. A series of evidences have substantiated that AtANAC019 and − 055 acted downstream of AtMYC2 and enhanced the expressions of *AtVSP1*, *AtLOX2* and *AtBSMT1* but suppressed *AtICS1* [82, 83]. Herein, five wheat homologues (*TaNAC1–5*) of *AtANAC019* and *− 055* were induced by *Pst* infection at 24 hpi and then decreased to very low levels, whereas the mRNA levels remained relatively stable after *Bst* invasion. Besides, *TaLOX3.1–3* were significantly induced by both pathogen, particularly *TaLOX3.3* at 24 hpi. JAZ and DELLA proteins mediate crosstalk between JA-dependent defenses and GA-triggered elongation growth. Under biotic stress, JA concentrations increase to high levels, resulting in the degradation of JAZs and the release of MYCs and DELLAs to activate JA-responsive genes and to bind PIFs, respectively. In this study, three *TaPIF*s/*PIL*s (*TabHLH071*, − *073* and − *075*) were up-expressed at 24 hpi after *Pst* infection and down-expressed at 72 hpi, with opposite expression patterns observed in *Bgt* infection. In addition, some *ZmPIF*s/*PIL*s were up-regulated, especially *ZmbHLH058*, − *059* and *ZmPIL1* (*ZmbHLH054*). *OsGAI* encoding DELLA protein was reported to integrate and amplify SA- and JA-dependent defense signaling [84]. Its homologous genes *TaRHT1A-D* were significantly up-regulated after *Pst* inoculation, especially at 24 hpi, but down-regulated after *Bgt* infection. Of our identified *TaJAZ*s (Additional files 30 and 32: Table S20 and Figure S12), six of 61 genes were induced under pathogen stress, including *TaJAZ14B*, −*15D*, −*16A*, -*17B*, -*9B* and -*2B* homologous to *OsJAZ10*, − *11*, − *6*, − *8*, − *4* and *OsTIFY1a* respectively. In addition, strongly up-regulated *ZmbHLH144*, − *145* and *TabHLH317*–*319* are homologous with *OsDPF* (*OsbHLH025*) which is implicated in the biosynthesis of diterpenoid phytoalexins by positively regulating *CPS2* and *CYP99A2* [22]. And *TabHLH324* significantly induced after *Bgt* infection was closely connected to some genes listed in Additional file 30: Table S20, which encode CYPs, UDP-glucosyl transferases (UGTs), 2-oxoglutarate/Fe(II)-dependent dioxygenases (2-ODDs) and Tryptophan synthases (TSAs) and may be involved in the biosynthesis of DIMBOA and tryptophan. Our results indicated that bHLHs are crucial components of a complex regulation circuit involved in the plant disease resistance. Due to the lack of genome-scale identification of PR, AP2/ERF, PDF, bZIP, NPR, and other gene families for the updated wheat genome assembly, the current study has a few limitations to study the signaling pathway in response to pathogen stresses.

## Conclusions

The updated genome assemblies are expected to serve as a platform for our comparative functional genomics studies. In this study, 183 *OsbHLH*s, 231 *ZmbHLH*s and 571 *TabHLH*s were identified. The comparative evolutionary analysis showed that all *bHLH*s from Arabidopsis, rice, maize and wheat can be divided into 36 subfamilies. The exon/intron structure and motif compositions were conserved within one subfamily. Some *bHLH*s were specifically expressed in endosperm, grain, inflorescence, leaf, root, or shoot. Combining with the WGCNA and GO function enrichment analysis, we speculated that *TabHLH454–459* and − *311*–*313* may be related with iron uptake, and *TabHLH216*–*221*, *− 333*, *− 335*, and − *340* may be involved in anther development. Some differentially expressed *bHLH*s may be required for enhancing the tolerance of crop plants to drought and pathogen stresses. PIFs/PILs, MYCs and TabHLH407–409 and ZmbHLH174 may be involved in the crosstalk between GA-mediated growth and JA-, ABA-, SA-dependent defenses. And some bHLHs participate in the regulation of secondary metabolism in response to environmental stimulus. Comparative spatiotemporal expression analysis of lots of putative homeologous triplets showed that there was apparent expression divergence among the homeologs, but it was nonradical alterations, suggesting that the subfunctionalization rather than neo-functionalization occurred among three subgenomes. Our analysis will facilitate the future functional analysis of bHLHs in Gramineae species and contribute to molecular breeding for improving yield, stress tolerance and grain quality.

## Methods

### Database mining and identification of *bHLH* genes

Rice, maize and bread wheat genome sequences, annotation files in GTF format, and protein sequences were downloaded from TIGR (release 7), Ensembl Plants (release 31, http://plants.ensembl.org/index.html) and Ensembl Plants (relase 32), respectively. Three local protein databases were established with these protein sequences. As per the previous researches, predicted AtbHLHs, OsbHLHs and ZmbHLHs were retrieved from TAIR (release 10), TIGR and Ensembl Plants, respectively [25, 32, 35, 36]. As no typical bHLH domain was found in At1t22380 (AtbHLH152), Os07t11020 (OsbHLH017), Os01t65080 (OsbHLH033), Os01t18290 (OsbHLH105), Os04t35000 (OsbHLH145), GRMZM5G899865_T01 and GRMZM2G137426_T01, they were excluded from the study. All bHLH domain sequences from three species were aligned with MEGA 6.0 software (http://megasoftware.net/) and the obtained multiple alignments were used to construct Hidden Markov Model (HMM) profiles with hmmbuild tool implemented in HMMER3.1 (http://hmmer.org/). The profiles were used to search the local protein databases using hmmsearch tool. Then the bHLH HMM profile (accession number: PF00010) in the Pfam database (http://pfam.xfam.org/) was also applied to search the proteins. All non-redundant sequences were received a conserved domain check using Pfam tool and SMART web server (http://smart.embl-heidelberg.de/). To confirm the amino acid sequences as bHLHs, we examined them by counting the number of matches at each region of the predicted bHLH domain according to a low stringent criterion with allowing 12 mismatches from the HLH consensus.

### Phylogenetic analysis

Multiple alignments of identified bHLH domain sequences from 571 TabHLHs, 231 ZmbHLHs, and 183 OsbHLHs were created respectively using MAFFT v7 (http://mafft.cbrc.jp/alignment/software/) with --auto, −-reorder options. The bHLH sequences used for our analysis were renamed as TabHLHXXX, ZmbHLHXXX and OsbHLHXXX, and the number designation of them was based on the order of the multiple sequence alignment. To investigate the evolutionary relationships among Arabidopsis, rice, maize and wheat bHLHs, multiple sequence alignment of 1154 bHLH domains was generated using MAFFT. Phylogenetic tree was constructed by the neighbor-joining (NJ) method with a bootstrap test (1000 replicates) using MEGA v6.0. The alignments were visualized using ESPript v3.0 (http://espript.ibcp.fr/ESPript/ESPript/), and the tree image was generated using iTOL (http://itol.embl.de/).

### Gene structure analysis and conserved motif detection

The exon/intron organization and splicing phase of the predicted *TabHLH*s and *ZmbHLH*s were investigated based on the GTF annotation files of *Triticum aestivum* and *Zea mays* genomes, and then graphically displayed by the Gene Structure Display Server (GSDS, http://gsds.cbi.pku.edu.cn/) [85]. For *OsbHLH*s, the gene structures were determined by comparing predicted gene coding sequences (CDS) with their corresponding genomic sequences using GSDS. Furthermore, to discover the intron distribution pattern within the coding sequence of the bHLH domain, the different regions of bHLH domain were assigned to different colors. To analyze other conserved motifs in complete amino acid sequence of bHLHs, the MEME tool in Galaxy web-based platform (https://usegalaxy.org/) was used. And each motif was individually checked using FIMO tool in the platform also.

### *Cis*-regulatory elements analysis

The upstream 1500 bp genomic DNA sequences of predicted *TabHLH* genes were extracted from *Triticum aestivum* genome using TBtools (http://cj-chen.github.io/tbtools/), and then submitted to PlantCARE database (http://bioinformatics.psb.ugent.be/webtools/plantcare/html/) for scanning *cis*- regulatory elements.

### Read alignment and expression analysis

The raw sequencing data in FASTQ or SAM formats generated from wheat (*cv.* Chinese Spring, N9134 and TAM107) of different developmental stages and under different treatment conditions are available from ArrayExpress (http://www.ebi.ac.uk/arrayexpress/) with accession numbers E-MTAB-4484 and E-MTAB-4289, and NCBI Sequence Read Archive (SRA) database (https://www.ncbi.nlm.nih.gov/sra) with accession number SRP045409. With the TGACv1 wheat genome assembly and annotations generated in Dec. 2015, to which 99% genes annotated on the IWGSC1 + popseq assembly can be mapped, more coding genes can be identified. Therefore, we re-processed the raw data according to the following steps: The raw pair-end reads were quality-trimmed with multi-perspective approach via SeqPrep (https://github.com/jstjohn/SeqPrep), Sickle (https://github.com/najoshi/sickle) and Fastx-Toolkit (http://hannonlab.cshl.edu/fastx_toolkit/). Clean data of pair-end reads from each sample were aligned to the TGACv1 wheat genome assembly by HISAT2 software [86]. The raw count of unique mapped reads to each gene was aggregated using featureCounts and used to calculated TPM value utilizing a custom-made Perl script [87]. The DEseq2 package in R software (v3.3.1) (http://www.bioconductor.org/packages/devel/bioc/html/DESeq2.html) was used to perform differential gene expression analysis, and fold change cutoff of 2 and adjusted *P* value ≤0.05 were taken as statistically significant threshold. The log2-transformed (TPM + 1) values were used for heatmap generation by gplots package.

To analyze the expression patterns of maize and rice *bHLH*s during development, drought and pathogen stresses, the processed expression data sets were downloaded from NCBI SRA and ArrayExpress databases with accession numbers SRP010680, E-GEOD-40070, E-MTAB-4219, E-MTAB-2037 and E-GEOD-65022. The differential expression analysis of maize genes after *Fusarium verticillioides* infection was performed using DEseq2 package. The log2-transformed (FPKM+ 1) and log2-transformed fold change values were used to generate heatmaps.

### Co-expression network construction and GO enrichment analysis

We extended the expression analysis by carrying out a weighted gene co-expression network nalysis (WGCNA) with package WGCNA in R [88]. As it is believed that low expressed and non-changing genes provide limited information in a co-expression network building, 35,702 wheat genes were selected based on their expression values ≥10 TPM in one or more samples and coefficient of variation (CV) ≥ 0.6. And other 563 genes of interest were added into the analysis. To check that there are no outliers, 57 samples were clustered using the function *hclust*. Based on the scale free topology analysis by *pickSoftThreshold* function, the lowest soft-thresholding power value 12 was selected to compute an adjacency matrix (AM). Topological overlap matrix (TOM) was computed from AM, and in turn converted into a dissimilarity TOM. Genes were then clustered using hierarchical clustering and distinct modules were identified using Dynamic Tree Cut approach. Modules whose eigengenes were highly correlated were merged with a mergeCutHeight of 0.25. In order to study the relationships among modules, module similarity was quantified based on the correlation of *module eigengenes* (*MEs*). To select potential biologically interesting modules for downstream analysis, the correlation between modules and tissues type were estimated using *MEs*.

Using BioMart data mining tool supported by Ensembl Plants, Gene Ontology (GO) annotation was obtained to generate a broad overview of groups of genes. GO enrichment analysis was performed for each module using the OmicShare tools (www.omicshare.com/tools). As TFs are crucial in regulating gene expression and triggering multiple aspects of biological process, *bHLH*s were considered as the “guide-gene” to construct subnetworks. The node and edge information of subnetworks was visualized and analyzed using Cytoscape (http://www.cytoscape.org/).

### Plant materials and qPCR validation

Seeds of Nipponbare and a leading wheat variety TAM107 were surface-sterilized in 1% sodium hypochlorite for 20 min, rinsed in distilled water for six times, and soaked in dark overnight at room temperature. The germinated seeds were cultured in water and grew in a growth chamber with 22 °C/18 °C (day/night), 16 h/8 h (light/dark), and 50% humidity. Then 1 week old seedlings were subjected to dehydration stress, replacing water with 20% (m/V) PEG-6000 solution for 1 h, 6 h and 24 h. Maize inbred line B73 were soaked at room temperature and germinated seeds grew in soil in the growth chamber, keeping 90% soil humidity. Then 1 week old seedlings were subjected to drought stress 12 h and 24 h after soil humidity reduced to 50%. Total RNA were isolated from fresh seedling samples, and the quantitative real time PCR (qPCR) reaction was performed on CFX96 qPCR detection system. *UBQ* (rice: LOC_Os06g46770, maize: GRMZM2G409726) and wheat actin (TRIAE_CS42_1AL_TGACv1_001447_AA0030680) were used as internal reference genes to normalize Ct values of each reaction [89–91].

## Additional files

Additional file 1: **Table S1.** List of 1154 bHLHs from Arabidopsis, rice, maize and wheat and their related information. (XLS 509 kb)

Additional file 2: **Table S2.** A table shows 180 new names of wheat *bHLHs* side by side with previous ones. (XLS 73 kb)

Additional file 3: **Table S3.** The distribution of TabHLHs on wheat chromosomes. (XLS 24 kb)

Additional file 4: **Figure S1.** Amino acid sequence alignment of 1154 Arabidopsis, rice, wheat and maize bHLH domains. The red triangles at the bottom indicate the 31 conserved residues. The loop region has been shortened to better visualize the alignment results. (PDF 358 kb)

Additional file 5: **Figure S2.** The unrooted NJ tree of bHLH domains from Arabidopsis, rice, wheat and maize. The blue, red and green rhombuses indicate the *TabHLH* in A, B and D sub-genomes respectively. (PDF 510 kb)

Additional file 6: **Table S4.** Comparison of the number of genes and intron distribution patterns within the bHLH domain in each bHLH subfamily between wheat and other species. (XLS 84 kb)

Additional file 7: **Figure S3.** Phylogenetic relationships and gene structure features of *TabHLHs.* The phylogenetic tree was constructed with MEGA 6.0 based on a multiple alignment of 571 wheat bHLH domain sequences. For better visualization and comparison, all introns were displayed in the same length. (PDF 5746 kb)

Additional file 8: **Figure S4.** Phylogenetic relationships and gene structural features of *ZmbHLHs.* The phylogenetic tree was constructed with MEGA 6.0 based on a multiple alignment of 231 amino acid sequences of maize bHLH domain. For better visualization and comparison, all introns were displayed in the same length. (PDF 1617 kb)

Additional file 9: **Table S5.** Motif with best possible match and the number in each subfamilies and species. (XLS 38 kb)

Additional file 10: **Figure S5.** Architecture of protein conserved motifs. Motifs are graphically represented as colored boxes drawn to scale for bHLH proteins. (PDF 19037 kb)

Additional file 11: **Figure S6.** Arrangement of secondary structure elements in AtMYC3 (5–242). Secondary structure elements overlaid on the sequence alignment of bHLH N-terminal proteins belonging to three subfamilies III(d + e), XIII and IIIf. (PDF 517 kb)

Additional file 12: **Table S6.** The distribution of *cis*-regulatory elements in *TabHLH* promoters. “Y” means that the element is present in the promoter region. (XLS 648 kb)

Additional file 13: **Table S7.** Summary of RNA-seq data generated and mapping statistics. (XLS 35 kb)

Additional file 14: **Table S8.** The TPM values of 209 *TabHLHs* in 15 tissues. (XLS 85 kb)

Additional file 15: **Table S9.** The FPKM values of 188 *ZmbHLHs* in 18 tissues. (XLS 78 kb)

Additional file 16: **Table S10.** The FPKM values of 110 *OsbHLHs* in seven tissues. (XLS 35 kb)

Additional file 17: **Figure S7.** Heatmap showing the transcriptional abundance of *ZmbHLHs* in 18 tissues. (PDF 448 kb)

Additional file 18: **Figure S8.** Heatmap showing log2-transformed (FPKM+1) values for *OsbHLHs* in 7 tissues. (PDF 850 kb)

Additional file 19: **Table S11.** The TPM values of 80 *TabHLHs* under drought stress. (XLS 39 kb)

Additional file 20: **Table S12.** The FPKM values of 78 *ZmbHLHs* under drought stress. (XLS 71 kb)

Additional file 21: **Table S13.** The RPKM values of 59 *OsbHLHs* under drought stress. (XLS 29 kb)

Additional file 22: **Table S14.** List of primers used in this study for qPCR. (XLS 27 kb)

Additional file 23: **Table S15.** The TPM values of 88 *TabHLHs* under stripe rust (S) and powdery mildew (P) stresses. (XLS 41 kb)

Additional file 24: **Table S16.** Convertion of maize AGPv3 gene IDs to maize AGPv4 gene IDs. (XLS 63 kb)

Additional file 25: **Table S17.** The RPKM values for 147 *ZmbHLHs* after *F. verticillioides* infection. (XLS 42 kb)

Additional file 26: **Figure S9.** Analysis of network topology for various soft-thresholding powers. **a.** The scale-free fit index (y-axis) as a function of the soft-thresholding power (x-axis). **b.** The mean connectivity (degree, y-axis) as a function of the soft-thresholding power (x-axis). (PDF 469 kb)

Additional file 27: **Figure S10.** Module-tissue association. Each row corresponds to a module and each column corresponds to a tissue. The top and bottom number in each cell indicate the correlation coefficient between the module and tissue and *p*-value of the test, respectively. (PDF 3815 kb)

Additional file 28: **Table S18.** Summary of the significantly enriched GO terms (q-value < 0.001) of genes in each co-expression module. BP, biological process; MF, molecule function; CC, cellular component. (XLS 279 kb)

Additional file 29: **Table S19.** List of the “guide genes” of nine subnetwork, and their related information. (XLS 37 kb)

Additional file 30: **Table S20.** The expression profiles of some genes from other families discussed in this study. (XLS 181 kb)

Additional file 31: **Figure S11.** Plyhogenetic analysis of 140 TaDEFLs, 7 AtPDF1s and 6 AtPDF2s. The phylogenetic tree was constructed using MEGA by the NJ method with 1000 bootstrap replications, and the bootstrap values greater than 700 were displayed on the branches. (PDF 411 kb)

Additional file 32: **Figure S12.** Phylogenetic relationship and motif compositions of TaJAZs. a. The phylogenetic tree of TaJAZs constructed using MEGA by the NJ method with 100 bootstrap replicates. **b.** Domain distribution of TaJAZs was investigated using the MEME web server. **c.** The consensus sequence of TIFY and Jas motif from TaJAZs. (PDF 414 kb)

## Abbreviations

bHLH: Basic helix-loop-helix
CRE: Cis-acting regulatory
GO: Gene Ontology
hpi: Hours post-inoculation
JA: Jasmonic acid
PIFs: Phytochrome-Interacting factor
SA: Salicylic acid
WGCNA: Weighted gene co-expression network analysis
