# Supplementary material for: Comparative functional genomics analysis of bHLH gene family in rice, maize and wheat
Source: BMC Plant Biol. 2018 Nov 29;18:309. doi: 10.1186/s12870-018-1529-5 (PMC6267037; doi:10.1186/s12870-018-1529-5)
Supplement: Supplementary file 4 — Figure S1. Amino acid sequence alignment of 1154 Arabidopsis, rice, wheat and maize bHLH domains. The red triangles at the bottom indicate the 31 conserved residues. The loop region has been shortened to better visualize the alignment results. (PDF 358 kb) [file 12870_2018_1529_MOESM4_ESM.pdf]

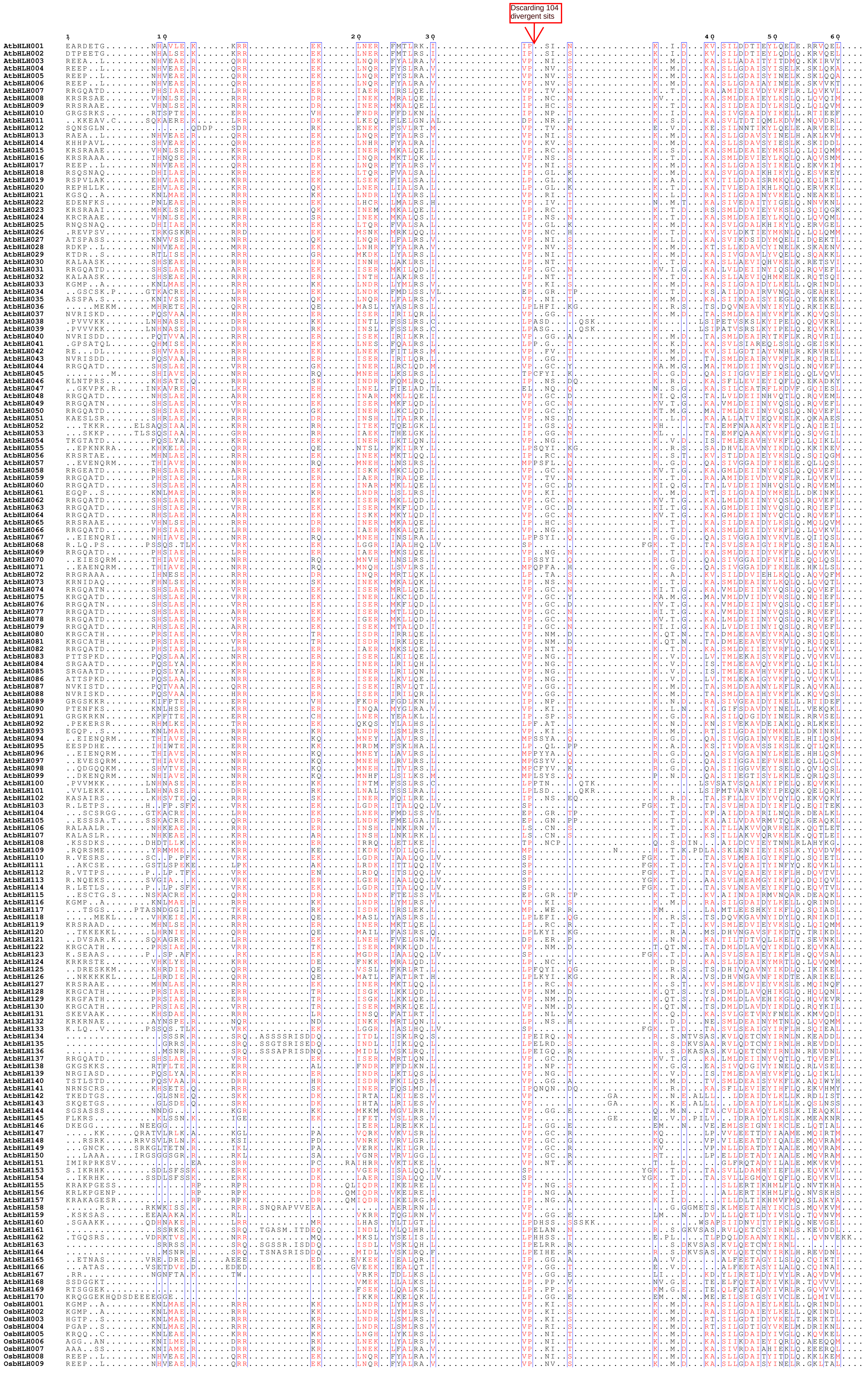
Dscarding 104 divergent sits

```
OsbHLH010  REEP..L........NHVEAE.R......QRR............EK......LNQR..FYALRA.V................VP..KI..S...............K..M.D...KA.SLLSDAIAYIQELE.ARLRGD....
OsbHLH011  GAPP..I........GHVEAE.R......QRR............EK......LNRR..FCELRA.A................VP..TV..S...............R..M.D...KA.SLLADAVDYIAELR.RRVERL....
OsbHLH012  AAGSSIK........NHVMSE.R......RRW............EK......LNEM..FLTLKS.L................VP..SI..D...............K..V.D...KA.SSLAETIAYLKELE.RRVQEL....
OsbHLH013  TQGSSIK........NHVMSE.R......RRR............EK......LNEM..FLILKS.V................VP..SI..H...............R..V.D...KA.SILAETIAYLKELE.KRVEEL....
OsbHLH014  DQGSSIK........NHVMSE.R......RRR............EK......LKEM..FLILKS.V................VP..SI..H...............K..V.D...KA.SILAETIAYLKELE.KRVEEL....
OsbHLH015  TQESGIK........NHVISE.R......RRR............EK......LNEM..FLILKS.I................VP..SI..H...............K..V.D...KA.SILEETIAYLKVLE.KRVKEL....
OsbHLH016  TPGSNIK........SHVMSE.R......RRR............EK......LNEM..FLILKS.L................LP..SV..R...............K..V.D...KA.SILAETITYLKVLE.KRVKEL....
OsbHLH018  RPASQNQ........EHILAE.R......KRR............EK......LSQR..FIALSK.I................VP..GL..K...............K..M.D...KA.SVLGDAIKYVKQLQ.DQVKGL....
OsbHLH019  RPPANAQ........EHVIAE.R......KRR............EK......LQQQ..FVALAT.I................VP..GL..K...............K..T.D...KI.SLLGSTIDYVKQLE.EKVKAL....
OsbHLH020  SAAPYAQ........EHIIAE.R......KRR............EK......INQR..FIELST.V................IP..GL..K...............K..M.D...KA.TILSDAVRYVKEMQ.EKLSEL....
OsbHLH021  AAPGYVQ........DHIIAE.R......RRR............EK......INQR..FIELST.V................IP..GL..K...............K..M.D...KA.TILGDAVKYVKELQ.EKVKTL....
OsbHLH022  ASVAVQL........EHVVAE.R......KRR............EK......INQR..FMELSA.V................IP..KL..K...............K..M.D...KA.TILSDAASYIRELQ.EKLKAL....
OsbHLH023  RATSSMQ........EHVIAE.R......KRR............EK......MHQQ..FTTLAS.I................VP..EI..T...............K..T.D...KV.SVLGSTIEYVHHLR.ERVKIL....
OsbHLH024  .ATNSSQL.......YHMMSE.R......KRR............EK......LNDS..FHTLRS.L................LPP.C...S...............K..K.D...KT.TVLINAAKYLKSLE.TEITEL....
OsbHLH025  .PPSGNQL.......QHMISE.R......KRR............EK......LNDS..FLALKA.V................LPP.G...S...............K..K.D...KT.SILIRAREYVKSLE.SKLSEL....
OsbHLH026  .PPSDNQL.......QHMISE.R......KRR............EK......LNDS..FVALKA.V................LPT.G...S...............K..K.D...KA.SILIRAREHIKSLE.SKLSEL....
OsbHLH027  .PSGNNQL.......QHTMSE.R......KRR............EK......LNDS..FVALKA.V................LPP.G...S...............K..K.D...KT.SILIRAREYVKSLE.SKLSEL....
OsbHLH028  ....ENQQL....QLQHVLSE.R......KRR............EK......LNDS..FKALRD.V................LPP.A...T...............K..K.D...KA.SVLMRAKDYVNVLK.ARIAEL....
OsbHLH029  .PPTSSQL.......HHMISE.R......RRR............ER......LNES..FEHLRG.L................LPP.G...S...............K..K.D...KA.TVLAKTLEYMNLLI.AQISEL....
OsbHLH030  .PPSSSQL.......HHMISE.R......RRR............ER......INDS..FQTLRA.LL..............PLPP.D...S...............K..K.D...KA.AILASTTEYMDKLI.SQVSEL....
OsbHLH031  MPNTPRS........KHSATE.Q......RRR............SK......INDR..FQLLRD.L................LP..HN..DQ..............K..R.D...KA.SFLLEVIEYIRFLQ.EKVQKY....
OsbHLH032  RPSSPRS........KHSATE.Q......RRR............SK......INDR..FQILRE.L................LP..HS..DQ..............K..R.D...KA.TFLLEVIEYIRFLQ.EKVQKF....
OsbHLH034  GPSTPRS........KHSATE.Q......RRR............TK......INDR..LEILRE.L................LP..HT..DQ..............K..R.D...KA.SFLSEVIEYIRFLQ.EKVQKY....
OsbHLH035  EAR.ALK........IHSEAE.R......RRR............ER......INAH..LTTLRR.M................IP..DT..K...............Q..M.D...KA.TLLARVVDQVKDLK.RKASEI....
OsbHLH036  QAT.AMT........IHSEAE.R......RRR............ER......INAH..LATLRR.I................LP..DA..K...............Q..M.D...KA.TLLASVVNQVKHLK.TRATEA....
OsbHLH037  KAAMALK........SHSEAE.R......RRR............ER......INAH..LATLRT.M................VP..CT..D...............K..M.D...KA.ALLAEVVGHVKKLK.SAAARV....
OsbHLH038  KALAASR........SHSEAE.R......RRR............QR......INGH..LARLRS.L................LP..NT..T...............K..T.D...KA.SLLAEVIEHVKELK.RQTSAM....
OsbHLH039  KALAASR........SHSEAE.R......RRR............QR......INGH..LARLRS.L................LP..NT..T...............K..T.D...KA.SLLAEVIEHVKELK.RQTTAI....
OsbHLH040  KALAASK........SHSEAE.R......RRR............ER......INAH..LARLRS.L................LP..NT..T...............K..T.D...KA.SLLAEVIQHVKELK.RQTSEI....
OsbHLH041  RALAASR........NHREAE.K......RRR............ER......IKSH..LDRLRA.V................LA..CD..P...............K..I.D...KA.SLLAKAVERVRDLK.QRMAGI....
OsbHLH042  GGSPAVR........SHSEAE.R......KRR............QR......INAH..LATLRT.L................VP..SA..S...............R..M.D...KA.ALLGEVVRHVRELR.CRADDA....
OsbHLH043  RWTRARR........SHSEAE.R......KRR............ER......INAH..LDTLRG.L................VP..SA..S...............R..M.D...KA.ALLGEVVRYVRKLR.SEAAGS....
OsbHLH044  ..EIECQRM......THIAVE.R......NRR............RQ......MNEY..LAVLRS.L................MPASYS..Q...............R..G.D...QA.SIVGGAINYVKELE.QLLQSL....
OsbHLH045  ..EIESQRM......THIAVE.R......NRR............RQ......MNEY..LAVLRS.L................MPPSYA..Q...............R..G.D...QA.SIVGGAINYVRELE.QLLQTL....
OsbHLH046  ..EVESQRM......THIAVE.R......NRR............KQ......MNEY..LAVLRS.L................MPPSYA..Q...............R..G.D...QA.SIVGGAINFVKELE.QLLQSL....
OsbHLH047  ..EVESQRM......THIAVE.R......NRR............KQ......MNEY..LAVLRS.L................MPASYV..Q...............R..G.D...QA.SIIGGAINYVKEME.QLLQSL....
OsbHLH048  ..EAESQRR......NHIAVE.R......NRR............RQ......MNEY..LAVLRS.L................MPPSYA..Q...............R..G.D...QA.SIVAGAINFVKELE.QLLQSL....
OsbHLH049  ..ETETQRM......THIAVE.R......NRR............RQ......MNEY..LAILRS.L................MPEPYV..Q...............R..G.D...QA.SIVGGAIEFVKELE.QQLQSL....
OsbHLH050  ..DAESQRM......THIAVE.R......NRR............RQ......MNEY..LAVLRS.L................MPESYV..H...............R..G.D...QA.SIVGGAIDFVKELE.QLLQSL....
OsbHLH051  ..EVESQRM......THIAVE.R......NRR............RQ......MNEY..LRVLRS.L................MPGSYV..Q...............R..G.D...QA.SIIGGAIEFIRELE.QLIQCL....
OsbHLH052  ..EAENQRM......THIAVE.R......NRR............RL......MNDH..LASLRS.L................IPSNYI..P...............R..G.D...QA.TVVGGAIDYVKQLE.QQLVAL....
OsbHLH053  ..GDGAAKM......SHITVE.R......NRR............KQ......MNEH..LAVLRS.L................MPCFYV..K...............R..G.D...QA.SIIGGVVDYIKELQ.QVLRSL....
OsbHLH054  ..AATTPKT......AHIAVE.R......NRR............KQ......MNEN..LAVLRS.L................MPCFYV..K...............R..G.D...QA.SIIGGVVDYIKELQ.QVLHSL....
OsbHLH055  ........M......SHIAVE.R......NRR............RQ......MNDH..LKVLRS.L................TPAFYI..K...............R..G.D...QA.SIIGGAIDFIKELQ.TLLQSL....
OsbHLH056  .SGSHRK.......LSHNAYE.R......DRR............KQ......LNEL..YSSLRA.L................LPDAD.....HTK...........K.......LSIPTTVSRVLKYIPELQ.KQVENL....
OsbHLH057  ..ESGTR.P.....SSKACRE.K......VRR............DK......LNER..FLELGA.VL...............EP..GK..TP..............K..M.D...KS.SILNDAIRVMAELR.SEAQKL....
OsbHLH058  ..ESSAP.P.....GTKACRE.K......LRR............DR......LNER..FNELSA.IL...............EP..GK..PP..............R..A.D...KV.SILSDAARLLSQLR.AEAQKL....
OsbHLH059  ...SSSSGP.....KSKACRE.K......IRR............DR......LNDR..FLELSS.VI...............NP..DK..QA..............K..L.D...KA.NILSDAARLLAELR.GEAEKL....
OsbHLH060  ..GSCGR.P.....TSKASRE.K......IRR............DK......MNDR..FLELGT.TL...............EP..GK..PV..............K..S.D...KA.AILSDATRMVIQLR.AEAKQL....
OsbHLH061  ..EKAPK.K.....THKAERE.K......LKR............DQ......LNDL..FVELSS.ML...............DP..ER..Q...............N..S.G...KA.TVLGDAARVLRDLV.SQVESL....
OsbHLH062  ..KKAPK.R.....IHKSERE.K......LKR............DK......QNDL..FNELGN.LL...............EP..DR..Q...............N..N.G...KA.CVLGETTRILKDLL.SQVESL....
OsbHLH063  ..KKVPR.K.....VHKSERE.K......LKR............GH......LNDL..FGELGN.ML...............EA..DR..Q...............S..N.G...KA.CILTDTTRILRDLL.SQVKSL....
OsbHLH064  ..SIATR.K.....VQKADRE.K......MRR............DR......LNEQ..FQELGS.TL...............DP..DR..P...............R..N.D...KA.TILSDAIQMLKDLT.SQVNKL....
OsbHLH065  RNT.SPR.......D..APVSPK......EKK............DK......IGER..VAALQQ.LV...............SP.....................FGK..T.D...TA.SVLQEASGYIKFLH.QQLEVL....
OsbHLH066  P.AAATT.......TNKRP...R......VRR............ER......LGER..IIALQQ.LV...............SP.....................FGK..S.D...TA.SVLHEALGYIRFLH.DQVQVL....
OsbHLH067  R.IEAPS.......P..MP.TFK......VRK............EK......LGDR..ITALQQ.LV...............SP.....................FGK..T.D...TA.SVLHEAIEYIKFLH.DQVASL....
OsbHLH068  R.LETPS.......P..LP.TFK......VRK............EK......LGDR..ITALQQ.LV...............SP.....................FGK..T.D...TA.SVLHETIEYIKFLH.DQVGAL....
OsbHLH069  S.CSSNA.......NTLLA...K......VRK............EK......LGDR..ITALQQ.LV...............SP.....................FGK..T.D...TA.SVLHEAIEYIKFLH.EQVASL....
OsbHLH070  ..LSQMV.......IDFLPIKFH......VRK............EK......LGDR..VTALQQ.LV...............SP.....................FGK..T.D...TA.SVLHETIEYIKFLH.DQVGAL....
OsbHLH071  R.LESHS.......SSMLP.SFK......VRK............EK......LGDR..IAALQQ.LV...............SP.....................FGK..T.D...TA.SVLMEAIGYIKFLQ.DQVETL....
OsbHLH072  R.TQ.EP.......SPAQA.TVK......VRK............EK......LGDR..ITALHQ.LV...............SP.....................FGK..T.D...TA.SVLLEAIGYIRFLH.GQIEAL....
OsbHLH073  R.VQ.AS.......SSAQS.TLK......VRK............ER......LGDR..ITALHQ.IV...............SP.....................FGK..T.D...TA.SVLQETIGYIRFLL.SQIEAL....
OsbHLH074  R.VQ.AS.......SSAQS.TLK......VRK............ER......LGDR..ITALHQ.IV...............SP.....................FGK..T.D...TA.SVLQETIGYIRFLL.GQIEAL....
OsbHLH075  KSETSHS.......TSSPK..HQ......SPK............VK......LGEK..ITALQQ.IV...............SP.....................FGK..T.D...TA.SVLLETITYIKFLH.EQIQLF....
OsbHLH076  KQEAS.K.......ASPPK..QP......VPK............VK......LGEK..ITALQQ.IV...............SP.....................FGK..T.D...TA.SVLFETIKYIKFLH.EQVQLL....
OsbHLH077  HEATSPT........SSLK..SQ......VPK............VK......LGDK..ITALQQ.IV...............SP.....................FGK..T.D...TA.SVLYEAINYIKWLH.EQVQLL....
OsbHLH078  RNQTTKA.......TCK......      KRS............QK......LGDR..ITALQQ.IV...............SP.....................YGK..T.D...TA.SVLHEAAACIRQLH.QQIQIL....
OsbHLH079  RRGQATD........SHSLAE.R......VRR............ER......ISER..MRYLQE.L................VP..GC..N..............KV.T.G...KA.GMLDEIINYVQSLQ.KQVEFL....
OsbHLH080  RRGQATD........SHSLAE.R......VRR............ER......ISER..MRMLQA.L................VP..GC..D..............KV.T.G...KA.LILDEIINYVQSLQ.NQVEFL....
OsbHLH081  RRGQATD........SHSLAE.R......VRR............ER......ISER..MRMLQA.L................VP..GC..D..............KV.T.G...KA.LILDEIINYVQSLQ.NQVEFL....
OsbHLH083  RRGQATD........SHSLAE.R......VRR............EK......ISER..MKMLQS.L................VP..GC..D..............KV.T.G...KA.LMLDEIISYVQSLQ.NQVEFL....
OsbHLH084  KRGQATN........SHSLAE.R......LRR............KK......ISER..MKLLQD.L................VP..GC..S..............KI.T.G...KA.VMLDEIINYVQSLQ.RQVEFL....
OsbHLH085  RRGQATN........SHSLAE.R......LRR............EK......ISER..MKLLQD.L................VP..GC..S..............KV.T.G...KA.VMLDEIINYVQSLQ.RQVEFL....
OsbHLH086  RRGQATN........SHSLAE.R......VRR............EK......ISER..MKYLQD.L................VP..GC..S..............KV.T.G...KA.VMLDEIINYVQSLQ.RQVEFL....
OsbHLH087  RKGQATN........NHSLAE.R......LRR............EK......ISER..MKLLQD.L................VP..GC..S..............KV.T.G...KA.LMLDEIINYVQSLQ.RQVEFL....
OsbHLH088  KRGQATN........SHSLAE.R......FRR............EK......INER..MKLLQD.L................VP..GC..N..............KI.T.G...KA.MMLDEIINYVQSLQ.RQVEFL....
OsbHLH089  RRGQATD........SHSLAE.R......ARR............EK......ISER..MKILQD.L................VP..GC..N..............KV.I.G...KA.SVLDEIINYIQALQ.RQVEFL....
OsbHLH090  RRGEATD........SHSLAE.R......VRR............EK......ISQR..MKLLQD.L................VP..GC..N..............KV.V.G...KA.VMLDEIINYVQSLQ.RQVEFL....
OsbHLH091  RRGQATD........SHSLAE.R......VRR............ER......ISQR..MKVLQD.L................VP..GC..N..............KV.I.G...KA.LMLDEIINYVQSLQ.RQVEFL....
OsbHLH092  RRGQATD........SHSLAE.R......VRR............EK......ISQR..MKVLQD.L................VP..GC..N..............KV.V.G...KA.LMLDEIINYVQSLQ.QQVEFL....
OsbHLH093  RRGQATD........SHSLAE.R......VRR............ER......ISER..MKLLQS.L................VP..GC..N..............KI.T.G...KA.LMLDEIINYVQSLQ.RQVEFL....
OsbHLH094  RRGQATD........SHSLAE.R......ARR............EK......ISER..MKILQD.L................VP..GC..N..............KV.I.G...KA.SVLDEIINYIQSLQ.HQVEFL....
OsbHLH095  ...............................................................MELLKE.L................VP..GC..S..............KV.S.G...TA.LVLDEIINHVQSLQ.RQVEYL....
OsbHLH096  RRGQATD........PHSIAE.R......LRR............EK......ISER..MKNLQV.L................VP..NS..N...............K..A.D...KA.SMLDEIIDYVKFLQ.LQVKVL....
OsbHLH097  RRGQATD........PHSIAE.R......LRR............EK......ISDR..MKDLQE.L................VP..NS..N...............K..T.N...KA.SMLDEIIDYVKFLQ.LQVKVL....
OsbHLH098  RRGQATD........PHSIAE.R......LRR............ER......IAER..MRALQE.L................VP..NT..N...............K..T.D...RA.AMLDEILDYVKFLR.LQVKVL....
OsbHLH099  ...............................................................MRALQD.L................VP..NT..N...............K..T.D...RA.AMLDEILDYVKFLR.LQVKVL....
OsbHLH100  RRGQATD........PHSIAE.R......LRR............ER......IAER..MKALQE.L................VP..NA..N..............KLQT.D...KA.SMLDEIIDYVKFLQ.LQVKAS....
OsbHLH101  RRSRSAE........FHNFSE.R......RRR............DR......INEK..LKALQE.L................LP..NC..T...............K..T.D...KV.SMLDEAIDYLKSLQ.LQLQML....
OsbHLH102  KRRRAAE........VHNLSE.R......RRR............DR......INEK..MKALQE.L................IP..HC..N...............K..T.D...KA.SMLDEAIEYLKSLQ.LQLQMM....
OsbHLH103  RRSRAAE........VHNLSE.R......RRR............DR......INEK..MRALQE.L................IP..HC..N...............K..T.D...KA.SMLDEAIEYLKSLQ.LQLQMM....
OsbHLH104  RRTRAAE........VHNLSE.R......RRR............DR......INEK..LRALQE.L................VP..HC..N...............K..T.D...KA.SILDEAIEYLKSLQ.MQVQIM....
OsbHLH106  KRSRTAE........VHNLSE.R......RRR............DR......INEK..MRALQE.L................IP..NC..N...............KI...D...KA.SMLEEAIEYLKTLQ.LQVQMM....
OsbHLH107  KRSRAAE........VHNLSE.K......RRR............SR......INEK..MKALQS.L................IP..NS..S...............K..T.D...KA.SMLDDAIEYLKQLQ.LQVQML....
OsbHLH108  KRSRAAE........VHNLSE.K......RRR............SK......INEK..MKALQS.L................IP..NS..N...............K..T.D...KA.SMLDEAIEYLKQLQ.LQVQML....
OsbHLH109  KRGCATH........PRSIAE.R......ERR............TR......ISKR..LKKLQD.L................VP..NM..D...............K.QT.N...TS.DMLDIAVTYIKELQ.GQVEKL....
OsbHLH110  KRGCATH........PRSIAE.R......ERR............TR......ISEK..LRKLQE.L................VP..NM..D...............K.QT.S...TA.DMLDLAVEHIKGLQ.SQLQAL....
OsbHLH111  KRGCATH........PRSIAE.R......ERR............TR......ISEK..LRKLQA.L................VP..NM..D...............K.QT.S...TS.DMLDLAVDHIKGLQ.SQLQTL....
OsbHLH112  KRGCATH........PRSIAE.R......VRR............TR......ISER..IRKLQE.L................VP..NM..D...............K.QT.N...TA.DMLDLAVDYIKDLQ.KQVKGL....
OsbHLH113  KRSRAAA........IHNESE.R......KRR............DR......INQK..MKTLQK.L................VP..NS..S...............K..T.D...KA.SMLDEVIDYLKQLQ.AQVQVM....
OsbHLH114  ...PFSP........PFFIAS.MP...CCLRR............ER......IAER..MKALQE.L................VP..NA..N...............K..T.D...KA.SMLDEIIDYVKFLQ.LQVKVL....
OsbHLH115  SGGAGQP........PFG.............QLRR..........ER......IAER..MKSLQE.L................VP..NA..N...............K..T.D...KA.SMLDEIIDYVKFLQ.LQVKVL....
OsbHLH116  .KSGDSI........DELFTKFH......RRR............FK......ITER..FRTLQR.L................VP..GC..D...............K..S.N...QA.STLDQTIQYMKSLQ.HQLKAM....
OsbHLH117  NVRISSD........PQTVAA.R......QRR............ER......ISER..LRVLQK.L................VP..GG..A...............K..M.D...TA.SMLDEAANYLRFLK.SQIREL....
OsbHLH118  NVRISSD........PQTVAA.R......LRR............ER......VSDR..LRVLQK.L................VP..GG..N...............K..M.D...TA.SMLDEAASYLKFLK.SQVQKL....
OsbHLH119  NVRISSD........PQTVAA.R......LRR............ER......VSER..LRVLQR.L................VP..GG..S...............K..M.D...TA.TMLDEAASYLKFLK.SQLEAL....
OsbHLH120  NVRISKD........PQSVAA.R......LRR............ER......ISER..IRILQR.L................VP..GG..T...............K..M.D...TA.SMLDEAIHYVKFLK.SQVQSL....
OsbHLH121  NVRISTD........PQSVAA.R......MRR............ER......ISER..IRILQR.L................VP..GG..T...............K..M.D...TA.SMLDEAIHYVKFLK.TQVQSL....
OsbHLH122  NARVSSE........PQSVAA.R......LRR............ER......VSQR..MRALQR.L................VP..GG..A...............R..L.D...TA.SMLEEAIRYVKFLK.GHVQSL....
OsbHLH123  GAKLSTD........PQSVAA.R......ERR............HR......ISDR..FRVLRS.L................VP..GG..S...............K..M.D...TV.SMLEQAIHYVKFLK.AQVTLH....
OsbHLH124  NVRISDD........PQSVAA.R......HRR............ER......ISER..IRILQR.L................VP..GG..T...............K..M.D...TA.SMLDEAIRYIKFLK.RQVQEL....
OsbHLH125  .PSPNKEQ.......PQSAAA.K......VRR............ER......ISER..LKVLQD.L................VP..NG..T...............K..V.D...LV.TMLEKAINYVKFLQ.LQVKVL....
OsbHLH126  PTTPTKD........PQSLAA.K......NRR............ER......ISER..LRILQE.L................VP..NG..T...............K..V.D...LV.TMLEKAISYVKFLQ.LQVKVL....
OsbHLH127  QRANNKE........TQSSAA.K......SRR............ER......ISER..LRALQE.L................VP..SG..G...............K..V.D...MV.TMLDRAISYVKFMQ.MQLRVL....
OsbHLH128  NRGAATD........PQSLYA.R......KRR............ER......INER..LRILQN.L................VP..NG..T...............K..V.D...IS.TMLEEAVQYVKFLQ.LQIKLL....
OsbHLH129  GRGAATD........PQSLYA.R......KRR............ER......INER..LKILQN.L................IP..NG..T...............K..V.D...IS.TMLEEAVHYVKFLQ.LQIKLL....
OsbHLH130  GRGAATD........PQSLYA.R......KRR............ER......INER..LKTLQN.L................VP..NG..T...............K..V.D...IS.TMLEEAVHYVKFLQ.LQIKLL....
OsbHLH131  GHRSATD........PQSLYA.R......KRR............ER......INER..LKILQN.L................VP..NG..T...............K..V.D...IS.TMLEEAMHYVKFLQ.LQIKLL....
OsbHLH132  HKQCSKD........TQSLYA.K......RRR............ER......INER..LRILQQ.L................VP..NG..T...............K..V.D...IS.TMLEEAVQYVKFLQ.LQIKLL....
OsbHLH133  HSVSAES........SQSYYA.K......NRR............QR......INER..LRILQE.L................IP..NG..T...............K..V.D...IS.TMLEEAIQYVKFLH.LQIKLL....
OsbHLH134  RRRSATI........AQSLYA.K......RRR............ER......INGR..LRILQK.L................VP..NG..T...............K..V.D...IS.TMLEEAVHYVKFLQ.LQIKVE....
OsbHLH135  RNPGYAE........THGLTE.K......RRR............SR......INEK..FKMLQR.L................VP..GC..D...............K..C.S...QS.STLDRTIHYMKSLQ.QQLQAM....
OsbHLH136  RHRHAAG........AHNLTE.K......RRR............FK......ITER..FRTLQR.L................VP..GC..DN..............K..S.N...QA.STLDQTIQYMKSLQ.HQLEAT....
OsbHLH137  ......E........DQVPTD.EGICVMQKRR............CK......INEN..LKTLQQ.L................VP..VC..D...............K.SN.N...QA.STLDKTIRYMKSLQ.QHAQAM....
OsbHLH138  ..GEEAE.......GDDGPDDRK......RRR............ER......IQET..VAALRK.I................VPG.GI..A...............K........DATAVLDEAICYLKYLK.LKVKTL....
OsbHLH139  SSSY............GGGQARPG....RKK............ER......MKKM..VRTLKG.I................IP..GG..N...............Q..M.D...TP.AVLDEAVRYLKSLK.VEVKKL....
OsbHLH140  GGGRKPE........A.GGGE........RKK............ER......MKKM..MRTLKG.I................IP..GG..D...............R..M.D...TP.AVLDEAVRYLKSLK.VEVKKL....
OsbHLH141  GKGKGKA........N.FATE.R......ERR............EQ......LNVK..FRTLRM.L................FP..NP..T...............K..N.D...RA.SIVGDAIEYIDELN.RTVKEL....
OsbHLH142  ANG.VEK........K....E.K......QRR............LR......LTEK..YNALML.L................IP..NR..T...............K..E.D...RA.TVISDAIEYIQELG.RTVEEL....
OsbHLH143  KRGCATH........PRSIAE.R......VRR............TR......ISER..IRKLQE.L................VP..NM..E...............K.QT.N...TA.DMLDLAVDYIKELQ.KQV..LND..
OsbHLH144  AMDMEHA........LHIWTE.R......ERR............KK......MKNM..FSTLHG.L................LP..KI..PG..............K..T.D...KA.SIVGEAIGYIKTLE.DVVQKL....
OsbHLH146  GGRSRER........MHIFAE.R......ERR............RK......IKNM..FTDLRD.L................VP..SL..TN..............K..A.D...KA.TIVGEAISFIRSLE.ETVADL....
OsbHLH147  ..GGGNR......ACMFAVRE.R......ERR............RR......MNDM..FAGIRR.L................VP..NLP.E...............K....E......EVRMEAQK..QELQ.RERDRL....
OsbHLH148  .VESSRGF.......RHMMRE.R......QRR............EK......LSQS..YADLYA.M................V...SS..RS..............K..G.D...KN.SIVQSAAIYIHELK.VARDQL....
OsbHLH149  KRSRPGESA.............RP.....RPK............DR....QMIQDR..IKELRE.I................VP..NS..A...............K..I.D......TLLEKTIKHMLFLQ.NVAKHA....
OsbHLH150  KRSRPGESP.............KP.....RPK............DR....QLIQDR..IKELRE.L................VP..NG..A...............K..I.D......ALLEKTIKHMVFLQ.SVTKHA....
OsbHLH151  KRSRPGESP.............KP.....RPK............DR....QLIQDR..IKELRE.M................VP..NG..A...............K..I.D......ALLEKTVKHMLFLQ.SVTKHA....
OsbHLH152  RRTRAAE........VHNLSE.R......RRR............DR......INEK..MRALQE.L................IP..HC..N...............K..T.D...KA.SILDEAIEYLKSLQ.MQVQIM....
OsbHLH153  .................MSS.........RRGGGGGGGRITDEE......INEL..ISKLQA.L................LPESSR..S...............R..A.SRSSAS.KLLKETCSYIKSLH.REVDDL....
OsbHLH154  ................GS.SV....PSSS..SSSRTSISEDQ......IAEL..LSKLQA.L................LPESQA..R...............N..A.HRGSAA.RVLQETCSYIRSLH.QEVDNL....
OsbHLH155  RVEGMPS........KNLMAE.R......RRR............KR......LNDR..LSMLRS.V................VP..KI..S...............KV.L.S...ES.FCVAIGIASAREMR.LDDV......
OsbHLH156  RRDR..S........KTIVSE.R......KRR............VR......MKEK..LYELRA.L................VP..NI..T...............K..M.D...KA.SIIADAVVYVKDLQ.AHARKL....
OsbHLH157  .RRTPLM.......SSSSEVA.R......RL.....................VKKR..DKVLRR.M................IP..GG..E...............LI...D...EI.SLLHEAMDYVVHLR.AQVDVL....
OsbHLH158  .RSGAG.........GGGEMA.R......RL...................QVRRR..AMALRK.V................IP..GG..G...............DA...D...EA.ALLREAMDYVVHLR.AQVDVL....
OsbHLH159  KNSGSNK......ERR..RM.........RR...............CVE..VRRK..MEALRR.L................VP..GG..G...............G.........E.ELLFRAADYIARLQ.VQVKVM....
OsbHLH160  .................MSG.K......DQK............KA......TTAL..EEKLEL.L................RDV.....T...............K..S.SAANET.SILVDASKYIKELK.DKVSQE....
OsbHLH161  .................MSG.K......DQK............KA.......AAL..EEKLEL.L................RSV.....T...............K..S.SAANET.SILVDASKYIKELK.DKVSQE....
OsbHLH162  .................MVS.R......EQ.............K.......RASL..HEKLQI.L................RTL.....T...............H..S.HAVNKM.SIISDASTYIKDLK.QKIAAL....
OsbHLH163  .................MMS.R......ERK............K.......AAAL..HEKLQI.L................RSI.....T...............H..S.HALSNT.SIITDASEYIKELK.QKVVRL....
OsbHLH164  MGKEFKS........KNLEAE.R......RRR............GR......LNGN..IFALRA.V................VP..KI..T...............K..M.S...KE.ATLSDAIEHIKNLQ.NEVLEL....
OsbHLH165  AGSCSIK........NHVMSE.R......RRR............EK......LNEM..FLILKS.L................VP..SI..D...............K..V.D...KA.SILSETIAYLKELE.RRVQEL....
OsbHLH166  ................MMSS.R......ERK............K.......AAAL..QEKLQI.L................RSI.....T...............H..S.HALSNT.SILMDASKYIKELK.QKVVRL....
OsbHLH167  ..PT..V........SHVEAE.R......QRR............EK......LNRR..FCDLRA.A................VP..TV..S...............R..M.D...KA.SLLADAAAYIAELR.ARVARL....
OsbHLH168  KAYGGGG.......GGGGGVA.R......AM.....................VRKR..ASVLKE.I................VP..GG..K...............AL...D...MC.ALLGETLDYAVSLK.AQVDVM....
OsbHLH169  .QGKAME.......GEAEQEE.E......EEE............VM......VDEK..VAVLRQ.L................VP...G..G...............E.AM.....AVERLLDETADYIAALR.AQVGVM....
OsbHLH170  .................MSS.........RR...SSRGSISEEE......INEL..ISKLQS.L................LPNS.R..R...............R..S.SQASTT.KLLKETCNYIKSLH.REVDDL....
OsbHLH171  AASGGGG.........ALAA.R......RM.....................VKGR..LRLLRR.L................VP..GG..E...............AL...R...GF.SLLSETLDYVVCLK.TQVELM....
OsbHLH172  .................MSS.........RR...SSRSSVSEEE......INEL..ISKLQS.L................LPSS.R..R...............R..A.NQASTT.KLLKETCSYIKSLH.REVDDL....
OsbHLH173  ...............MSRRS.R......SRA..SSAAR.ITDEQ......IGDL..VSKLQA.L................LPEARL..R..................N.DRVPSA.RVLQETCSYIRSLH.REVDDL....
OsbHLH174  .................MSG.........RR...ASGRITDDE......INEL..ISKLQS.L................LPESSR..R...............R..ATSRSPAT.KLLKEMCSYIKSLH.REVDDL....
OsbHLH175  .....AG.......ETSAAA.R......AV.............PPPPRQQGEPPR..AEALRR.L................VP..GG..A...............GM...E...YS.SLLEETADYLRSLR.AQVQLM....
OsbHLH176  SRH..PS.....SSSGKG...........PKA............PA......LARK..AKVLGR.L................VP..GC..R...............KL.........FP.ALLAEASDYIAALE.MQVRAM....
OsbHLH177  ..SGGRR.....RRIRPAAA.R......RS...............GVA..LRRK..VRELRR.L................VP..GG..E...............GA.........AR.SLLVRTADYIVRLK.ARVELL....
OsbHLH178  P....PP.....AARPAP...........RSR............PP......LASK..AKVLGR.L................VP..GC..R...............KL.........FP.ALLAEASDYIAALE.MQVRAM....
OsbHLH179  ..SSGSD.......PNKKKSEHQ......SPK............VN......LGEK..IMALQQ.IM...............SP.....................FGK..T.D...TA.SVLLETITYIKFLHGTEVSI....
OsbHLH180  SSSQSSV........FHGSMDT.......VTH............ER......MRNM..VTVLRG.I................IP..GG..D...............Q..L.D...TA.SVIEEAVRYLKFLK.MEAKKL....
OsbHLH181  ...HHHH.....NQAVQSAAA.R......ERR............RR......ISSK..TAELSR.L................IP..GA..A...............RM...S...TA.EMLQAAARHVRLLQ.AQVGML....
OsbHLH182  ...KAAA.....AASSAGEPP.M......LKE............RK......VKER..LRVLGR.L................VP..GC..R...............KL.........AP.ALLEETADYVAALE.MQVKAM....
OsbHLH183  .EAASGR.......PPKAGSE.R......ARR............QT......MSRL..YDELGA.L................LP..NL..P.............PR..A.S...TT.RIVEEAIACVGELR.ARTAEL....
OsbHLH184  .EEELR.......RPPPPRR.R......RQQ............PT......MAAL..YAELAS.L................LP..GL..............RSR..A.S...RE.EIVEAAAKQVKVLE.DTVAVL....
OsbHLH185  SGGSAR.F.......RHIMRE.R......LRR............ER......LSQG..YADLQA.I................LPT.GA..SSSKVLVKSLIRNYMS..G.G...KN.TIVAAAANYIRELE.GRKGWL....
OsbHLH186  ................RRS.RT......SSR..LAAAPPPTDEQ......MAEL..ISKLQA.V................LPTR............................EANAKASSA.EVLQEACRYIRRLH.READAL....
OsbHLH187  .TAAAAA.......AERKEME.R......RRR............QD......MKGL..CVKLAS.L................IPK.....E...............HC...ASRTQL.GSLDEAAAYIKKLK.ERVDEL....
OsbHLH188  GRCRKTGLH..............RP......RPR............DR....QLIQDR..MMELRQ.L................VP..NT..S...............KV.I.D......SLLDKTIAHMQFLQ.CVSEKA....
TabHLH001  KSRAAMD........SQSNYA.K......RRR............HK......INER..LRVLQH.L................IP..NG..T...............K..V.D...IS.TMLEEAVQYVKFLH.LQIKLL....
TabHLH002  KSRASMD........SQSNYA.K......RRR............HK......INER..LRVLQH.L................IP..NG..T...............K..V.D...IS.TMLEEAVQYVKFLH.LQIKLL....
TabHLH003  MSRAGLD........SQSNYA.K......RRR............HK......INER..LRVLQH.L................IP..NG..T...............K..V.D...IS.TMLEEAVHYVKFLH.LQIKLL....
TabHLH004  SRGAATD........PQSLYA.R......KRR............ER......INER..LKTLQN.L................VP..NG..T...............K..V.D...IS.TMLEEAVEYVKFMQ.LQIKLL....
```

```
TabHLH005 SRGAATD........PQSLYA.R......KRR..........ER......INER..LKTLQN.L...............VP..NG..T..............K..V.D...IS.TMLEEAVEYVKFMQ.LQIKLL....
TabHLH006 SRGAATD........PQSLYA.R......KRR..........ER......INER..LKTLQN.L...............VP..NG..T..............K..V.D...IS.TMLEEAVEYVKFMQ.LQIKLL....
TabHLH007 GRGAATD........PQSLYA.R......KRR..........EK......INER..LKVLQN.L...............IP..NG..T..............K..V.D...IS.TMLEEAVHYIKFMQ.LQIKLL....
TabHLH008 GRGAATD........PQSLYA.R......KRR..........EK......INER..LKVLQN.L...............IP..NG..T..............K..V.D...IS.TMLEEAVHYIKFMQ.LQIKLL....
TabHLH009 GRGAATD........PQSLYA.R......KRR..........QK......INER..LKVLQK.L...............IP..NG..T..............K..V.D...IS.TMLEEAVHYIKFMQ.LQIKLL....
TabHLH010 GRGAATD........PQSLYA.R......KRR..........ER......INER..LKTLQT.L...............VP..NG..T..............K..V.D...MS.TMLEEAVQYVKFLQ.LQIKVL....
TabHLH011 GRGAATD........PQSLYA.R......KRR..........ER......INER..LKTLQT.L...............VP..NG..T..............K..V.D...MS.TMLEEAVQYVKFLQ.LQIKVL....
TabHLH012 GRGAATD........PQSLYA.R......KRR..........KR......INER..LKTLQT.L...............VP..NG..T..............K..V.D...MS.TMLEEAVQHVKFLQ.LQIKVL....
TabHLH013 GRGSAAD........PQSLYA.K......RRR..........EK......INDR..LRVLQK.L...............VP..NG..T..............K..V.D...LS.TMLEEAVLYVKFLQ.LQIKVL....
TabHLH014 GRGSAAD........PQSLYA.K......RRR..........ER......INDR..LRVLQK.L...............VP..NG..T..............K..V.D...LS.TMLEEAVLYVKFLQ.LQIKVL....
TabHLH015 GRGSAAD........PQSLYA.K......RRR..........EK......INDR..LRALQK.L...............VP..NG..T..............K..V.D...LS.TMLEEAVLYVKFLQ.LQIKVL....
TabHLH016 AGRQTTD........PQSLYA.R......KRR..........EK......INER..LKVLQN.L...............VP..NG..T..............K..V.D...IS.TMLEEAVEYVKFLQ.LQIKLL....
TabHLH017 AGRQTTD........PQSLYA.R......KRR..........EK......INER..LKVLQN.L...............VP..NG..T..............K..V.D...IS.TMLEEAVEYVKFLQ.LQIKLL....
TabHLH018 AGRQTTD........PQSLYA.R......KRR..........EK......INER..LKVLQN.L...............VP..NG..T..............K..V.D...IS.TMLEEAVEYVKFLQ.LQIKLL....
TabHLH019 CKRPSKN........LQSLCA.K......RRR..........ER......INEK..LRELQN.L...............IP..NG..T..............K..V.D...IS.TMLEEAVQYVKFLQ.LQIKVL....
TabHLH020 CKRPSKN........LQSLCA.K......RRR..........ER......INEK..LRVLQR.L...............IP..NG..T..............K..V.D...IS.TMLEEAVQYVKFLQ.LQIKVL....
TabHLH021 CRRPSKN........LQSLYA.K......RRR..........ER......INEK..LRVLQH.L...............IP..NG..T..............K..V.D...IS.TMLEEAVQYVKFLQ.LQIKVL....
TabHLH022 KVVPTKD........PQSVVA.K......VRR..........ER......ISER..LKVLQD.L...............VP..NG..T..............K..V.D...MV.TMLEKAITYVKFLQ.LQVKVL....
TabHLH023 KVVPTKD........PQSAVA.K......VRR..........ER......ISER..LKVLQD.L...............VP..NG..T..............K..V.D...MV.TMLEKAITYVKFLQ.LQVKVL....
TabHLH024 KVVPTKD........PQSAVA.K......VRR..........ER......ISER..LKVLQD.L...............VP..NG..T..............K..V.D...MV.TMLEKAITYVKFLQ.LQVKVL....
TabHLH025 TTSPKGD........PQSLTA.K......NRR..........EK......ISER..LRTLQE.L...............VP..NG..T..............K..V.D...MV.TMLEKAFSYVKFLQ.LQVKVL....
TabHLH026 TTSPKGD........PQSLTA.K......NRR..........EK......ISER..LRTLQE.L...............VP..NG..T..............K..V.D...MV.TMLEKAFSYVKFLQ.LQVKVL....
TabHLH027 TTSPKGD........PQSLTA.K......NRR..........EK......ISER..LRTLQE.L...............VP..NG..T..............K..V.D...MV.TMLEKAFSYVQFLQ.LQVKVL....
TabHLH028 ITSPKGD........PQSLTA.K......NRR..........EK......ISER..LRTLQE.L...............VP..NG..T..............K..V.D...MV.TMLEKAFSYVKFLQ.LQVKVL....
TabHLH029 TTSPKGD........PQSLAA.K......TRR..........EK......ISER..LRTLQE.L...............VP..NG..T..............K..V.D...MV.TMLEKAFSYVKFLQ.LQVKVL....
TabHLH030 TTSPKGD........PQSLAA.K......NRR..........EK......ISER..LRTLQE.L...............VP..NG..T..............K..V.D...MV.TMLEKAVSYVKFLQ.LQVKVL....
TabHLH031 TSSPKGD........TQSLTA.K......NRR..........EK......ISER..LRTLQE.L...............VP..NG..T..............K..V.D...MV.TMLEKAFSYVKFLQ.LQVKVL....
TabHLH032 HNSPKGD........PQSLAA.K......NRR..........EK......ISER..LRTLQE.L...............VP..NG..T..............K..V.D...MV.TMLEKAVSYVKFLQ.LQVKVL....
TabHLH033 TTSAKGG........PQSHTA.K......NRR..........EK......ISER..LRTLQE.L...............VP..NG..T..............K..V.D...MV.TMLEKAFSYVKFLQ.LQVKVL....
TabHLH034 ATSSAKD........TQSDAA.K......TRR..........ER......IGER..LRVLQE.L...............VP..NG..S..............K..V.D...MV.TMLDKAITYVKFMQ.LQLTVL....
TabHLH035 PTSSAKD........PQSDAA.K......TRR..........ER......IGER..LRVLQE.L...............VP..NG..N..............K..V.D...MV.TMLDKAITYVKFMQ.LQLTVL....
TabHLH036 ATTSAKD........PQSDAA.K......TRR..........ER......IGER..LRVLQE.L...............VP..NG..N..............K..V.D...MV.TMLDKAITYVKFMQ.LQLTVL....
TabHLH037 NVRISSD........PQTVAA.R......LRR..........EK......VSER..LRALQR.L...............VP..GG..S..............K..M.D...TA.SMLDEAASYLKFLK.SQVAAL....
TabHLH038 NVRISSD........PQTVAA.R......LRR..........EK......VSER..LRALQR.L...............VP..GG..S..............K..M.D...TA.SMLDEAASYLKFLK.SQVAAL....
TabHLH039 NVRISSD........PQTVAA.R......LRR..........EK......VSER..LRALQR.L...............VP..GG..S..............K..M.D...TA.SMLDEAASYLKFLK.SQVAAL....
TabHLH040 NVRISSD........PQTVAA.R......LRR..........ER......VSDR..LRVLQK.L...............VP..GG..S..............K..M.D...TA.SMLDEAASYLKFLK.SQVQAL....
TabHLH041 NVRISSD........PQTVAA.R......LRR..........ER......VSDR..LRVLQK.L...............VP..GG..S..............K..M.D...TA.SMLDEAASYLKFLK.SQVQAL....
TabHLH042 NVRISSD........PQTVAA.R......LRR..........ER......VSDR..LRVLQK.L...............VP..GG..S..............K..M.D...TA.SMLDEAASYLKFLK.SQVQAL....
TabHLH043 NVRISSD........PQTVAA.R......QRR..........ER......ISER..LRVLQK.L...............VP..GG..A..............K..M.D...TA.SMLDEAANYLRFLK.SQLREL....
TabHLH044 NVRISSD........PQTVAA.R......QRR..........ER......ISER..LRVLQK.L...............VP..GG..A..............K..M.D...TA.SMLDEAANYLRFLK.SQIREL....
TabHLH045 NVRISSD........PQTVAA.R......QRR..........ER......ISER..LRVLQK.L...............VP..GG..A..............K..M.D...TA.SMLDEAANYLRFLK.SQIREL....
TabHLH046 NVRTSKD........PQSVAA.R......LRR..........ER......ISER..IRVLQR.L...............VP..GG..T..............K..M.D...TA.SMLDEAIHYVKFLK.SQVQSL....
TabHLH047 NVRTSKD........PQSVAA.R......LRR..........ER......ISER..IRVLQR.L...............VP..GG..T..............K..M.D...TA.SMLDEAIHYVKFLK.SQVQSL....
TabHLH048 NVRTSKD........PQSVAA.R......LRR..........ER......ISER..IRVLQR.L...............VP..GG..T..............K..M.D...TA.SMLDEAIHYVKFLK.SQV.......
TabHLH049 NVRISTD........PQSVAA.R......MRR..........ER......ISER..IRILQR.L...............VP..GG..T..............K..M.D...TA.SMLDEAIHYVKFLK.TQVQSL....
TabHLH050 NVRISTD........PQSVAA.R......MRR..........ER......ISER..IRILQR.L...............VP..GG..T..............K..M.D...TA.SMLDEAIHYVKFLK.TQVQSL....
TabHLH051 NVHISTD........PQSVAA.R......MRR..........ER......ISER..IRILQR.L...............VP..GG..T..............K..M.D...TA.SMLDEAIHYVKFLK.TQVQSL....
TabHLH052 NVRISED........PQSVAA.R......HRR..........ER......ISER..IRILQR.L...............VP..GG..T..............K..M.D...TA.SMLDEAIRYIKFLK.RQVQDL....
TabHLH053 NVRISED........PQSVAA.R......HRR..........ER......ISER..IRILQR.L...............VP..GG..T..............K..M.D...TA.SMLDEAIRYIKFLK.RQVQDL....
TabHLH054 NVRISED........PQSVAA.R......HRR..........ER......ISER..IRILQR.L...............VP..GG..T..............K..M.D...TA.SMLDEAIRYIKFLK.RQVQDL....
TabHLH055 NGRVSSE........PQSVAA.R......LRR..........ER......VSQR..MRALQR.L...............VP..GG..A..............R..L.D...TA.SMLEEAVRYVRFLK.SHVQAL....
TabHLH056 NGRVSSE........PQSVAA.R......LRR..........ER......VSQR..MRALQR.L...............VP..GG..A..............R..L.D...TA.SMLEEAVRYVRFLK.SHVQAL....
TabHLH057 NGRVSSE........PQSVAA.R......LRR..........ER......VSQR..MRALQR.L...............VP..GG..A..............R..L.D...TA.SMLEEAVRYVRFLK.SHVQAL....
TabHLH058 GAKLSTD........PQSVAA.R......ERR..........HR......ISDR..FRVLRS.L...............VP..GG..S..............K..M.D...TV.SMLEQAIHYVKFLK.AQVSLH
TabHLH059 GAKLSTD........PQSVAA.R......ERR..........HR......ISDR..FRVLRS.L...............VP..GG..S..............K..M.D...TV.SMLEQAIHYVKFLK.AQVSLH
TabHLH060 GAKLSTD........PQSVAA.R......ERR..........HR......ISDR..FRVLRS.L...............VP..GG..S..............K..M.D...TV.SMLEQAIHYVKFLK.AQVSLY
TabHLH061 KRSRAAA........IHNESE.R......KRR..........DR......INQK..MQTLQK.L...............VP..NS..S..............K..T.D...KA.SMLDEVIDHLKELQ.ATVQMM....
TabHLH062 KRSRAAA........IHNESE.R......KRR..........DR......INQK..MQTLQK.L...............VP..NS..S..............K..T.D...KA.SMLDEVIDHLKQLQ.ATVQMM....
TabHLH063 KRSRAAA........IHNESE.R......KRR..........DR......INQK..MQTLQK.L...............VP..NS..S..............K..T.D...KA.SMLDEVIDHLKQLQ.ATVQMM....
TabHLH064 KRGRTAE........VHNMSE.R......RRR..........DR......INEK..MRALQE.L...............IP..NC..N..............KI...D...KA.SMLEEAIEYLKTLQ.LQVQMM....
TabHLH065 KRGRTAE........VHNMSE.R......RRR..........DR......INEK..MRALQE.L...............IP..NC..N..............KI...D...KA.SMLEEAIEYLKTLQ.LQVQMM....
TabHLH066 KRGRTAE........VHNMSE.R......RRR..........DR......INEK..MRALQE.L...............IP..NC..N..............KV.I.D...KA.SMLEEAIEYLKTLQ.LQVQMM....
TabHLH067 RRTRAAE........VHNQSE.R......KRR..........DR......INEK..MKALQE.L...............VP..HC..N..............K..S.D...KA.SILDEAIEYLKSLQ.LQVQIM....
TabHLH068 RRTRAAE........VHNQSE.R......KRR..........DR......INEK..MKALQE.L...............VP..HC..N..............K..S.D...KA.SILDEAIEYLKSLQ.LQVQIM....
TabHLH069 RRTRAAE........VHNQSE.R......KRR..........DR......INEK..MKALQE.L...............VP..HC..N..............K..S.D...KA.SILDEAIEYLKSLQ.LQVQIM....
TabHLH070 RRSRAAE........VHNQSE.R......RRR..........DR......INEK..MRSLQE.L...............IP..HC..N..............K..A.D...KA.SILDEAIEYLKSLQ.MQVQIM....
TabHLH071 RRSRAAE........VHNQSE.R......RRR..........DR......INEK..MRSLQE.L...............IP..HC..N..............K..A.D...KA.SILDEAIEYLKSLQ.MQVQIM....
TabHLH072 RRSRAAE........VHNQSE.R......RRR..........DR......INEK..MRSLQE.L...............IP..HC..N..............K..A.D...KA.SILDEAIEYLKSLQ.MQVQIM....
TabHLH073 RRSRAAE........VHNLSE.R......RRR..........DR......INEK..MRALQE.L...............IP..HC..N..............K..T.D...KA.SMLDEAIEYLKTLQ.MQVQMM....
TabHLH074 RRSRAAE........VHNLSE.R......RRR..........DR......INEK..MRALQE.L...............IP..HC..N..............K..T.D...KA.SMLDEAIEYLKTLQ.MQVQMM....
TabHLH075 RRSRAAE........VHNLSE.R......RRR..........DR......INEK..MRALQE.L...............IP..HC..N..............K..T.D...KA.SMLDEAIEYLKTLQ.MQVQMM....
TabHLH076 KRRRAAQ........VHNLSE.R......RRR..........DR......INEK..MRALQE.L...............VP..HC..N..............KVQT.D...KA.SMLDEAIEYLKSLQ.LQLQVM....
TabHLH077 KRRRAAQ........VHNLSE.R......RRR..........DR......INEK..MRALQE.L...............VP..HC..N..............K..T.D...KA.SMLDEAIEYLKSLQ.LQLQLM....
TabHLH078 KRRRAAQ........VHNLSE.R......RRR..........DR......INEK..MRALQE.L...............VP..HC..N..............K..T.D...KA.SMLDEAIEYLKSLQ.LQLQLM....
TabHLH079 RRSRSAD........FHNFSE.R......RRR..........DK......INEK..LKALQE.L...............LP..NC..T..............K..T.D...KV.SMLDEAIDYLKSLQ.LQLQML....
TabHLH080 RRSRSAD........FHNFSE.R......RRR..........DK......INEK..LKALQE.L...............LP..NC..T..............K..T.D...KV.SMLDEAIDYLKSLQ.LQLQML....
TabHLH081 RRSRSAD........FHNFSE.R......RRR..........DK......INEK..LKALQE.L...............LP..NC..T..............K..T.D...KV.SMLDEAIDYLKSLQ.LQLQML....
TabHLH082 RRSRSAD........FHNFSE.R......RRR..........DK......INEK..LKALQE.L...............LP..NC..T..............K..T.D...KV.SMLDEVIDYLKSLQ.LQLQML....
TabHLH083 KRARAAE........VHNLSE.K......RRR..........CK......INEK..MKALQS.L...............VP..NS..S..............KVQT.D...KA.SMLDDAIEYLKHLQ.LQVQML....
TabHLH084 KRARAAE........VHNLSE.K......RRR..........CK......INEK..MKALQS.L...............VP..DS..S..............K..T.D...KA.SMLDDAIEYLKHLQ.LQVQML....
TabHLH085 KRARAAE........VHNLSE.K......RRR..........CK......INEK..MKALQS.L...............VP..NS..S..............K..T.D...KA.SMLDDAIEYLKHLQ.LQVQML....
TabHLH086 RRGQATD........SHSLAE.R......VRR..........EK......IGER..MKLLQS.L...............VP..SC..N..............KI.T.G...KA.LMLDEIINYVQSLQ.RQVEFL....
TabHLH087 RRGQATD........SHSLAE.R......VRR..........EK......IGER..MKLLQS.L...............VP..SC..N..............KI.T.G...KA.LMLDEIINYVQSLQ.RQVEFL....
TabHLH088 RRGQATD........SHSLAE.R......VRR..........EK......IGER..MKLLQS.L...............VP..SC..N..............KI.T.G...KA.LMLDEIINYVQSLQ.RQVEFL....
TabHLH089 RRGEATD........SHSLAE.R......VRR..........EK......ISQR..MKLLQD.L...............VP..GC..N..............KV.V.G...KA.VMLDEIINYVQSLQ.RQVEFL....
TabHLH090 RRGEATD........SHSLAE.R......VRR..........EK......ISQR..MKLLQD.L...............VP..GC..N..............KV.V.G...KA.VMLDEIINYVQSLQ.RQVEFL....
TabHLH091 RRGEATD........SHSLAE.R......VRR..........EK......ISQR..MKLLQD.L...............VP..GC..N..............KV.V.G...KA.VMLDEIINYVQSLQ.RQVEFL....
TabHLH092 RRGQATD........SHSLAE.R......VRR..........ER......ISQR..MKFLQD.L...............VP..GC..N..............KV.I.G...KA.LMLDEIINYVQSLQ.RQVEFL....
TabHLH093 RRGQATD........SHSLAE.R......VRR..........ER......ISQR..MKFLQD.L...............VP..GC..N..............KV.I.G...KA.LMLDEIINYVQSLQ.RQVEFL....
TabHLH094 RRGQATD........SHSLAE.R......VRR..........ER......ISQR..MKFLQD.L...............VP..GC..N..............KV.I.G...KA.LMLDEIINYVQSLQ.RQVEFL....
TabHLH095 KRGQATN........SHSLAE.R......LRR..........KK......ISEK..MKLLQD.L...............VP..GC..N..............KI.T.G...KA.VMLDEIINYVQSLQ.QQVEFL....
TabHLH096 KRGQATN........SHSLAE.R......LRR..........KK......ISER..MKLLQD.L...............VP..GC..N..............KI.T.G...KA.VMLDEIINYVQSLQ.RQVEFL....
TabHLH097 KRGQATN........SHSLAE.R......LRR..........KK......ISER..MKLLQD.L...............VP..GC..N..............KI.T.G...KA.VMLDEIINYVQSLQ.RQVEFL....
TabHLH098 RRGQATN........SHSLAE.R......LRR..........EK......ISER..MKLLQD.L...............VP..GC..S..............KV.T.G...KA.VMLDEIINYVQSLQ.RQVEFL....
TabHLH099 RRGQATN........SHSLAE.R......LRR..........EK......ISER..MKLLQD.L...............VP..GC..S..............KV.T.G...KA.VMLDEIINYVQSLQ.RQVEFL....
TabHLH100 RRGQATN........SHSLAE.R......LRR..........EK......ISER..MKLLQD.L...............VP..GC..S..............KV.T.G...KA.VMLDEIINYVQSLQ.RQVEFL....
TabHLH101 RSGQATN........SHSLAE.R......VRR..........EK......ISER..MKFLQD.L...............VP..GC..S..............KV.I.G...KA.VMLDEIINYVQSLQ.RQVEFL....
TabHLH102 RSGQATN........SHSLAE.R......VRR..........EK......ISER..MKFLQD.L...............VP..GC..S..............KV.I.G...KA.VMLDEIINYVQSLQ.RQVEFL....
TabHLH103 RSGQATN........SHSLAE.R......VRR..........EK......ISER..MKFLQD.L...............VP..GC..S..............KV.I.G...KA.VMLDEIINYVQSLQ.RQVEFL....
TabHLH104 RRGQATD........SHSLAE.R......VRR..........EK......ITLK..MKMLQD.L...............VP..GC..N..............KV.I.G...KA.LMLDEIINYVQSLQ.QQVEFL....
TabHLH105 RRGQATD........SHSLAE.R......VRR..........EK......ITLK..MKMLQD.L...............VP..GC..N..............KV.I.G...KA.LMLDEIINYVQSLQ.QQVEFL....
TabHLH106 RRGQATD........SHSLAE.R......VRR..........EK......ITLK..MKMLQD.L...............VP..GC..N..............KV.I.G...KA.LMLDEIINYVQSLQ.QQVEFL....
TabHLH107 KRGQATN........NHSLAE.R......FRR..........EK......INER..MKHLQD.L...............VP..GC..N..............KI.T.G...KA.MMLDEIINYVQSLQ.RQVEFL....
TabHLH108 KRGQATN........NHSLAE.R......FRR..........EK......INER..MKHLQD.L...............VP..GC..N..............KI.T.G...KA.MMLDEIINYVQSLQ.RQVEFL....
TabHLH109 KRGQATN........NHSLAE.R......FRR..........EK......INER..MKHLQD.L...............VP..GC..N..............KI.T.G...KA.MMLDEIINYVQSLQ.RQVEFL....
TabHLH110 RRGQATD........SHSLSE.R......VRR..........ER......ISER..MRMLQS.L...............VP..GC..D..............KV.T.G...KA.LILDEIINYVQSLQ.NQVEFL....
TabHLH111 RRGQATD........SHSLSE.R......VRR..........ER......ISER..MRMLQS.L...............VP..GC..D..............KV.T.G...KA.LILDEIINYVQSLQ.NQVEFL....
TabHLH112 RRGQATD........SHSLSE.R......VRR..........ER......ISER..MRMLQS.L...............VP..GC..D..............KV.T.G...KA.LILDEIINYVQSLQ.NQVEFL....
TabHLH113 RRGQATD........SHSLAE.R......VRR..........ER......ISER..MRLLQT.L...............VP..GC..D..............KV.T.G...KA.LVLDEIINYVQSLQ.NQVEFL....
TabHLH114 RRGQATD........SHSLAE.R......VRR..........ER......ISER..MRLLQT.L...............VP..GC..D..............KV.T.G...KA.LVLDEIINYVQSLQ.NQVEFL....
TabHLH115 RRGQATD........SHSLAE.R......VRR..........ER......ISER..MRYLQE.L...............VP..GC..D..............KV.T.G...KA.GMLDEIINYVQSLQ.KQVEFL....
TabHLH116 RRGQATD........SHSLAE.R......VRR..........ER......ISER..MRYLQE.L...............VP..GC..D..............KV.T.G...KA.GMLDEIINYVQSLQ.KQVEFL....
TabHLH117 RRGQATD........SHSLAE.R......VRR..........ER......ISER..MRYLQE.L...............VP..GC..D..............KV.T.G...KA.GMLDEIINYVQSLQ.KQVEFL....
TabHLH118 RRGQATD........SHSLAE.R......ARR..........EK......ITER..MKILQD.L...............VP..GC..N..............KV.I.G...KA.SVLDEIINYVQALE.RQIEFL....
TabHLH119 RRGQATD........SHSLAE.R......ARR..........EK......ITER..MKILQD.L...............VP..GC..N..............KV.I.G...KA.SVLDEIINYVQALE.RQIEFL....
TabHLH120 RRGQATD........SHSLAE.R......ARR..........EK......ITER..MKILQD.L...............VP..GC..N..............KV.I.G...KA.SVLDEIINYVQALE.RQIEFL....
TabHLH121 RRGQATD........SHSLAE.R......ARR..........EK......INAR..MELLKE.L...............VP..GC..S..............KV.S.G...TA.LVLEEIINHVQSLQ.RQVEYL....
TabHLH122 RRGQATD........SHSLAE.R......ARR..........EK......INAR..MELLKE.L...............VP..GC..S..............KV.S.G...TA.LVLEEIINHVQSLQ.RQVEYL....
TabHLH123 RRGQATD........SHSLAE.R......ARR..........EK......INAR..MELLKE.L...............VP..GC..S..............KV.S.G...TA.LVLEEIINHVQSLQ.RQVEYL....
TabHLH124 ........................................................MRALQE.L...............VP..NT..N..............K..T.D...RA.VMLDEILDYVKFLR.LQVKVL....
TabHLH125 RRGQATD........PHSIAE.R......LRR..........ER......IAER..MRALQE.L...............VP..NT..N..............K..T.D...RA.VMLDEILDYVKFLR.LQVKVL....
TabHLH126 RRGQATD........PHSIAE.R......LRR..........ER......IAER..MRALQE.L...............VP..NT..N..............K..T.D...RA.VMLDEILDYVKFLR.LQVKVL....
TabHLH127 RRGQATD........PHSIAE.R......LRR..........ER......IAER..MKALQE.L...............VP..NA..N..............K..T.D...KA.SMLDEIIDYVKFLQ.LQVKVL....
TabHLH128 RRGQATD........PHSIAE.R......LRR..........ER......IAER..MKALQE.L...............VP..NA..N..............K..T.D...KA.SMLDEIIDYVKFLQ.LQVKVL....
TabHLH129 RRGQATD........PHSIAE.R......LRR..........ER......IAER..MKALQE.L...............VP..NA..N..............K..T.D...KA.SMLDEIIDYVKFLQ.LQVKVL....
TabHLH130 RRGQATD........PHSIAE.R......LRR..........ER......IAER..MKALQE.L...............VP..NA..N..............K..T.D...KA.SMLDEIIDYVKFLQ.LQVKVL....
TabHLH131 RRGQATD........PHSIAE.R......LRR..........ER......IAER..MKSLQE.L...............VP..NA..N..............K..T.D...KA.SMLDEIIDYVKFLQ.LQVKVL....
TabHLH132 RRGQATD........PHSIAE.R......LRR..........ER......IAER..MKSLQE.L...............VP..NA..N..............K..T.D...KA.SMLDEIIDYVKFLQ.LQVKVL....
TabHLH133 RRGQATD........PHSIAE.R......LRR..........ER......IAER..MKSLQE.L...............VP..NA..N..............K..T.D...KA.SMLDEIIDYVKFLQ.LQVKVL....
TabHLH134 RRGQATD........PHSIAE.R......LRR..........ER......IAER..MKALQE.L...............VP..SA..N..............K..T.D...KA.SMLDEIIDYVKFLQ.LQVKVL....
TabHLH135 RRGQATD........PHSIAE.R......LRR..........ER......IAER..MKALQE.L...............VP..SA..N..............K..T.D...KA.SMLDEIIDYVKFLQ.LQVKVL....
TabHLH136 RRGQATD........PHSIAE.R......LRR..........EK......ISER..MKNLQD.L...............VP..NS..N..............K..A.D...KS.SMLDEIIDYVKFLQ.LQVKVL....
TabHLH137 RRGQATD........PHSIAE.R......LRR..........EK......ISER..MKNLQD.L...............VP..NS..N..............K..A.D...KS.SMLDEIIDYVKFLQ.LQVKVL....
TabHLH138 RRGQATD........PHSIAE.R......LRR..........EK......ISER..MKNLQD.L...............VP..NS..N..............K..A.D...KS.SMLDEIIDYVKFLQ.LQVKVL....
TabHLH139 KRGCATH........PRSIAE.R......ERR..........TR......ISKR..LRRLQD.L...............VP..NM..D..............K.QT.N...TS.DMLDIAVDYIKVLQ.DQIEKL....
TabHLH140 KRGCATH........PRSIAE.R......ERR..........TR......ISKR..LRRLQD.L...............VP..NM..D..............K.QT.N...TS.DMLDIAVDYIKVLQ.DQIEKL....
TabHLH141 KRGCATH........PRSIAE.R......ERR..........TR......ISKR..LRRLQD.L...............VP..NM..D..............K.QT.N...TS.DMLDIAVDYIKVLQ.DQIEKL....
TabHLH142 KRGCATH........PRSIAE.R......VRR..........TK......ISER..IRKLQE.L...............VP..NM..E..............K.QT.N...TS.DMLDLAVDYIKELQ.MQVKAM....
TabHLH143 KRGCATH........PRSIAE.R......VRR..........TK......ISER..IRKLQE.L...............VP..NM..E..............K.QT.N...TS.DMLDLAVDYIKELQ.MQVKVM....
TabHLH144 KRGCATH........PRSIAE.R......VRR..........TK......ISER..IRKLQE.L...............VP..NM..E..............K.QT.N...TS.DMLDLAVDYIKELQ.MQVKVM....
TabHLH145 KRGCATH........PRSIAE.R......VRR..........TR......ISER..IRKLQE.L...............VP..NM..D..............K.QT.N...TA.DMLDLAVDYIKELQ.EQVKVI....
TabHLH146 KRGCATH........PRSIAE.R......VRR..........TR......ISER..IRKLQE.L...............VP..NM..D..............K.QT.N...TA.DMLDLAVDYIKELQ.EQVKVI....
TabHLH147 KRGCATH........PRSIAE.R......VRR..........TR......ISER..IRKLQE.L...............VP..NM..D..............K.QT.N...TA.DMLDLAVDYIKELQ.EQVKVI....
TabHLH148 KRGCATH........PRSIAE.R......ERR..........TR......ISDK..LRKLQD.L...............VP..NM..D..............K.QT.S...TS.DMLDLAVEHIKGLQ.SQLQAM....
TabHLH149 KRGCATH........PRSIAE.R......ERR..........TR......ISDK..LRKLQD.L...............VP..NM..D..............K.QT.S...TS.DMLDLAVEHIKGLQ.SQLQAM....
TabHLH150 KRGCATH........PRSIAE.R......ERR..........TR......ISYK..LRKLQD.L...............VP..NM..D..............K.QT.S...TS.DMLDLAVEHIKGLQ.SQLQAM....
TabHLH151 KKDRASD........GRSIAE.R......VRR..........TK......ISER..LRNLQG.L...............VP..NM..E..............K.QT.N...TS.DMLDMAVDYIKELQ.MKIKAM....
TabHLH152 RRSCSGK...........AAE.Q......RRR..........DK......INDK..LRTLQQ.L...............TP..RC..S..............K..T.D...KA.STLEEVIKYVKSLQ.HQIQMM....
TabHLH153 RRSSCTG........SHSQTE.K......RRR..........AK......INKS..LKTLQR.I...............VP..GC...D.............K.RC.S...QA.STLDLTIKYIKSLQ.RQVQDM....
TabHLH154 RRTASGG........TQSQTE.K......RRR..........SK......INKS..LKTLQH.L...............VP..GC..DD.............K.RC.S...QA.STLDLTIRYIKSLQ.RQVHAM....
TabHLH155 RPSHHSE........THNSTE.K......RRR..........CK......ISDR..LRTLQQ.L...............VP..GC..D..............K..T.N...QE.STLDQTIKYMKSLQ.QQVQAM....
TabHLH156 EAR.SLK........VHSLSE.R......RRR..........ER......INTH..LATLRR.M...............IP..DA..N..............Q..M.D...KA.TLLACVVNQVKELK.RKATET....
TabHLH157 EVR.SLK........VHSESE.R......RRR..........ER......INTH..LATLRR.M...............IP..DA..N..............Q..M.D...KA.TLLACVVNQVKELK.RKATET....
TabHLH158 EVR.SLK........VHSESE.R......RRR..........ER......INTH..LATLRR.M...............IP..DA..N..............Q..M.D...KA.TLLACVVNQVKELK.RKATET....
TabHLH159 EAR.ALK........VHSEAE.R......RRR..........VR......INAH..LTALRR.M...............IP..DT..K..............Q..M.D...KA.ALLARVVDQVRHLK.RRASEA....
TabHLH160 EAR.ALK........VHSEAE.R......RRR..........VR......INAH..LTALRR.M...............IP..DT..K..............Q..M.D...KA.ALLARVVDQVKHLK.RRAGEA....
TabHLH161 EAR.ALK........VHSEAE.R......RRR..........VR......INAH..LTALRR.M...............IP..DT..K..............Q..M.D...KA.ALLARVVDQVKHLK.RRAGEA....
TabHLH162 EAR.ALK........VHSEAE.R......RRR..........VR......INAH..LTALRR.M...............IP..DT..K..............Q..M.D...KA.ALLGRVVDQVRHLK.RRASEV....
TabHLH163 EAR.ALK........VHSEAE.R......RRR..........VR......INAH..LTTLRR.M...............IP..DT..N..............Q..M.D...KA.ALLARVVDQVRHVK.RRATEA....
TabHLH164 EAR.ALK........AHSEAE.R......RRR..........VR......INAH..LTALRR.M...............IP..DT..K..............Q..M.D...KA.ALLARVVDQVRQLK.RRASEA....
TabHLH165 GKGATVR........SHSEAE.R......KRR..........QR......INAH..LATLRT.L...............VP..SA..S..............R..M.D...KA.ALLGEVVRHVRELQ.GRANDA....
TabHLH166 GKGATVR........SHSEAE.R......KRR..........QR......INAH..LATLRT.L...............VP..SA..S..............R..M.D...KA.ALLGEVVRHVRELQ.GRANDA....
TabHLH167 GKGATVR........SHSEAE.R......KRR..........QR......INAH..LATLRT.L...............VP..SA..S..............R..M.D...KA.ALLGEVVRHVRELQ.GRANDA....
TabHLH168 RGARTSR........SHSEAE.R......KRR..........QR......INTH..LATLRS.L...............LP..SA..S..............Q..M.D...KA.ALLGEVVRHVRELR.GDADGG....
TabHLH169 RGARTSR........SHSEAE.R......KRR..........QR......INTH..LATLRS.L...............LP..SA..S..............Q..M.D...KA.ALLGEVVRHVRELR.GDADGG....
TabHLH170 RGARTSR........SHSEAE.R......KRR..........QR......INTH..LATLRS.L...............LP..SA..S..............Q..M.D...KA.ALLGEVVRHVRELR.GDADGG....
TabHLH171 KAATALR........SHSEAE.R......RRR..........ER......INAH..LATLRS.M...............VP..CT..D..............K..M.D...KA.ALLAEVINHVKKLK.SEAARV....
TabHLH172 KAATALR........SHSEAE.R......RRR..........ER......INAH..LATLRS.M...............LP..CT..D..............K..M.D...KA.ALLAEVINHVKKLK.SEAARV....
TabHLH173 KAATALR........SHSEAE.R......RRR..........ER......INAH..LATLRS.M...............VP..CT..G..............K..M.D...KA.ALLAEVINHVKKLK.SEAARV....
TabHLH174 KALAASK........SHSEAE.R......RRR..........ER......INSH..LARLRS.L...............LP..NT..T..............K..T.D...KA.SLLAEVIQHVKELK.RQTSEI....
TabHLH175 KALAASK........SHSEAE.R......RRR..........ER......INSH..LARLRS.L...............LP..NT..T..............K..T.D...KA.SLLAEVIQHVKELK.RQTSEI....
TabHLH176 KALAASK........SHSEAE.R......RRR..........ER......INSH..LARLRS.L...............LP..NT..T..............K..T.D...KA.SLLAEVIQHVKELK.RQTSEI....
TabHLH177 KALAASR........SHSEAE.R......RRR..........QR......INGH..LARLRS.L...............LP..NT..T..............K..T.D...KA.SLLAEVLEHVKELK.RQTSAM....
TabHLH178 KALAASR........SHSEAE.R......RRR..........QR......INGH..LARLRS.L...............LP..NT..T..............K..T.D...KA.SLLAEVLEHVKELK.RQTSAM....
TabHLH179 KALAASR........SHSEAE.R......RRR..........QR......INGH..LARLRS.L...............LP..NT..T..............K..T.D...KA.SLLAEVLEHVKELK.RQTSAM....
TabHLH180 RAVAATR........NHREAE.K......RRR..........ER......IKSH..LDRLRA.V...............LA..CD..P..............K..I.D...KA.TLLAKAVERVRELK.QRMDGV....
TabHLH181 RAVVATR........NHREAE.K......RRR..........ER......IKSH..LDRLRA.V...............LA..CD..P..............K..I.D...KA.TLLAKAVERVRELK.QRMAGV....
TabHLH182 RAVAATR........NHREAE.K......RRR..........ER......IKSH..LDRLRA.V...............LA..CD..P..............K..I.D...KA.TLLAKAVERVRELK.QRMAGV....
```

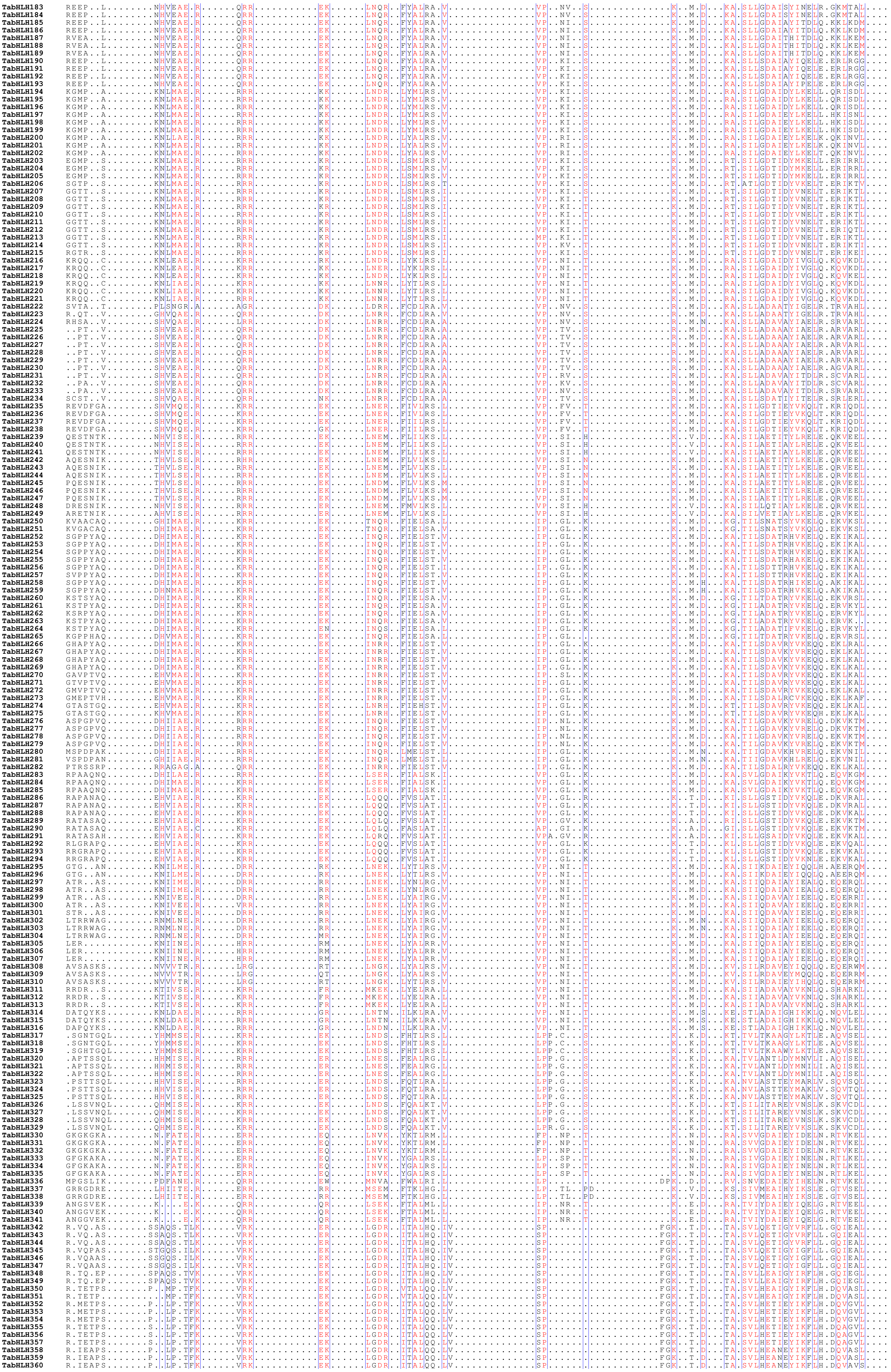
TabHLH183
TabHLH184
TabHLH185
TabHLH186
TabHLH187
TabHLH188
TabHLH189
TabHLH190
TabHLH191
TabHLH192
TabHLH193
TabHLH194
TabHLH195
TabHLH196
TabHLH197
TabHLH198
TabHLH199
TabHLH200
TabHLH201
TabHLH202
TabHLH203
TabHLH204
TabHLH205
TabHLH206
TabHLH207
TabHLH208
TabHLH209
TabHLH210
TabHLH211
TabHLH212
TabHLH213
TabHLH214
TabHLH215
TabHLH216
TabHLH217
TabHLH218
TabHLH219
TabHLH220
TabHLH221
TabHLH222
TabHLH223
TabHLH224
TabHLH225
TabHLH226
TabHLH227
TabHLH228
TabHLH229
TabHLH230
TabHLH231
TabHLH232
TabHLH233
TabHLH234
TabHLH235
TabHLH236
TabHLH237
TabHLH238
TabHLH239
TabHLH240
TabHLH241
TabHLH242
TabHLH243
TabHLH244
TabHLH245
TabHLH246
TabHLH247
TabHLH248
TabHLH249
TabHLH250
TabHLH251
TabHLH252
TabHLH253
TabHLH254
TabHLH255
TabHLH256
TabHLH257
TabHLH258
TabHLH259
TabHLH260
TabHLH261
TabHLH262
TabHLH263
TabHLH264
TabHLH265
TabHLH266
TabHLH267
TabHLH268
TabHLH269
TabHLH270
TabHLH271
TabHLH272
TabHLH273
TabHLH274
TabHLH275
TabHLH276
TabHLH277
TabHLH278
TabHLH279
TabHLH280
TabHLH281
TabHLH282
TabHLH283
TabHLH284
TabHLH285
TabHLH286
TabHLH287
TabHLH288
TabHLH289
TabHLH290
TabHLH291
TabHLH292
TabHLH293
TabHLH294
TabHLH295
TabHLH296
TabHLH297
TabHLH298
TabHLH299
TabHLH300
TabHLH301
TabHLH302
TabHLH303
TabHLH304
TabHLH305
TabHLH306
TabHLH307
TabHLH308
TabHLH309
TabHLH310
TabHLH311
TabHLH312
TabHLH313
TabHLH314
TabHLH315
TabHLH316
TabHLH317
TabHLH318
TabHLH319
TabHLH320
TabHLH321
TabHLH322
TabHLH323
TabHLH324
TabHLH325
TabHLH326
TabHLH327
TabHLH328
TabHLH329
TabHLH330
TabHLH331
TabHLH332
TabHLH333
TabHLH334
TabHLH335
TabHLH336
TabHLH337
TabHLH338
TabHLH339
TabHLH340
TabHLH341
TabHLH342
TabHLH343
TabHLH344
TabHLH345
TabHLH346
TabHLH347
TabHLH348
TabHLH349
TabHLH350
TabHLH351
TabHLH352
TabHLH353
TabHLH354
TabHLH355
TabHLH356
TabHLH357
TabHLH358
TabHLH359
TabHLH360

```
TabHLH361   S.RPATN.......NNILP.SFK.......VRK...........EK......LGDR..IAALQQ.LV..............SP....................FGK..T.D...TA.SVLMEAIGYIKFLQ.DQVETL....
TabHLH362   S.RPATN.......NNILP.SFK.......VRK...........EK......LGDR..IAALQQ.LV..............SP....................FGK..T.D...TA.SVLMEAIGYIKFLQ.DQVETL....
TabHLH363   S.RPATN.......NNILP.SFK.......VRK...........EK......LGDR..IAALQQ.MV..............SP....................FGK..T.D...TA.SVLMEAIGYIKFLQ.DQVETL....
TabHLH364   A.PPAAS.......SNKRP...R.......ARK...........ER......LGER..ILALQQ.LV..............SP....................FGK..T.D...TA.SVLHEALGYIRFLH.DQVQVL....
TabHLH365   A.PPAAS.......TNKRP...R.......ARK...........ER......LGER..ILALQQ.LV..............SP....................FGK..T.D...TA.SVLHEALGYIRFLH.DQVQVL....
TabHLH366   A.APAAT.......SNKRP...R.......ARK...........ER......LGER..ILALQQ.LV..............SP....................FGK..T.D...TA.SVLHEALGYIRFLH.DQVQVL....
TabHLH367   .NA.SPR.......GGGGPISPK.......EKK...........DK......VGER..VAALQQ.LV..............SP....................FGK..T.D...TA.SVLQEASGYIKFLH.QQLEVL....
TabHLH368   .NA.SPR.......GSGGPISPK.......EKK...........DK......VGER..VAALQQ.LV..............SP....................FGK..T.D...TA.SVLQEASGYIKFLH.QQLEVL....
TabHLH369   .SA.SPR.......GSGGPISPR.......EKK...........DK......VGER..VAALQQ.LV..............SP....................FGK..T.D...TA.SVLQEASGYIKFLH.QQLEVL....
TabHLH370   RNA.SPR.......G.GGPISPR.......EKK...........DK......VGER..VAALQQ.LV..............SP....................FGK..T.D...TA.SVLQEASGYIKFLH.QQLEVL....
TabHLH371   ...KDTS.......SRSPP.KAE.......VPD...........MK......LGERDKIIALQQ.II..............SP....................YGK..T.D...RA.SVLYETIKHIEYLH.EQIQLL....
TabHLH372   ...KDTS.......SRSPP.KAE.......VPD...........MK......LGERDKIIALQQ.II..............SP....................YGK..T.D...RA.SVLYETIKHIEYLH.EQIQLL....
TabHLH373   ...KDTS.......SRSPP.KAE.......VPD...........MK......LGERDKIIALQQ.II..............SP....................YGK..T.D...RA.SVLYETIKHIEYLH.EQIQLL....
TabHLH374   HESCSPT.......SSSPK..GQ.......MPK...........VK......LGHK..ITALQQ.MV..............SP....................FGK..T.D...TA.SVLYEAINYIKWLH.EQVQLL....
TabHLH375   HESCSPT.......SSSPK..GQ.......VTK...........VK......LGHK..ITALQQ.MV..............SP....................FGK..T.D...TA.SVLYEAINYIKWLH.EQVQLL....
TabHLH376   KQEAS.T.......ASPPK..QP.......VPK...........VK......LGEK..ITALQQ.IV..............SP....................FGK..T.D...TA.SVLFETIKYIKFLH.EQVQLL....
TabHLH377   KQEAS.T.......ASPPK..QP.......VPK...........VK......LGEK..ITALQQ.IV..............SP....................FGK..T.D...TA.SVLFETIKYIKFLH.EQVQLL....
TabHLH378   KQEAS.T.......ASPPK..QP.......VPK...........VK......LGEK..ITALQQ.IV..............SP....................FGK..T.D...TA.SVLFETIKYIKFLH.EQVQLL....
TabHLH379   RN..TTA.......AAKA...PC.......KRS...........QK......LGDK..ITALQQ.LV..............SP....................YGK..T.D...TA.SVLHEAAACIRNLH.DQIQIL....
TabHLH380   RN..TTA.......AAKA...PC.......KRS...........QK......LGDK..ITALQQ.LV..............SP....................YGK..T.D...TA.SVLHEAAACIRNLH.DQIQIL....
TabHLH381   RNTTTTA.......AAKA...PC.......KRS...........QK......LGDK..ITALQQ.LV..............SP....................YGK..T.D...TA.SVLHEAAACIRNLH.DQIQIL....
TabHLH382   QANPCLR........VNKLSE.K.......RRR...........SR......TNEK..MKALQS.L...............IP..NS..N...............K..T.D...KA.SMLDEHKRSAGDFF.DVHEFA....
TabHLH383   SGG..............GGGE.........RKK...........NR......MKKM..MKTLKG.I...............IP..GG..D...............R..M.D...TP.AALDEAVKYLKSLK.VEAKKH....
TabHLH384   SGG..............GGGE.........RKK...........NR......MKKM..MKTLKG.I...............IP..GG..D...............R..M.D...TP.AALDEAVKYLKSLK.VEAKKH....
TabHLH385   SGG..............GGGE.........RKK...........NR......MKKM..MKTLKG.I...............IP..GG..D...............R..M.D...TP.AALDEAVKYLKSLK.VEAKKH....
TabHLH386   TGNATGA........G.GGGE.........RKK...........ER......MKKM..MKTLKG.I...............IP..GG..D...............R..M.D...TP.AVLDEAVRYLKSLK.VEVKKL....
TabHLH387   TGNAAGA........G.GGGE.........RKK...........ER......MKKM..MKTLKG.I...............IP..GG..D...............R..M.D...TP.AVLDEAVRYLKSLK.VEVKKL....
TabHLH388   TGNAAGA........G.GGGE.........RKK...........ER......MKKM..MKTLKG.I...............IP..GG..D...............R..M.D...TP.AVLDEAVRYLKSLK.VEVKKL....
TabHLH389   .HRASPG.....ASSSGS..T.S.......AQA...........PA......LARK..AKALGR.L...............VP..GC..R...............KL.......FP.ALLSEASDYIAALE.MQVRAM....
TabHLH390   .RPASPG.....ASSSGS..T.S.......AQA...........QT......LARK..AKTLGR.L...............VP..GC..R...............KL.......FP.ALLSEASDYIAALE.MQVRAM....
TabHLH391   ..RASPG.....ASSSGST.T.S.......SSA...........PA......LARK..AKALGR.L...............VP..GC..R...............KL.......FP.ALLSEASDYIAALE.MQVRAM....
TabHLH392   ARH..PS.....VAAAAQP.Q.P.......GKA...........PA......LAKK..AKVLGR.L...............VP..GC..H...............KL.......FP.ALLSEASDYIRALE.MQVRAM....
TabHLH393   ARH..PS.....VAAAAQP.Q.P.......GKA...........PA......LAKK..AKVLGR.L...............VP..GC..H...............KL.......FP.ALLSEASDYIKALE.MQVRAM....
TabHLH394   ARH..SS.....VAAAAQP.Q.P.......GKA...........PA......LAKK..AKVLGR.L...............VP..GC..H...............KL.......FP.ALLSEASDYIKALE.MQVRAM....
TabHLH395   ...SKAA.....AAASASEPP.L.......LKE...........KK......VKDR..LRVLGR.L...............VP..GC..R...............KL.......AP.DLLEETADYVAALE.MQVKAM....
TabHLH396   ...SKAA.....AAASAGEPP.L.......LKE...........RK......VKDR..LRVLGR.L...............VP..GC..R...............KL.......AP.DLLEETADYVAALE.MQVKAM....
TabHLH397   ...SKAA.....AAASAGEPP.L.......LKE...........RK......VKDR..LRVLGR.L...............VP..GC..R...............KL.......AP.DLLEETADYVAALE.MQVKAM....
TabHLH398   AAGNG.........SSQSAAA.R.......ERR...........RR......ISEK..TAELSR.L...............IP..GG..H...............KL.......TA.EMLQEAARHVKLLQ.AQVGML....
TabHLH399   AAGNG.........SSQSAAA.R.......ERR...........RR......ISEK..TAELSR.L...............IP..GG..H...............KL.......TA.EMLQEAARHVKLLQ.AQVGML....
TabHLH400   ...NGRV.....GQTAPSAAA.R.......ERR...........RR......ISEK..TAELSR.L...............VP..GG..H...............RL.......TA.EMLQEAGRHVKLLQ.AQVGVL....
TabHLH401   ..ESSAQ.P.....GTKACRE.K.......LRR...........DR......LNER..FNELCA.VL..............EP..GK..PP..............K..A.D...KV.AILSDATRLLDQLR.AEAQKL....
TabHLH402   ..ESSAQ.P.....GTKACRE.K.......LRR...........DR......LNER..FSELCA.VL..............EP..GK..PP..............K..A.D...KV.AILSDATRLLDQLR.AEAQKL....
TabHLH403   ..ESSAQ.P.....GTKACRE.K.......LRR...........DR......LNER..FSELCA.VL..............EP..GK..PP..............K..A.D...KV.AILSDATRLLDQLR.AEAQKL....
TabHLH404   ..ETGAQ.P.....SSKACRE.K.......VRR...........DK......LNER..FLELGA.VL..............DP..GK..TP..............K..I.D...KC.AILNDAIRAVTELR.SEAEKL....
TabHLH405   ..ETGAQ.P.....SSKACRE.K.......VRR...........DK......LNER..FLELGA.VL..............DP..GK..TP..............K..I.D...KC.AILNDAIRAVTELR.SEAEKL....
TabHLH406   ..ETGAQ.P.....SSKACRE.K.......VRR...........DK......LNER..FLELSA.VL..............DP..GK..TP..............K..I.D...KC.AILNDAIRAVTELR.SEAEKL....
TabHLH407   ..EPCGR.P.....TSKACRE.K.......VRR...........DK......LNDR..FLELGT.TL..............DP..GK..PV..............K..A.D...KA.AILSDATRMVTQLR.AEAQQL....
TabHLH408   ..EPCGR.P.....TSKACRE.K.......VRR...........DK......LNDR..FLELGT.TL..............DP..GK..PV..............K..A.D...KA.AILSDATRMVTQLR.AEAQQL....
TabHLH409   ..EPCGR.P.....TSKACRE.K.......VRR...........DK......LNDR..FLELGT.TL..............DP..GK..PV..............K..A.D...KA.AILSDATRMVTQLR.AEAQQL....
TabHLH410   ...PSSSGP.....KSKACRE.K.......MRR...........DK......LNDR..FLELCS.VMNSGKHSGLEECSASNP..GK..NA..............K..L.D...KA.SILSDATRMLTQLR.GETEKL....
TabHLH411   ...PSSSGP.....KSKACRE.K.......MRR...........DK......LNDR..FLELCS.VMNSGKHTGLEECSASNP..GK..NA..............K..L.D...KA.SILSDATRMLTQLR.GETEKL....
TabHLH412   ...PSPSGP.....KSKACRE.K.......MRR...........DK......LNDR..FLELCS.VMNSGKHTGVEECSASNP..GK..NA..............K..L.D...KA.SILSDATRMLTQLR.GETEKL....
TabHLH413   ..EKVPK.K.....IHKSERE.K.......RKR...........GT......QNDL..FNELGA.ML..............EP..DR..Q...............N..N.G...KA.CVLGDTTRILKDLV.SQVESL....
TabHLH414   ..KKVPK.K.....IHKSERE.K.......RKR...........GT......QNDL..FNELGA.ML..............EP..DR..Q...............N..N.G...KA.CVLGDTTRILKDLV.SQVESL....
TabHLH415   ..TKVPN.K.....IHKSERE.K.......RKR...........GT......QNDL..FNELGA.ML..............EP..DR..Q...............N..N.G...KA.CVLGDTTRILKDLV.SQVESL....
TabHLH416   ..KKAPR.M.....IHKAERE.K.......HKR...........DL......LNDL..FSELDE.ML..............EA..DR..Q...............T..N.G...KA.CILTDTTRILRDLL.SQLESL....
TabHLH417   ..KKAPR.M.....IHKAERE.K.......HKR...........DL......LNDL..FGELGE.ML..............EA..DR..Q...............T..N.G...KA.CILTDTTRILRDLL.SQLESL....
TabHLH418   ..KKAPR.M.....IHKAERE.K.......HKR...........DL......LNDL..FSELGE.ML..............EA..DR..Q...............T..N.G...KA.CILTDTTRILRDLL.SQLESL....
TabHLH419   ..EKAPK.K.....NHKAERE.K.......LKR...........DQ......LNDL..FVELSS.ML..............DH..DR..Q...............N..R.G...KA.TVLGDAARVLRDLT.TQVESL....
TabHLH420   ..EKAPK.K.....NHKAERE.K.......LKR...........DQ......LNDL..FVELSS.ML..............DH..DR..Q...............N..R.G...KA.TVLGDVARVLRDLI.TQVESL....
TabHLH421   ..SVTAR.K.....VHKADRE.R.......MRR...........DK......LNEQ..FLELGT.TL..............DP..DR..P...............R..H.D...KA.TILGDTVQMLKDLT.SQVNKL....
TabHLH422   ..EVESQRM......THIAVE.R.......NRR...........RQ......MNDY..LRVLRS.L...............MPGSYV..Q...............R..G.D...QA.SIIGGAIEFIRELE.QLIQCL....
TabHLH423   ..EVESQRM......THIAVE.R.......NRR...........RQ......MNDY..LRVLRS.L...............MPGSYV..Q...............R..G.D...QA.SIIGGAIEFIRELE.QLIQCL....
TabHLH424   ..EVESQRM......THIAVE.R.......NRR...........RQ......MNDY..LRVLRS.L...............MPGSYV..Q...............R..G.D...QA.SIIGGAIEFIRELE.QLIQCL....
TabHLH425   ..DAESQRM......THIAVE.R.......NRR...........RQ......MNEY..LAVLRS.L...............MPESYA..H...............R..G.D...QA.SIVGGAIDFVKELE.QLLQSL....
TabHLH426   ..DAESQRM......THIAVE.R.......NRR...........RQ......MNEY..LAALRS.L...............MPESYA..H...............R..G.D...QA.SIVGGAIDFVKELE.QLLQSL....
TabHLH427   ..DAESQRM......THIAVE.R.......NRR...........RQ......MNEY..LAALRS.L...............MPESYA..H...............R..G.D...QA.SIVGGAIDFVKELE.QLLQSL....
TabHLH428   ..EVESQRR......THIAVE.R.......NRR...........RQ......MNEY..LAALRS.L...............MPQSYA..Q...............R..G.D...QA.SIVGGAINYVKELE.QLLQSL....
TabHLH429   ..EVESQRR......THIAVE.R.......NRR...........RQ......MNEY..LAALRS.L...............MPQSYA..Q...............R..G.D...QA.SIVGGAINYVKELE.QLLQSL....
TabHLH430   ..EVESQRR......THIAVE.R.......NRR...........RQ......MNEY..LAALRS.L...............MPQSYA..Q...............R..G.D...QA.SIVGGAINYVKELE.QLLQSL....
TabHLH431   ..EVESQRM......THIAVE.R.......NRR...........KQ......MNEY..LAALRS.L...............MPPSYA..Q...............R..G.D...QA.SIVGGAINFVKELE.QLLQTL....
TabHLH432   ..EVESQRM......THIAVE.R.......NRR...........KQ......MNEY..LAALRS.L...............MPPSYA..Q...............R..G.D...QA.SIVGGAINFVKELE.QLLQTL....
TabHLH433   ..EVESQRM......THIAVE.R.......NRR...........KQ......MNEY..LAALRS.L...............MPPSYA..L...............R..G.D...QA.SIVGGAINFVKELE.QLLQTL....
TabHLH434   ..EAESQRM......THIAVE.R.......NRR...........KQ......MNEY..LGVLRS.L...............MPASYV..H...............R..G.D...QA.SIIGGAINYVKELE.QLVQSL....
TabHLH435   ..EAESQRM......THIAVE.R.......NRR...........KQ......MNEY..LGVLRS.L...............MPASYV..H...............R..G.D...QA.SIIGGAINYVKELE.QLVQSL....
TabHLH436   ..EAESQRL......THIAVE.R.......NRR...........RQ......MNEY..LGVLRS.L...............MPASYV..H...............R..G.D...QA.SIIGGAINYVKELE.QLVQSL....
TabHLH437   ..ETECQRM......THIAVE.R.......NRR...........RQ......MNEY..LVVLRS.L...............MPESYV..Q...............R..G.D...QA.SIVGGAIDFVKELE.QQLQSL....
TabHLH438   ..ETECQRM......THIAVE.R.......NRR...........RQ......MNEY..LVVLRS.L...............MPESYV..Q...............R..G.D...QA.SIVGGAIDFVKELE.QQLQSL....
TabHLH439   ..ETECQRM......THIAVE.R.......NRR...........RQ......MNEY..LVVLRS.L...............MPESYV..Q...............R..G.D...QA.SIVGGAIDFVKELE.QQLQSL....
TabHLH440   ..ETESQRR......NHIAVE.R.......NRR...........RQ......MNDY..LAVLRS.S...............MPPSYA..Q...............R..G.D...QA.SIVAGAINFVKELE.QLLQSL....
TabHLH441   ..ETESQRR......NHIAVE.R.......NRR...........RQ......MNDY..LAVLRS.A...............MPPSYA..Q...............R..G.D...QA.SIVAGAINFVKELE.QLLQSL....
TabHLH442   ..ETESQRR......NHIAVE.R.......NRR...........RQ......MNDY..LAVLRS.A...............MPPSYA..Q...............R..G.D...QA.SIVAGAINFVKELE.QLLQSL....
TabHLH443   ..EAECQRM......THIAVE.R.......NRR...........RL......MNDH..LASLRA.L...............IPSDYI..P...............R..G.D...QA.TVVGGAIDYVKQLE.QQLVAL....
TabHLH444   ..EAECQRM......THIAVE.R.......NRR...........RL......MNDH..LASLRA.L...............IPSDYI..P...............R..G.D...QA.TVVGGAIDYVKQLE.QQLVAL....
TabHLH445   ..EAECQRM......THIAVE.R.......NRR...........RL......MNDH..LASLRA.L...............IPSDYI..P...............R..G.D...QA.TVVGGAIDYVKQLE.QQLVAL....
TabHLH446   ........M......SHIAVE.R.......NRR...........RQ......MNDH..LKVLRA.L...............TPAFYI..K...............R..C.D...QA.SIIGGAIEFIRELH.TVLDAL....
TabHLH447   ........M......SHIAVE.R.......NRR...........RQ......MNDH..LKVLRA.L...............TPAFYI..K...............R..C.D...QA.SIIGGAIEFIRELH.TVLDAL....
TabHLH448   ..AEGRRKM......SHIAVE.R.......NRR...........RQ......MNDH..LKVLRA.L...............TPAFYI..K...............R..C.D...QA.SIIGGAIEFIRELH.TVLDAL....
TabHLH449   ..GGAAPKT......AHITVE.R.......NRR...........KQ......MNEH..LTVLRS.L...............MPCFYV..K...............R..G.D...QA.SIIGGVVDYIKELQ.QVKQSL....
TabHLH450   ..GGAAPKT......AHITVE.R.......NRR...........KQ......MNEH..LTVLRS.L...............MPCFYV..K...............R..G.D...QA.SIIGGVVDYIKELQ.QVKQSL....
TabHLH451   ..GGAAPKT......AHITVE.R.......NRR...........KQ......MNEH..LTVLRS.L...............MPCFYV..K...............R..G.D...QA.SIIGGVVDYIKELQ.QVKQSL....
TabHLH452   .PGSHKK.......LSHNAYE.R.......DRR...........KQ......LNEL..YLSLRS.L...............LPDTD.....HTK...........K.......LSIPTTVCRALKYIPELQ.KQVENL....
TabHLH453   .PGSHKK.......LSHNAYE.R.......DRR...........KQ......LNEL..YLSLRS.L...............LPDAD.....HTK...........K.......LSIPTTVCRALKYIPELQ.KQVENL....
TabHLH454   .SGSHRK.......ISHNAYE.R.......DRR...........KE......LNEL..YSDLRS.L...............LPEDD.....RTK...........K.......LSIPITVSRVLKYIPEQQ.KQVEGL....
TabHLH455   .SGSHRK.......ISHNAYE.R.......DRR...........KE......LNEL..YSDLRS.L...............LPEDD.....RTK...........K.......LSIPITVSRVLKYIPELQ.KQVEGL....
TabHLH456   .SGSHRK.......ISHNAYE.R.......DRR...........KE......LNEL..YSDLRS.L...............LPEDD.....RTK...........K.......LSIPITVSRVLKYIPELQ.KQVEGL....
TabHLH457   .SGSHRK.......ISHNAYE.R.......DRR...........KQ......LNEL..YSDLRS.L...............LPDTD.....HTK...........K.......LSIPITVSRVLKYIPELQ.KQVDGL....
TabHLH458   .SGSHRK.......ISHNAYE.R.......DRR...........KQ......LNEL..YSDLRS.L...............LPDTD.....HTK...........K.......LSIPITVSRVLKYIPELQ.KQVDGL....
TabHLH459   .SGSHRK.......ISHNAYE.R.......DRR...........KQ......LNEL..YSDLRS.L...............LPDTD.....HTK...........K.......LSIPITVSRVLKYIPELQ.KQVDGL....
TabHLH460   .SGSQRK.......MSHNAYE.R.......DRR...........KQ......LNEQ..YSSLRS.L...............LPDDD.....HNK...........K.......MSIPTTVSRVIKYIPELQ.KEVDGL....
TabHLH461   .SGSHRK.......MSHNAYE.R.......DRR...........KQ......LNEQ..YSSLRS.L...............LPDDD.....HNK...........K.......MSIPTTVSRVIKYIPELQ.KEVDGL....
TabHLH462   .SGSHRK.......MSHNAYE.R.......DRR...........KQ......LNEQ..YSSLRS.L...............LPDDD.....HTK...........K.......MSIPTTVSRVINYIPELQ.KEVDRL....
TabHLH463   .SGSHKK.......LNHNAYE.R.......DRR...........TQ......LNQL..YSTLRS.L...............IPNAD.....HTK...........K.......LSIPTTVCQVLDYIPKLQ.KQVEDL....
TabHLH464   .SGSHKK.......LNHNAYE.R.......DRR...........TQ......LNQL..YSTLRS.L...............IPNAD.....HTK...........K.......LSIPTTVCQVLDYIPKLQ.KQVEDL....
TabHLH465   .SRSHRK.......MSHSAYE.R.......DSR...........KQ......LNEH..YSNLRS.L...............LPDDD....HHTR...........K.......LGNPTIVSRVIKYIPELQ.KEVDGL....
TabHLH466   .................SRRS.R.......TRR..TGSSPSISEEQ......ISEL..LSKLQA.L...............LPESQA..R...............N..A.HRGSAA.RVLQETCSYIRSLH.REVDDL....
TabHLH467   .................SRRS.R.......TRR..AGSSPSISEEQ......ISEL..LSKLQA.L...............LPESQA..R...............N..A.HRGSAA.RVLQETCNYIRSLH.REVDDL....
TabHLH468   .................SRRS.R.......TRS..AGSSPSISEDQ......ISEL..LSKLQA.L...............LPESQA..R...............N..A.HRGSAA.RVLQETCSYIRSLH.REVDDL....
TabHLH469   .................SRRS.R.......TRH..AGSSTSISEDQ......ISEL..LSKLQA.L...............LPESQA..R...............N..A.HRGSAA.RVLQETCSYIRSLH.REVDNL....
TabHLH470   .................SRRS.R.......TRR..GGSSSSISEEQ......ISEL..LSKLQA.V...............LPESQA..R...............N..A.HRGSAA.RVLEETCSYIRSLH.RELDDL....
TabHLH471   ................SSRRS.R.......SRQ..SGSSR.ITDEQ......ISDL..VSKLQD.L...............LPEARL..R..................N.DRVPSS.RVLQETCTYIRSLH.REVDDL....
TabHLH472   ................SSRRS.R.......SRQ..SGSSR.ITDEQ......ISDL..VSKLQD.L...............LPEARL..R..................N.DRVPSS.RVLQETCTYIRSLH.REVDDL....
TabHLH473   ................RVSLS.R.......SRQ..SGSSR.ITDEQ......ISDL..VSKLQD.L...............LPEARL..R..................N.DRVSSS.RVLQETCTYIRSLH.REVDDL....
TabHLH474   ..................MSS.........RR......GRITDEE......INEL..ISKLQA.L...............VPESSR..R...............R..A.SRSSAS.KLLKETCGYIKSLH.QEVEDL....
TabHLH475   ..................MSS.........RR......GRITDEE......INEL..ISKLQA.L...............LPESSR..R...............R..A.SRSSAS.KLLKETCGYIKSLH.QEVDDL....
TabHLH476   ..................MSS.........RR...SSRGAISEEE......INEL..MSKLQS.L...............LPNS.R..R...............R..S.SQASTT.KLLKETCSYIKSLH.REVDDL....
TabHLH477   ..................MSS.........RR...SSRGAISDEE......VNEL..MSKLQS.L...............LPNS.R..R...............R..S.SQASTT.KLLKETCSYIKSLH.REVDDL....
TabHLH478   ..................MSS.........RR...SSRGSISDEE......VNEL..MSKLQS.L...............LPNS.R..R...............R..S.SQASTT.KLLKETCSYIKSLH.REVDDL....
TabHLH479   ..................MSG.........RR....SRGSVSEEE......INEL..ISRLQT.L...............LPTT.R..R...............R.SS.SQASTT.KMLKETCSYIKSLH.REVDDL....
TabHLH480   ..................MSG.........RR....SRGSVSEEE......INEL..ISRLQT.L...............LPTA.R..R...............R.SS.SQASTT.KMLKETCSYIKSLH.REVDDL....
TabHLH481   ..................MSG.........RR....SRGSVSEEE......INEL..ISRLQT.L...............LPTA.R..R...............R.SS.SQASTT.KMLKETCSYIKSLH.REVDDL....
TabHLH482   VPRSSA.F.......RHITRE.R.......LRR...........ER......LSQG..YADIRA.L...............LPP.GR..AP..............N..G.A...KN.FVLAAAVDYIRELE.GRKGRL....
TabHLH483   VPRSSA.F.......RHITRE.R.......LRR...........ER......LSQG..YADIRA.L...............LPP.GR..AP..............N..G.A...KN.FVLAAAVDYIRELE.GRKGRL....
TabHLH484   VPRSSA.F.......RHITRE.R.......LRR...........ER......LSQG..YADIHA.L...............LPP.GR..AS..............N..G.A...KN.CVLAAAVDYIRELE.GRKGRL....
TabHLH485   .VESSRGF.......RHMMRE.R.......QRR...........EK......LSQS..YADLHA.M...............V.....SS..RS..............K..Q.D...KN.SIVQSAAVYIHELK.VAKEQL....
TabHLH486   .VESSRGF.......RHMMRE.R.......QRR...........EK......LSQS..YADLYA.M...............V.....SS..RS..............K..Q.D...KN.SIVQSAAVYIHELK.VAKEQL....
TabHLH487   .VESSRGF.......RHMMRE.R.......QRR...........EK......LSQS..YADLYA.M...............V.....SS..RS..............K..Q.D...KN.SIVQSAAVYIHELK.VAKEQL....
TabHLH488   ....SAA........ARAALV.P.......P.............APPARQAGEPAR..SDALRR.L...............VP..GG..A...............GM...E...YC.SLLEETADYVRCLR.AQVQLM....
TabHLH489   ....AAA........ARAALA.P.......P.............PPPARQAGEPAR..SDALRR.L...............VP..GG..A...............GM...E...YC.SLLEETADYVRCLR.AQVQLM....
TabHLH490   ....SAA........ARAALV.P.......P.............PPPARQAGEPAR..SDALRR.L...............VP..GG..A...............GM...E...YS.SLLEETADYVRCLR.AQVQLM....
TabHLH491   KA.GTAAR....PRGSAGVVA.R.......AM.......................LRKR..TQVLKG.I...............VP..GV..Q...............AV...D...EC.TLLGEALDYAVCLK.AQVDVM....
TabHLH492   KA.GTAAR....PRGSAGIVA.R.......AM.......................VRKR..TQVLKG.I...............VP..GV..E...............GV...D...EC.TLLGEALDYAVCLK.AQVDVM....
TabHLH493   KA.GTAAR....PWGSAGVVA.R.......AM.......................VRKR..TQVLKG.I...............VP..GV..E...............AV...D...EC.TLLGEALDYAVCLK.AQVDVM....
TabHLH494   .RRTTA.........SSGEIA.R.......RL.......................VRKR..TKVLRG.M...............VP..GG..E...............LL...D...GA.SLLREAVDYVVHLR.AQVAVL....
TabHLH495   .RRTTT.........SSGEIA.R.......RL.......................VRKR..TKVLRG.M...............VP..GG..E...............LL...D...GA.SLLREAMDYVVHLR.AQVAVL....
TabHLH496   .RRTTT.........SSGEIA.R.......RL.......................VRKR..TKVLRG.M...............VP..GG..E...............LL...D...GA.SLLREAMDYVVHLR.AQVAVL....
TabHLH497   .RSGRT.........GGGDTP.G.......RL.......................VSSR..TMALRE.V...............IP..GG..R...............NS...D...EA.TLLREAMDYAMHLR.AQVDVL....
TabHLH498   .RSGRA.........GGGDVA.G.......RL.......................LKSR..TMALRD.V...............IP..GG..R...............NS...D...EA.TLLREAMDYAVHLR.AQVDVL....
TabHLH499   KRS...............NVV.G.......RL.......................VRRR..MTALRE.V...............IP..GG..R...............DS...D...EV.TLLCEAMDYAVHLR.AQVDVL....
TabHLH500   KRS...............YVA.G.......RL.......................LRRW..TIALRD.V...............IP..GG..R...............HA...D...EV.TLLREAMDYAVHLR.AQVDVL....
TabHLH501   KRS...............YVA.G.......RL.......................LRRR..TMALRE.V...............IP..GG..R...............DA...D...EA.TLLREAMDYAVHLR.AQVDVL....
TabHLH502   ...............................................................MALRD.V...............IP..GG..R...............HS...D...EV.ILLREAMDYAVHLC.AQVDVL....
TabHLH503   .RSGRA.........GGGAVA.R.......RL.......................LRTR..TMALRE.L...............IP..GG..R...............DA...D...EV.TLLREAMDYTVHLR.AQVDVL....
TabHLH504   .RSGRA.........GAGAVA.R.......RL.......................LRTR..TMALRD.V...............IP..GG..R...............DA...D...QA.TLLREAMDYAVHLR.AQVDVL....
TabHLH505   ..............................RR.......................IVRR..TMALRE.V...............IP..GG..R...............DS...D...EA.TLLREAVDYTVHLR.AQVDVL....
TabHLH506   LPNTPKS........KHSATE.Q.......RRR...........SK......INDR..FQILRD.L...............LP..HT..DQ..............K..R.D...KA.TFLLEVIEYIRFLQ.EKAQKY....
TabHLH507   LPNTPKS........KHSATE.Q.......RRR...........SK......INDR..FQILRD.L...............LP..HT..DQ..............K..R.D...KA.TFLLEVIEYIRFLQ.EKAQKY....
TabHLH508   LPNTPKS........KHSATE.Q.......RRR...........SK......INDR..FQILRD.L...............LP..HT..DQ..............K..R.D...KA.TFLLEVIEYIRFLQ.EKAQKY....
TabHLH509   APGSPRS........KHSATE.Q.......RRR...........CK......INER..FQILRD.L...............VP..QS..DQ..............K..R.D...KA.TFLLEVIEYVKFLQ.EKVQNH....
TabHLH510   APGSPRS........KHSATE.Q.......RRR...........CK......INER..FQILRD.L...............VP..QS..DQ..............K..R.D...KA.TFLLEVIEYVKFLQ.EKVQKH....
TabHLH511   APGSPRS........KHSATE.Q.......RRR...........CK......INER..FQILRD.L...............VP..QS..DQ..............K..R.D...KA.TFLLEVIEYVKFLQ.EKVQKH....
TabHLH512   DPSSPRS........KHSATE.Q.......RRR...........TK......INDR..LDVLRD.L...............LP..NC..DQ..............K..R.D...KA.SFLLQVIEYIRLLQ.EKCQKY....
TabHLH513   DPSSPRS........KHSATE.Q.......RRR...........TK......INDR..LDILRD.L...............LP..NC..DQ..............K..R.D...KA.SFLLEVIEYIRLLQ.EKCQKY....
TabHLH514   DPSSPRS........KHSATE.Q.......RRR...........TK......INDR..LDILRD.L...............LP..NC..DQ..............K..R.D...KA.SFLLEVIEYIRLLQ.EKCQKY....
TabHLH515   ...................MS.R.......ERK...........KA......AAAL..QEKMQI.L...............RSI.....T...............H..S.HALSNP.SIVMDASEYIKGLK.QKIARL....
TabHLH516   ...................MS.R.......ERK...........K.......AAAL..QEKMQI.L...............RSI.....T...............H..S.HALSNP.SIVMDASEYIKGLK.QKIARL....
TabHLH517   ...................MS.R.......ERK...........K.......AAAL..QEKMQI.L...............RSI.....T...............H..S.HALSNP.SIVMDASEYIKGLK.QKIARL....
TabHLH518   ..................MMS.R.......ERK...........K.......AAAL..QEKLQL.L...............RSL.....T...............H..S.HALSNT.SIIMDASKYIKELK.QKVVML....
TabHLH519   ..................MMS.R.......ERK...........K.......AAAL..QEKLQL.L...............RSL.....T...............H..S.HALSNT.SIIMDASKYIKELK.QKVVML....
TabHLH520   ..................MMS.R.......ERK...........K.......AAAL..QEKLQL.L...............RSL.....T...............H..S.HALSNT.SIIMDASKYIKELK.QKVVML....
TabHLH521   .RSGRKA......TRT..TAG.R.......RR..............CVE.AIRRK..MEALRR.L...............VP..SG..G...............DH.......VD.ELLFRAADYIVRLQ.VQVKAM....
TabHLH522   .RSGKKA......TRT..KAE.R.......RR..............CME.AIRRK..MEALRR.L...............VP..SG..G...............DH.......VD.ELLFRAADYIVRLQ.VQVKAM....
TabHLH523   ....GKS......KGVVASLM.H.......RR..............PFQHMVLRR..LRELKK.I...............VP..DA.................A.D...VD.VLLRQTAEYICILE.LKVAAL....
TabHLH524   ....GKS......KGVVASLM.H.......RR..............PFQHMVLRR..LRELKK.I...............VP..DA.................A.D...VD.VLLRQTAEYICILE.LKVAAL....
TabHLH525   ....GKS......KGVVASLM.H.......RR..............PFQHMVLRR..LRELKK.I...............VP..DA.................A.D...VD.VLLRQTAEYICILE.LKVAAL....
TabHLH526   ..EIARR......RRSLCSSR.R.......RR..............SAA..VGRK..VRELRR.L...............VP..GA..A...............VM.......TD.RLLVRTADYIAQLR.VRVELL....
TabHLH527   HEGGKKELH.............RP......RPR...........DR....QLIQDR..MKELRE.L...............IP..NA..S...............K..I.D......ALLDKTITQMLFLQ.SVSEKA....
TabHLH528   HEGGKKELH.............RP......RPR...........DR....QLIQDR..MKELRE.L...............IP..NA..S...............K..I.D......ALLDKTITQMLFLQ.SVSEKA....
TabHLH529   HEGGKKELH.............RP......RPR...........DR....QLIQDR..MKELRE.L...............IP..NA..S...............K..I.D......ALLDKTITQMLFLQ.SVSEKA....
TabHLH530   KRSRPGESS.............KA......RPK...........DR....QLIQDR..IKELRE.M...............VP..NG..A...............K..I.D......ALLEKTVKHMLFLQ.SVTKHA....
TabHLH531   KRSRPGESS.............KA......RPK...........DR....QLIQDR..IKELRE.M...............VP..NG..A...............K..I.D......ALLEKTVKHMLFLQ.SVTKHA....
TabHLH532   KRSRPGESS.............KA......RPK...........DR....QLIQDR..IKELRE.M...............VP..NG..A...............K..I.D......ALLEKTVKHMLFLQ.SVTKHA....
TabHLH533   KRSRPGESS.............KP......RPK...........DR....QLIQDR..IKELRE.L...............VP..NG..A...............K..I.D......ALLEKTIKHMVFLQ.SVTKHA....
TabHLH534   KRSRPGESS.............KP......RPK...........DR....QLIQDR..IKELRE.L...............VP..NG..A...............K..I.D......ALLEKTIKHMVFLQ.SVTKHA....
TabHLH535   KRSRPGESS.............KP......RPK...........DR....QLIQDR..IKELRE.L...............VP..NG..A...............K..I.D......ALLEKTIKHMVFLQ.SVTKHA....
TabHLH536   KRARPGEST.............RP......RPK...........DR....QQIQDR..VKELRE.I...............VP..NS..A...............K..I.D......ALLDRTIKHMIFLQ.GVTKYA....
TabHLH537   KRARPGEST.............RP......RPK...........DR....QQIQDR..VKELRE.I...............VP..NS..A...............K..I.D......ALLDRTIKHMIFLQ.GVTKYA....
TabHLH538   KRARPGEST.............RP......RPK...........DR....QQIQDR..VKELRE.I...............VP..NS..A...............K..I.D......ALLDRTIKHMIFLQ.GVTKYA....
```

```
TabHLH539  ...GSSR.......SKEAGEK.R.....FLED...........SR.....VLVKQ..VRELRR.L...............VP..CC..H.............E..L.G......ELFQDAASHIEDLQ.VQVKVM....
TabHLH540  ...GSSR.......SKEGGEK.H.....FLED...........SR.....VLVKQ..VRELRR.L...............VP..CC..R.............E..L.G......ELFQDAATHIEDLQ.VQVKVM....
TabHLH541  .AGASRR.......RRSPRLE.R......ARR...........QT......MSVL..FDELGA.L...............LP..DL..P............HR..A.C...RA.DVVDGAIAYVRALE.DTAAEL....
TabHLH542  .AGASRR.......RRSPRLE.R......ARR...........QA......MSVL..FDELGA.L...............LP..DL..P............HR..A.C...KA.DIVDGAIAYVRALE.DTAAEL....
TabHLH543  .SGASRR.......RRSPRLE.R......ARR...........QT......MSVL..FDELGA.L...............LP..DL..P............PR..A.C...RA.DVVDGAIAYVRALE.DTAAEL....
TabHLH544  VAGSTRD.......PGRPQRR.R......AQF...........P..........L..YAELAA.L...............LP..GD..L............SR..A.N...QV.EILDAAVAHLKVLS.GTAAVL....
TabHLH545  VSGSTRA.......PGRPQRR.R......PQF...........P..........L..YAELGA.L...............LP..GD..L............SR..A.N...QV.EILDAAVAHLKVLA.DTAAVL....
TabHLH546  .ADGGCK.......MERKDVE.K......NRR...........LH......MKGL..CLKLSS.L...............VPASSSSDH.............HLNK.DAATQL.DQLDSAAAYIKQLR.GRIDDL....
TabHLH547  .HSSGSK.......MERKDVE.K......NRR...........LH......MKGL..CLKLSS.L...............VPASPSSDH.............HLNK.DAATQL.DQLDSAAAYIKQLR.GRIDDL....
TabHLH548  .TTSSGK.......MERKDVE.K......NRR...........LH......MKGL..CLKLSS.L...............VPASST..H.............HLNK.DAATQL.DQLDSAAAYIKQLR.ARIEDL....
TabHLH549  .GGCSGK.......MERKDVE.K......NRR...........LH......MKGL..CLKLSS.L...............LPASSA..H.............HLNK.DAATQL.DQLDSAAAYIKQLR.GRIDDL....
TabHLH550  .GCSASK.......MERKDVE.K......NRR...........LH......MKGL..CLKLSS.L...............VPPSSA..H.............HLNK.DAATQL.DQLDSAAAYIKQLR.ARIDHL....
TabHLH551  .PTEKLL.......MEKKDSE.K......ERR...........QR......MKAL..CDKLAS.L...............IPK.....E.............D....DTVTQL.GSLDVAASYIKKLK.ERVDEL....
TabHLH552  .PTAKVL.......MEKKESE.K......ERR...........QH......MKRL..CEKLSS.L...............IPK.....E.............D....DTMTQL.GSLDVAASYIKKLK.ERVDEL....
TabHLH553  .AAAAVL.......MEKKDSE.K......ERR...........QR......MKGL..CDKLAS.L...............IPK.....E.............H....DTMTQL.GSLDVAASYIKMLK.ERVDEL....
TabHLH554  .AAAMIL.......VEKKDSE.K......ERR...........QR......MKAL..CDKLAS.L...............IPK.....E.............H....DTMTQL.GSLDVAASYIKKLK.ERVDEL....
TabHLH555  .GGMAVP.......VEKKVLE.R......ERR...........QR......MKAL..CEKLGS.L...............IPK.....E.............H....DTMTQL.GSLDVAASYIKKLT.EWVDEL....
TabHLH556  .GSRAVP.......VEKKASE.K......EKR...........QR......MKAL..CGKLAS.L...............ISK.....E.............H....DTMTQL.GSLDVAASYIKKLK.EKVDEL....
TabHLH557  .GDTAAP.......MEKKRSE.R......QRR...........QR......MKAL..CDKLAS.L...............IPK.....Q.............H.........L.GSLDVAASYIKKLK.ERVDDL....
TabHLH558  .RGTAVL.......VEKKRSE.R......ERR...........QR......MKAL..CEKLAS.L...............IPK.....E.............H.........T.G..DVAASYIKKLK.ERVDEL....
TabHLH559  .GTAVVL.......LEKKESE.K......ERR...........KR......MKGL..CEKLAS.L...............IPR.....E.............HCDK.DTMTQL.GSLDVGASYIKKLK.ERVDEL....
TabHLH560  .GMAVVL.......LEKKESE.K......ERR...........KR......MKAL..CEKLAS.L...............IPR.....E.............HC...DTMTQL.GSLDVGASYIKKLK.ERVDEL....
TabHLH561  .GTAVVL.......LEKKESE.K......ERR...........KR......MKAL..CEKLAS.L...............IPR.....E.............HC...DTMTQL.GSLDVGASYIKKLK.ERVDEL....
TabHLH562  .GTTVVL.......LEKKESE.K......ERR...........KR......MKAL..CEKLAS.L...............IPR.....E.............HC...DTMTQL.GSLDVGASYIKKLK.ERVDEL....
TabHLH563  .GTAGVL.......LEKKESE.K......ERR...........KR......MKAL..CEKLAS.L...............IPR.....E.............HC...DTMTQL.GSLDAGASYIKKLK.ERIDEL....
TabHLH564  .GTTARV.......VPRKEAE.R......ERR...........QH......MKAL..CAKLAS.L...............IPK.....E.............H....STVTQL.GSLDEAVSYIKKLK.ERVDEL....
TabHLH565  .TTATRV.......VQRKEAE.R......ERR...........QH......MKAL..CAKLTS.L...............IPK.....E.............H....DTMTQL.SSLDEAASYIKKLK.ERVDEL....
TabHLH566  .TTTTRV.......VERKEAE.R......ERR...........QH......MKAL..CAKLTS.L...............IPK.....E.............H....DTMTQL.GSLDEAASYIKKLK.ERVDEL....
TabHLH567  .TTAARV.......VERKEAE.R......ERR...........QH......MKAL..CAKLAS.L...............IPK.....E.............H....DTMTQL.GSLDESASYIKKLK.ERVDEL....
TabHLH568  .TTAARV.......VERKEAE.R......ERR...........QH......MKAL..CAKLAS.L...............IPK.....E.............H....DTMTQL.GSLDEAVSYIKKLK.EKVDEL....
TabHLH569  .TTAARV.......VERKEAE.R......ERR...........QH......MKAL..CAKLTS.L...............IPK.....E.............Y....DTMTQL.GSLDEAASYIKKLK.ERVDEL....
TabHLH570  .GGTAVP.......VEKKELE.R......ERR...........QR......MKAL..CEKLAS.C...............IPK.....E.............Y....DAATQL.DQLDSATAYIKQLK.ARIDHL....
TabHLH571  .ATAGGK.......PERKTVE.R......NRR...........NQ......MNAL..YYRLDT.L...............VRAGGA...................P.AAATRP.DRLGEAAAYISQTA.ERVERL....
ZmbHLH001  ..EAESQRM......THIAVE.R......NRR...........RL......MNDH..LASLRS.L...............IPSSYT..P.............R..G.D...QA.TVVGGAIDYVKQLE.QQLVAL....
ZmbHLH002  ..EIESQRI......THIAVE.R......NRR...........RQ......MNEY..LAVLRS.L...............MPPSHA..H.............R..G.D...QA.SIVGGAINYVRELE.QLLQSL....
ZmbHLH003  ..EIESQRM......THIAVE.R......NRR...........RQ......MNEY..LAVLRS.L...............MPPSYA..H.............R..G.D...QA.SIVGGAINYVRELE.QLLQSL....
ZmbHLH004  ..EVESQRM......IHIAVE.R......NRR...........KQ......MNEH..LAALRS.L...............MPPAHT..Q.............R..G.D...QA.SIVGGAINFVKELE.QLLQSL....
ZmbHLH005  ..EVESQRM......VHIAVE.R......NRR...........KQ......MNEY..LAALRS.L...............MPPAYT..Q.............R..G.D...QA.SIVGGAINFVKELE.QLLQSL....
ZmbHLH006  ..EVESQRR......NHIAVE.R......NRR...........RQ......MNEY..LSVLRS.A...............LPPSYP..Q.............R..G.D...QA.SIVAGAINFVKELE.HLLQSL....
ZmbHLH007  ..EMESQRR......NHIAVE.R......NRR...........RQ......MNEY..LSVLRS.A...............MPPSYT..Q.............R..G.D...QA.SIVAGAINFVKELE.QLLQSL....
ZmbHLH008  ..EAETQRM......THIAVE.R......NRR...........RQ......MNEY..LAALRS.T...............MPEAYV..Q.............R..G.D...QA.SIVGGAIVFVRELE.QQLQCL....
ZmbHLH009  ..ETETQRM......THIAVE.R......NRR...........RQ......MNEY..LAALRS.I...............MPEAYV..Q.............R..G.D...QA.SIVGGAIEFVKELE.QQVQCL....
ZmbHLH010  ..EVESQRM......THIAVE.R......NRR...........RQ......MNEY..LRILRS.L...............MPGSYV..Q.............R..G.D...QA.SIIGGAIEFIRELE.QLIQCL....
ZmbHLH011  ..DAESQRM......THIAVE.R......NRR...........RQ......MNEY..LAALRS.L...............MPDSYV..H.............R..S.D...QA.SVVSGAIDFVKELE.QQLQSL....
ZmbHLH012  ..DEGAAKM......SHITVE.R......NRR...........KQ......MNEH..LAVLRS.L...............MPCFYV..K.............R..G.D...QA.SIIGGVVDYIKELQ.QVLRSL....
ZmbHLH013  ..DEGAPKI......SHVAVE.R......NRR...........KQ......MNEH..LTVLRS.L...............MPCFYV..K.............R..G.D...QA.SIIGGVVDYIKELQ.QVLRSL....
ZmbHLH014  ..DGAVNKT......SHITVE.R......NRR...........KQ......MNEH..IAVLRS.L...............MPCFYV..K.............R..G.D...QA.SIIGGVVDYIKELQ.QVLQSL....
ZmbHLH015  .......M.......SHIAVE.R......NRR...........RQ......MNEH..LKVLRS.L...............TPGLYI..K.............R..G.D...QA.SIIGGAIEFIKELQ.QVLESL....
ZmbHLH016  ..EAESQRM......THIAVE.R......NRR...........RL......MNDH..LASLRS.L...............IPSSYI..P.............R..V.SHAMQS.PCMHAAFRF.DLTT.QRLHAMQCRA
ZmbHLH017  ...AGGA.....CQSVQNSAA.R......QRR...........KR......ISEK..TAELAR.L...............IP..GA..N.............KL.......TA.EMLEEASRHVKLLQ.AQVGML....
ZmbHLH018  KRGQATN........SHSLAE.R......FRR...........EK......INER..MKLLQD.L...............VP..GC..N.............KI.T.G...KA.MMLDEIINYVQSLQ.RQIEFL....
ZmbHLH019  KRGQATN........SHSLAE.R......FRR...........EK......INVR..MKLLQD.L...............VP..GC..N.............KI.T.G...KA.MMLDEIINYVQSLQ.RQVEFL....
ZmbHLH020  RRGQATD........SHSLAE.R......VRR...........ER......ISQR..MKVLQD.L...............VP..GC..N.............KV.I.G...KA.LMLDEIINYVQSLQ.RQVEFL....
ZmbHLH021  RRGEATD........SHSLAE.R......VRR...........EK......ISQR..MKLLQD.L...............VP..GC..N.............KV.V.G...KA.VMLDEIINYVQSLQ.RQVEFL....
ZmbHLH022  RRGEATD........SHSLAE.R......VRR...........EK......ISQR..MKLLQD.L...............VP..GC..N.............KV.V.G...KA.VMLDEIINYVQSLQ.RQVEFL....
ZmbHLH023  RRGQATD........SHSLAE.R......VRR...........EK......ISQR..MKFLQD.L...............VP..GC..N.............KV.V.G...KA.LMLDEIINYVQSLQ.QQVEFL....
ZmbHLH024  RRGQATD........SHSLAE.R......VRR...........EK......ISQR..MKFLQD.L...............VP..GC..N.............KV.V.G...KA.LMLDEIINYVQSLQ.QQVEFL....
ZmbHLH025  KRGQATN........SHSLAE.R......LRR...........KK......ISER..MKLLQD.L...............VP..GC..S.............KI.T.G...KA.VMLDEIINYVQSLQ.RQVEFL....
ZmbHLH026  KRGQATN........SHSLAE.R......LRR...........KK......ISER..MKLLQD.L...............VP..GC..S.............KI.T.G...KA.VMLDEIINYVQSLQ.RQVEFL....
ZmbHLH027  RRGQATN........SHSLAE.R......LRR...........EK......ISER..MKLLQD.L...............VP..GC..S.............KV.T.G...KA.VMLDEIINYVQSLQ.RQVEFL....
ZmbHLH028  RRGQATN........SHSLAE.R......LRR...........EK......ISER..MKLLQD.L...............VP..GC..S.............KV.T.G...KA.VMLDEIINYVQSLQ.RQVEFL....
ZmbHLH029  RRGQATD........SHSLAE.R......VRR...........EK......IGER..MKLLQD.L...............VP..GC..S.............KV.T.G...KA.VMLDEIINYVQSLQ.RQVEFL....
ZmbHLH030  RRGQATD........SHSLAE.R......VRR...........EK......ISER..MKLLQD.L...............VP..GC..S.............KV.T.G...KA.VMLDEIINYVQSLQ.CQVEFL....
ZmbHLH031  RRGQATD........SHSLAE.R......ARR...........EK......ISER..MKVLQD.L...............VP..GC..N.............KV.I.G...KA.SVLDEIINYIQSLQ.CQVEFL....
ZmbHLH032  RRGQATD........SHSLAE.R......ARR...........EK......ISER..MKVLQD.I...............VP..GC..N.............KV.I.G...KA.SVLDEIINYIQSLQ.RQVEFL....
ZmbHLH033  RRGQATD........SHSLAE.R......VRR...........ER......ISER..MRVLQA.L...............VP..GC..D.............KV.T.G...KA.LVLDEIINYVQSLQ.NQVEFL....
ZmbHLH034  RRGQATD........SHSLAE.R......VRR...........ER......ISER..MRMLQA.L...............VP..GC..D.............KV.T.G...KA.MVLDEIINYVQSLQ.NQVEFL....
ZmbHLH035  RRGQATD........SHSLAE.R......VRR...........ER......ISER..MRMLQA.L...............VP..GC..D.............KV.T.G...KA.LILDEIINYVQSLQ.NQVEFL....
ZmbHLH036  RRGQATD........SHSLAE.R......VRR...........ER......ISER..MRYLQE.L...............VP..GC..S.............KV.T.G...KA.GMLDEIINYVQSLQ.KQVEFL....
ZmbHLH037  RRGQATD........SHSLAE.R......VRR...........ER......ISER..MRYLQE.L...............VP..GC..S.............KV.T.G...KA.GMLDEIINYVQSLQ.KQVEFL....
ZmbHLH038  RRGQATD........SHSLAE.R......ARR...........EK......INAR..MELLKE.L...............VP..GC..S.............KV.S.G...TA.LVLDEIINHVQSLQ.RQVEYL....
ZmbHLH039  RRGQATD........SHSLAE.R......ARR...........EK......INAR..MELLKE.L...............VP..GC..S.............KV.S.G...TA.LVLDEIINHVQSLQ.RQVEYL....
ZmbHLH040  RRGQATD........PHSIAE.R......LRR...........ER......IAER..MKALQE.L...............VP..NA..N.............K..T.D...KA.SMLDEIVDYVKFLQ.LQVKVL....
ZmbHLH041  KRGQATD........PHSIAE.R......LRR...........ER......IAER..MKSLQE.L...............VP..NA..N.............K..T.D...KA.SMLDEIIDYVRFLQ.LQVKVL....
ZmbHLH042  RRGQATD........PHSIAE.R......LRR...........ER......IAER..MRALQE.L...............VP..NT..N.............K..T.D...RA.AMLDEILDYVKFLR.LQVKVL....
ZmbHLH043  RRGQATD........PHSIAE.R......LRR...........ER......IAER..MRALQE.L...............VP..NT..N.............K..T.D...RA.AMLDEILDYVKFLR.LQVKVL....
ZmbHLH044  RRGQATD........PHSIAE.R......LRR...........ER......IAER..MKALQE.L...............VS..NA..N.............K..T.D...KA.SMLDEIIDYVKFLQ.LQVLSM....
ZmbHLH045  RRGQATD........PHSIAE.R......LRR...........EK......ISDR..MKNLQE.L...............VP..NS..N.............R..T.D...KA.SMLDEIIEYVKFLQ.LQVKVL....
ZmbHLH046  RRGQATD........PHSIAE.R......LRR...........EK......ISDR..MKNLQD.L...............VP..NS..N.............K..A.D...KA.SMLDEIIDYVKFLQ.LQVKVL....
ZmbHLH047  RRGQATD........PHSIAE.R......LRR...........EK......ISDR..MKNLQD.L...............VP..NS..N.............K..A.D...KA.SMLDEIIDHVKFLQ.LQVKVL....
ZmbHLH048  AAAVHQP........PFG..........QLRR...........ER......IAER..MKALQE.L...............VP..NA..N.............K..T.D...KA.SMLDEIIDYVKFLQ.LQVKVL....
ZmbHLH049  KRRRAAQ........VHNLSE.R......RRR...........DR......INEK..MKALQE.L...............IP..HC..N.............K..A.D...KA.SMLDEAIEYLKSLQ.LQLQVV....
ZmbHLH050  RRSRAAE........VHNLSE.R......RRR...........DR......INEK..MRALQE.L...............IP..HC..N.............K..T.D...KA.SMLDEAIEYLKSLQ.LQVQMM....
ZmbHLH051  RRTRAAE........VHNLSE.R......RRR...........DR......INEK..MRALQE.L...............IP..HC..N.............K..T.D...KA.SILDETIEYLKSLQ.MQVQIM....
ZmbHLH052  KRSRTAE........VHNLSE.R......RRR...........DR......INEK..MRALQE.L...............IP..NC..N.............KI...D...KA.SMLDEAIEYLKTLQ.LQVQMM....
ZmbHLH053  KRSRTAE........VHNLSE.R......RRR...........DR......INEK..MRALQE.L...............IP..NC..N.............KI...D...KA.SMLDEAIEYLKTLQ.LQVQMM....
ZmbHLH054  KRCRTAE........VHNLSE.R......RRR...........DR......INEK..MRALQE.L...............IP..NC..N.............KV...D...KS.SMLEEAIEYLKTLQ.LQVQMM....
ZmbHLH055  KRSRAAE........VHNLSE.K......RRR...........SR......INEK..MKALQT.L...............IP..NS..S.............K..T.D...KA.SMLDDAIEYLKHLQ.LQVQML....
ZmbHLH056  KRSRAAE........VHNLSE.K......RRR...........SK......INEK..MKALQS.L...............IP..NS..N.............K..T.D...KA.SMLDEAIEYLKQLQ.LQVQML....
ZmbHLH057  RRSRSAE........FHNFSE.R......RRR...........DK......INEK..LKALQE.L...............LP..NC..N.............K..T.D...KV.SMLDEAIDYLKSLQ.LQLQML....
ZmbHLH058  RRSRSAD........FHNFSE.R......RRR...........DR......INEK..LRALQE.L...............LP..NC..T.............K..T.D...KV.SMLDEAIDYLKSLQ.LQLQML....
ZmbHLH059  RKGMCRD........ESDSRS.E......RRR...........DR......IKEK..MRALQE.L...............IP..HC..N.............K..T.D...KA.SILDETIEYLKSLQ.MQVQIM....
ZmbHLH060  RRSHHGE........AHNLTE.K......RRR...........HK......INER..FKTLQQ.I...............VP..GC..S.............K..S.N...QA.STLDQTIHYMKSLQ.HQVQAM....
ZmbHLH061  RRSHHGE........AHNLTE.K......RRR...........HK......INER..LKTLQQ.I...............VP..GC..S.............K..S.N...QA.STLDQTIHYMKSLQ.HQVQAM....
ZmbHLH062  NARISTD........PQSVAA.R......VRR...........ER......ISER..IRVLQR.L...............VP..GG..T.............K..M.D...TA.SMLDEAIHYVKFLK.TQVQSL....
ZmbHLH063  NVRISTD........PQSVAA.R......MRR...........ER......ISER..IRILQR.L...............VP..GG..T.............K..M.D...TA.SMLDEAIHYVKFLK.TQVQSL....
ZmbHLH064  NVRVSKD........PQSVAA.R......LRR...........ER......ISER..IRALQR.L...............VP..GG..T.............K..M.D...TA.SMLDEAIQYVKFLK.SQLQSL....
ZmbHLH065  NVRISKD........PQSVAA.R......LRR...........ER......ISER..IRTLQR.L...............VP..GG..T.............K..M.D...TA.SMLDEAIHYVKFLK.SQVQSL....
ZmbHLH066  NVRISED........PQSVAA.R......HRR...........ER......ISER..VRVLQR.L...............VP..GG..T.............K..M.D...TA.SMLDEAIRYIKFLK.RQVQEL....
ZmbHLH067  NVRISED........PQSVAA.R......HRR...........ER......ISER..VRVLQR.L...............VP..GG..T.............K..M.D...TA.SMLDEAIRYIKFLK.RQVQEL....
ZmbHLH068  NVRISED........PQSVAA.R......HRR...........ER......ISER..VRVLQR.L...............VP..GG..T.............K..M.D...TA.SMLDEAIRYIKFLK.RQVQEL....
ZmbHLH069  NVRISED........PQSVVA.R......HRR...........ER......ISER..VRVLQR.L...............VP..GG..T.............K..M.D...TA.SMLDEAIRYIKFLK.RQVQEL....
ZmbHLH070  NVRISSD........PQTVAA.R......LRR...........ER......VSER..LRVLQK.L...............VP..GG..S.............K..M.D...TA.SMLDEAASYLKFLK.SQVQAL....
ZmbHLH071  NVRISSD........PQTVAA.R......LRR...........ER......VSER..LRVLQR.L...............VP..GG..S.............R..M.D...TA.SMLDEAAGYLKFLK.SQVKAL....
ZmbHLH072  NVRISSD........PQTVAA.R......QRR...........ER......ISDR..LRVLQK.L...............VP..GG..A.............K..M.D...TA.SMLDEAASYLRFLK.SQVRDL....
ZmbHLH073  NVRISSD........PQTVAA.R......QRR...........ER......ISER..LRVLQK.L...............VP..GG..A.............K..M.D...TA.SMLDEAASYLRFLQ.SQVREL....
ZmbHLH074  GAKLSTD........PQSVAA.R......ERR...........HR......ISDR..FRVLRS.L...............VP..GG..S.............K..M.D...TV.SMLEQAIHYVKFLK.TQISLH....
ZmbHLH075  SRGAATE........PQSLYA.R......KRR...........ER......INER..LRILQN.L...............VP..NG..T.............K..V.D...IS.TMLEEAAQYVKFLQ.LQIKLL....
ZmbHLH076  SRGGATD........PQSLYA.R......KRR...........ER......INER..LRILQN.L...............VP..NG..T.............K..V.D...IS.TMLEEAAQYVKFLQ.LQIKLL....
ZmbHLH077  ATTTTAE........PQSIYA.R......KRR...........ER......INER..LKVLQS.L...............VP..NG..T.............K..V.D...MS.TMLEEAVHYVKFLQ.LQIRLL....
ZmbHLH078  ATTTTAE........PQSIYA.R......KRR...........ER......INER..LKVLQS.L...............VP..NG..T.............K..V.D...MS.TMLEEAVHYVKFLQ.LQIRLI....
ZmbHLH079  PSRSSKD........SQSLYA.K......RRR...........ER......INEK..LRTLQQ.L...............IP..NG..T.............K..V.D...MS.TMLEEAVQYVKFLQ.LQIKLL....
ZmbHLH080  STAPTKD........PQSLAA.K......NRR...........ER......ISER..LRALQE.L...............VP..NG..T.............K..V.D...LV.TMLEKAISYVKFLQ.LQVKVL....
ZmbHLH081  APAPTKD........PQSVAA.K......VRR...........EK......IAEK..LKVLQD.L...............VP..NG..T.............K..V.D...LV.TMLEKAITYVKFLQ.LQVKVL....
ZmbHLH082  KRGCATH........PRSIAE.R......VRR...........TK......ISER..IRKLQE.L...............VP..DM..D.............K.QT.N...TS.DMLDLAVDYIKDLQ.KQVKAL....
ZmbHLH083  KRGCATH........PRSIAE.R......VRR...........TK......ISER..IRKLQE.L...............VP..NM..E.............K.QT.N...TA.DMLDLAVDYIKDLQ.KQVKVL....
ZmbHLH084  KRGCATH........PRSIAE.R......VRR...........TK......ISER..IRKLQE.L...............VP..NM..E.............K.QT.N...TA.DMLDLAVDYIKDLQ.KQVKVL....
ZmbHLH085  KRGCATH........PRSIAE.R......VRR...........TK......ISER..IRKLQE.L...............VP..NM..D.............K.QT.N...TS.DMLDLAVDYIKELQ.KQVKVL....
ZmbHLH086  KRGCATH........PRSIAE.R......VRR...........TK......ISER..IRKLQE.L...............VP..NM..D.............K.QT.N...TS.DMLDLAVDYIKDLQ.KQVKVL....
ZmbHLH087  RRGCATH........PRSIAE.R......ERR...........TR......ISKR..LKKLQD.L...............VP..NM..D.............K.QT.N...TS.DMLDLAVEYIKELK.DQVEKL....
ZmbHLH088  KRGCATH........PRSIAE.R......ERR...........TR......ISEK..LRKLQA.L...............VP..NM..D.............K.QT.S...TA.DMLDLAVDHIRGLQ.NELQAL....
ZmbHLH089  GGAP..S........KNLMAE.R......RRR...........KR......LNDR..LSMLRS.I...............VP..KI..S.............K..M.D...RT.SILGDTIDYVKELT.ERIKVL....
ZmbHLH090  EGMP..S........KNLMAE.R......RRR...........KR......LNDR..LSMLRS.V...............VP..KI..S.............K..M.D...RT.SILGDTIDYMKELL.ERIKLL....
ZmbHLH091  VGMS..S........KNLMAE.R......RRR...........KR......LNGR..LSMLQS.V...............VP..KI..N.............K..M.D...WT.SILGDTIDYMKELL.ERIKLL....
ZmbHLH092  KRLP..A........KNLMAE.R......RRR...........KK......LNDR..LYMLRS.V...............VP..KI..S.............K..M.D...RA.SILGDAIEYLKELL.RKIEEL....
ZmbHLH093  KGMP..A........KNLMAE.R......RRR...........KK......LNDR..LYMLRS.V...............VP..KI..S.............K..M.D...RA.SILGDAIEYLKELL.QRISDL....
ZmbHLH094  KGLP..A........KNLMAE.R......RRR...........KK......LNDR..LYMLRS.V...............VP..KI..S.............K..M.D...RA.SILGDAIEYLKELL.QKINDL....
ZmbHLH095  KRQQ..C........KNLEAE.R......KRR...........KK......LNER..LYKLRS.V...............VP..NI..S.............K..M.D...RA.AILGDAIDYIVGLQ.NQVKAL....
ZmbHLH096  TTT..TT........RNMDME.R......TRR...........RK......LNER..LYALRS.V...............VP..NI..T.............K..M.D...KA.SIVRDAIAHIEHLQ.EQERRL....
ZmbHLH097  AAA..AN........KNILME.R......DRR...........RK......LNEK..LYALRS.V...............VP..NI..T.............K..M.D...KA.SIIKDAIEYIEQLQ.AEERRA....
ZmbHLH098  AAC..GN........KNILME.R......DRR...........RK......LNDK..LYALRS.V...............VP..NI..T.............K..M.D...KA.SIIKDAIEYIQQLQ.VEERRV....
ZmbHLH099  RRDR..S........KTIVSE.R......KRR...........VR......MKEK..LYELRA.L...............VP..NI..T.............K..M.D...KA.SIIADAVVYVKNLQ.AHARKL....
ZmbHLH100  RRDR..S........KTIVSE.R......KRR...........VR......MKEK..LYELRA.L...............VP..NI..T.............K..M.D...KA.SIIADAVVYVKNLQ.AHARKL....
ZmbHLH101  RRDR..S........KTIVSE.R......KRR...........VR......MKEK..LYELRS.L...............VP..NI..T.............KVQM.D...KA.SIIADAVVYVKNLQ.AHARKL....
ZmbHLH102  PAAEFKS........KNLEAE.R......KRR...........GK......LNRN..ILALRA.V...............VP..NI..T.............K..M.S...KE.STLSDAIDLIKRLQ.NQVLEL....
ZmbHLH103  REEP..L........NHVEAE.R......QRR...........EK......LNQR..FYALRA.V...............VP..NV..S.............K..M.D...KA.SLLGDAISYINELR.GKLTSL....
ZmbHLH104  REEP..L........NHVEAE.R......QRR...........EK......LNQR..FYALRA.V...............VP..NV..S.............K..M.D...KA.SLLGDAISYINELR.GKLTSL....
ZmbHLH105  REEP..L........NHVEAE.R......QRR...........EK......LNQR..FYALRA.V...............VP..NI..S.............K..M.D...KA.SLLGDAITYITDLQ.KKLKEM....
ZmbHLH106  REEP..L........NHVEAE.R......QRR...........EK......LNQR..FYALRA.V...............VP..NI..S.............K..M.D...KA.SLLGDAITYITDLQ.KKLKEM....
ZmbHLH107  REEP..L........NHVEAE.H......QRR...........EK......LNQR..FYALRA.V...............VP..NI..S.............K..M.D...KA.SLLGDAITYIPDER.VALPPL....
ZmbHLH108  REEP..L........NHVEAE.R......QRR...........EK......LNQR..FYALRA.V...............VP..KI..S.............K..M.D...KA.SLLSDAIAYIQELE.DRLRGG....
ZmbHLH109  ..PT..L........SHVEAE.R......QRR...........DK......LNRR..FCELRA.A...............VP..TV..S.............R..M.D...KA.SLLADAATYIGELR.DRVEQL....
ZmbHLH110  ..PT..V........SHVEAE.R......QRR...........EK......LNRR..FCDLRA.A...............VP..TV..S.............R..M.D...KA.SLLADAAAYIAELR.GRIARL....
ZmbHLH111  ..PA..V........SHVESE.R......QRR...........EK......LNRR..FCDLRA.A...............VP..TV..S.............R..M.D...KA.SLLADAASYIAELR.GRVARL....
ZmbHLH112  ..PG..I........SHVEAE.R......QRR...........EK......LNRR..FCDLRA.A...............VP..TV..S.............R..M.D...KA.SLLADAAAYIAELR.GRVEQL....
ZmbHLH113  RMHWNTQ........EHVIAE.R......KRR...........EK......MQQQ..FVTLAT.I...............VP..DL..T.............KMDQ.D...KI.SLLGSTIEYVKHLE.EKVKAL....
ZmbHLH114  RASSGVH........EHIVAE.R......MRR...........QK......MNHQ..FAALAS.M...............IP..DI..T.............K..T.D...KV.SLLGSTIEYVQHLR.GRLKAL....
ZmbHLH115  SSPGPVQ........DHIIAE.R......RRR...........EK......INQR..FIELST.V...............IP..GL..K.............K..M.D...KA.TILGDAVKYVRELQ.EKVKGL....
ZmbHLH116  SSPGPVQ........DHIIAE.R......RRR...........EK......INQR..FIELST.V...............IP..GL..K.............K..M.D...KA.TILGDAVKYVRELQ.EKVKGL....
ZmbHLH117  SSAAYAQ........DHIIAE.R......KRR...........EK......INQR..FIELST.V...............IP..GL..K.............K..M.D...KA.TILSDATKYVKELH.GKLKDL....
ZmbHLH118  MAAAYAK........DHIIAE.R......KRR...........EK......INQR..FIELST.V...............IP..GL..K.............K..M.D...KA.TILLDATRYLKELQ.EKLKDL....
ZmbHLH119  VGSIYAQ........DHIIAE.R......KRR...........EK......INQR..FIELST.V...............IP..GL..K.............K..M.D...KA.TILSDATRYVRDLQ.EKIKAH....
ZmbHLH120  SAPPCSQ........DHIVAE.R......KRR...........EK......INQR..FIELSA.V...............IP..CL..K.............K..M.D...KA.TILSDATRYVKELQ.EKLKAL....
ZmbHLH121  GAASCTP........EHIVAE.R......KRR...........EK......INNR..LIELST.V...............IP..GL..K.............K..M.D...KA.TILSDAAKYVKELQ.QRLKAL....
ZmbHLH122  RPASQNQ........DHILAE.R......KRR...........EK......LSQR..FIALSK.I...............VP..GL..K.............K..M.D...KA.SVLGDAIKYVKQLQ.DQVKGL....
ZmbHLH123  RPASQNQ........DHILAE.R......KRR...........EK......LSQR..FIALSK.I...............VP..GL..K.............K..M.D...KA.SVLGDAIKYVKQLQ.DQVKGL....
ZmbHLH124  AQENGAK........NHVMLE.R......KRR...........EK......LNEM..FLVLKS.L...............VP..SI..H.............K..V.D...KA.SILAETIAYLKELQ.RRVQEL....
ZmbHLH125  MSGTGTK........NHVMSE.R......KRR...........EK......LNEM..FLVLKS.L...............LP..SI..H.............R..V.N...KA.SILAETIAYLKELQ.RRVQEL....
ZmbHLH126  KALAASR........SHSEAE.R......RRR...........ER......INAH..LARLRS.L...............LP..NT..T.............K..V.........LILIIIDDITHAR.IE........
ZmbHLH127  LRKERGR........SHSEAE.R......KRR...........QR......INAH..LATLRT.L...............VP..SA..S.............R..M.D...KA.ALLGEVVRYVRELR.EKASDA....
ZmbHLH128  RS.GTGR........SHSEAE.R......KRR...........QR......INAH..LATLRT.L...............LP..AA..S.............R..M.D...KA.ALLGEVVRHVRELR.GEADAA....
ZmbHLH129  RSGGTSR........SHSEAE.R......KRR...........QR......INAH..LATLRT.L...............LP..AA..S.............R..M.D...KA.ALLGEVVRHVRELR.GEADAA....
ZmbHLH130  KALAASR........SHSEAE.R......SRR...........QR......INGH..LAKLRS.L...............LP..NT..T.............K..T.D...KA.SLLAEVIEHVKELK.RQTSAA....
ZmbHLH131  KALAASR........SHSEAE.R......RRR...........QR......INGH..LARLRS.L...............LP..NT..T.............K..T.D...KA.SLLAEVLDHVKELK.RQTSAM....
ZmbHLH132  KALAASR........SHSEAE.R......RRR...........QR......INSH..LARLRS.L...............LP..NT..S.............K..T.D...KA.SLLAEVIEHVKELK.RQTSAV....
ZmbHLH133  KALAASK........SHSEAE.R......RRR...........ER......INAH..LARLRS.L...............LP..NT..T.............K..T.D...KA.SLLAEVIQHVKELK.RQTSEI....
ZmbHLH134  KAAMALK........SHSEAE.R......RRR...........ER......INAH..LATLRT.M...............VP..CS..D.............K..M.D...KA.AVLAEVITHVKKLK.STAAHI....
ZmbHLH135  KAAMALK........SHSEAE.R......RRR...........ER......INAH..LATLRT.M...............VP..CS..D.............K..M.D...KA.AVLAEVITHVKKLK.STAAHI....
ZmbHLH136  RALAASR........NHREAE.K......RRR...........ER......IKSH..LDRLRN.V...............LA..CD..P.............K..I.D...KA.SLLAKAVERVRDLK.QRAAGV....
ZmbHLH137  EAR.ALK........VHSEAE.R......RRR...........ER......INAH..LATLRR.M...............VP..DT..R.............Q..M.D...KA.TLLARVVEQVKLLK.RKASEA....
ZmbHLH138  EARQALK........IHSAAE.K......RRR...........ER......INAH..LATLRR.M...............IP..DA..S.............Q..M.D...KA.TLLARVVCQLKDLK.KKSAET....
ZmbHLH139  VEDVDHD........LHIFTE.R......ERR...........KK......MKNM..FSTLHA.L...............LP..QL..PD............K..A.D...KA.TIVGEAVTYIKTLE.GTVQKL....
ZmbHLH140  ..AASKKLK......PHIVAE.R......KRR...........DR......MRDY..FGELLA.L...............MP..QI..PA............K..S.D...KG.TIVGHAVDYIQ..............
ZmbHLH141  GPNTPRS........KHSATE.Q......RRR...........SK......INDR..FQILRE.L...............LP..HN..DQ............K..R.D...KA.TFLLEVIEYIRFLQ.EKAQKY....
ZmbHLH142  RPNTPRS........KHSATE.Q......RRR...........SK......INDR..FQILRE.L...............LP..HS..DQ............K..R.D...KA.TFLLEVIEYIRFLQ.EKVQKY....
ZmbHLH143  GPSTPRS........KHSATE.Q......RRR...........TK......INDR..LEILRE.L...............LP..HG..DQ............K..R.D...KA.SFLLEVIEYIRFLQ.EKVQKY....
ZmbHLH144  .PPSVNQL.......QHMFSE.R......KRR...........EK......LNDS..FHALKA.V...............LPP.G...A.............K..K.D...KT.SILIRAREYVRSLE.ARVAEL....
ZmbHLH145  .PPSVNQL.......QHMFSE.R......KRR...........EK......LNDS..FHALKA.A...............LPP.G...A.............K..K.D...KT.SILIRARDYVRSLE.AKVAEL....
```

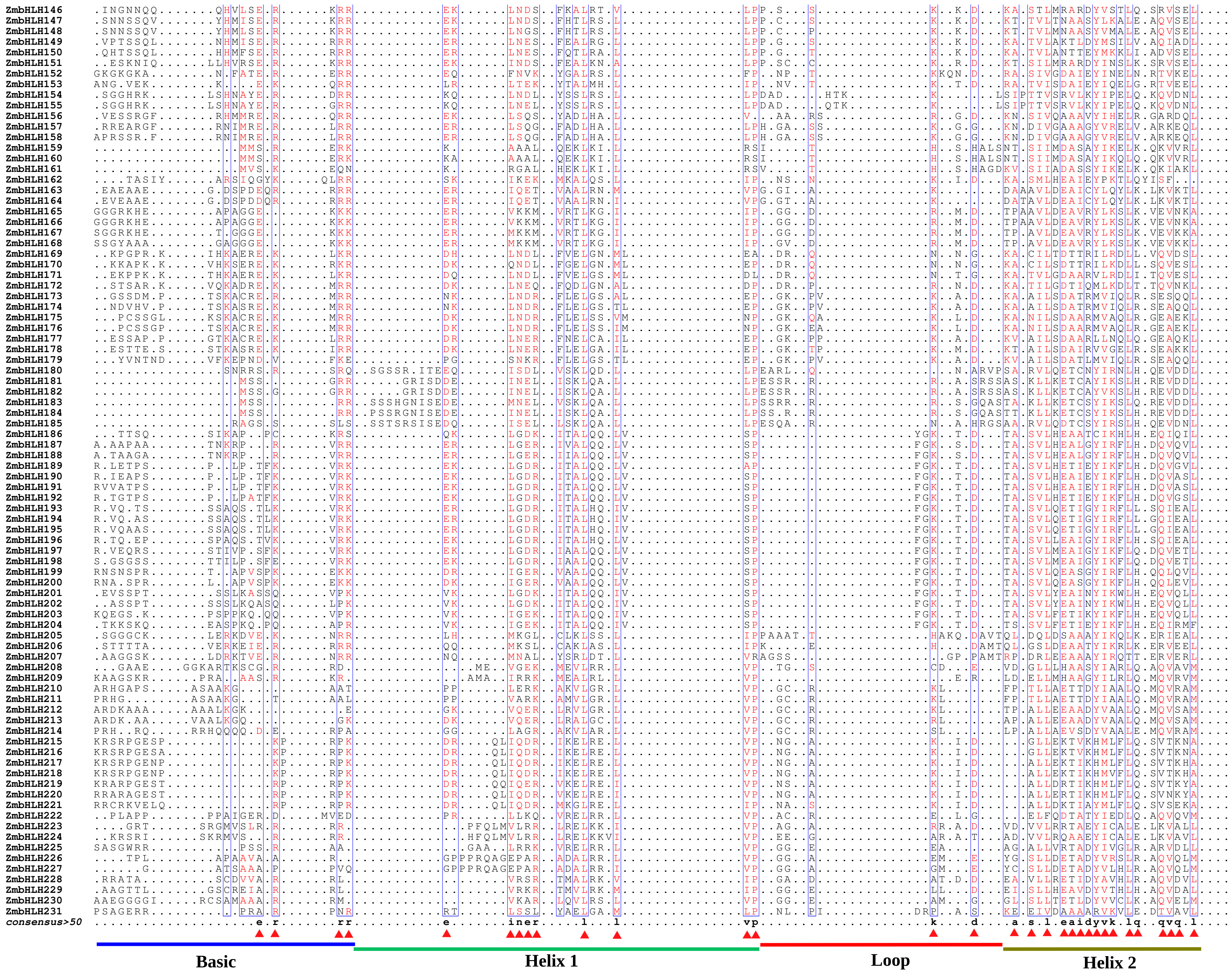

Figure S1. Amino acid sequence alignment of 1154 Arabidopsis, rice, wheat and maize bHLH domains. The red triangles at the bottom indicate the 31 conserved residues. The loop region has been shortened to better visualize the alignment results.
