# Supplementary figures and images for: Comparative functional genomics analysis of bHLH gene family in rice, maize and wheat
Source: BMC Plant Biol. 2018 Nov 29;18:309. doi: 10.1186/s12870-018-1529-5 (PMC6267037; doi:10.1186/s12870-018-1529-5)

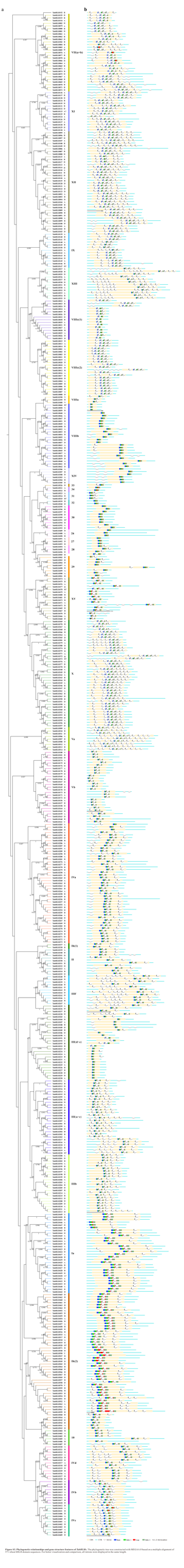

Supplement: Supplementary file 7 — Figure S3. Phylogenetic relationships and gene structure features of TabHLHs. The phylogenetic tree was constructed with MEGA 6.0 based on a multiple alignment of 571 wheat bHLH domain sequences. For better visualization and comparison, all introns were displayed in the same length. (PDF 5746 kb) [file 12870_2018_1529_MOESM7_ESM.pdf]

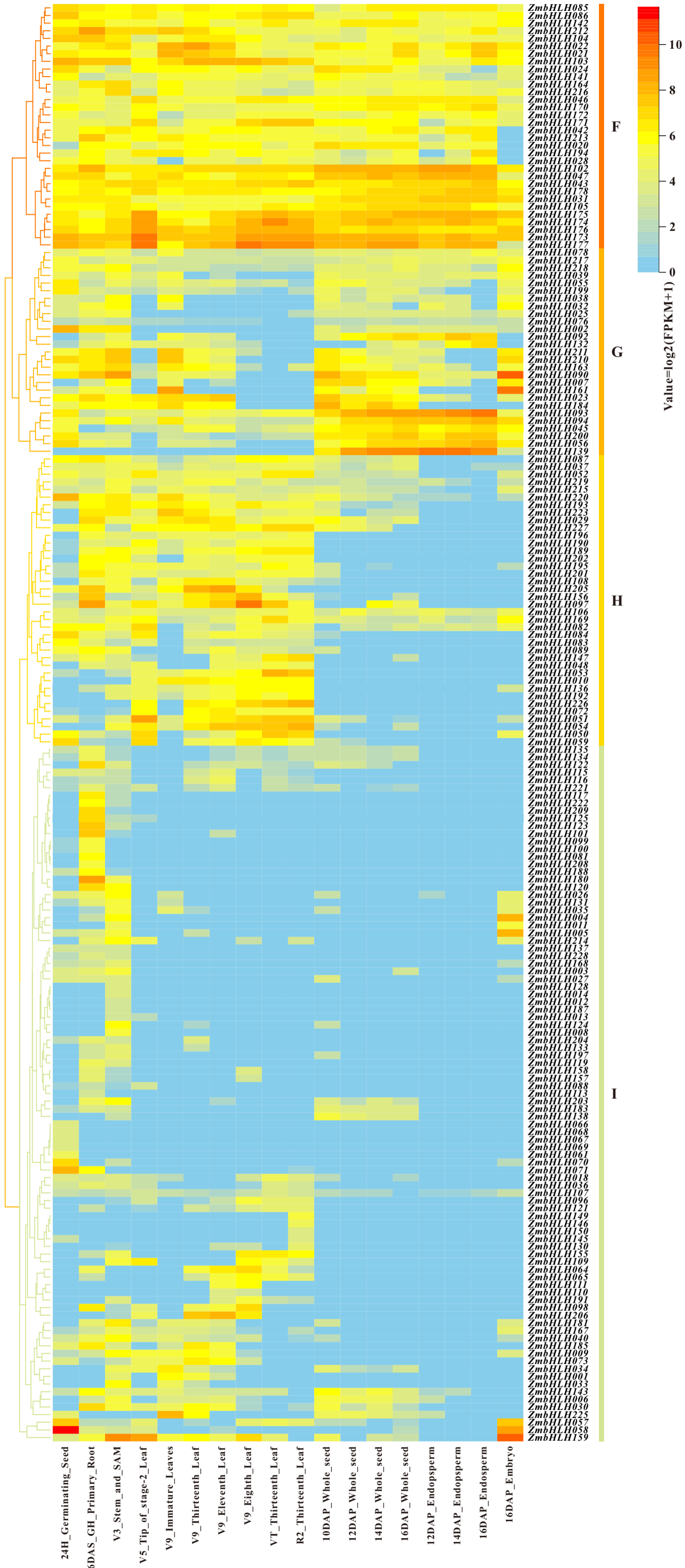

Figure S7. Heatmap showing the transcriptional abundance of ZmbHLHs in 18 tissues.

Supplement: Supplementary file 17 — Figure S7. Heatmap showing the transcriptional abundance of ZmbHLHs in 18 tissues. (PDF 448 kb) [file 12870_2018_1529_MOESM17_ESM.pdf]

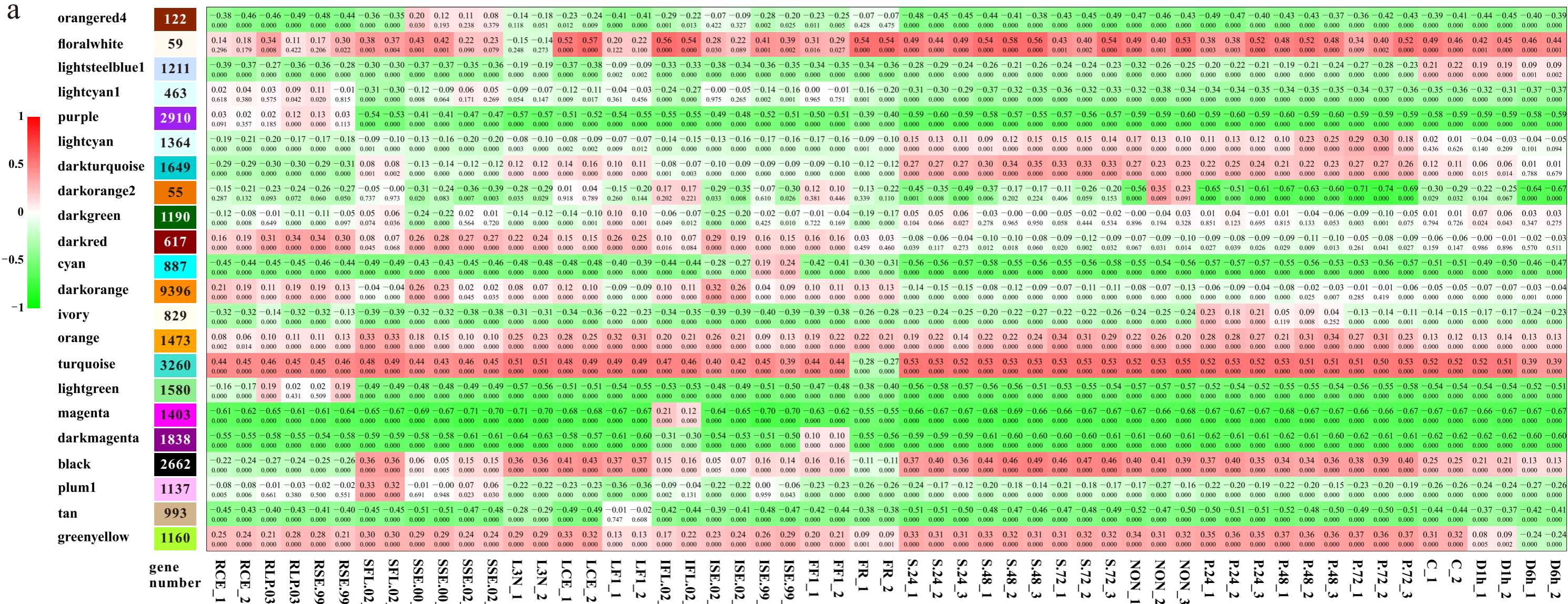

Supplement: Supplementary file 27 — Figure S10. Module-tissue association. Each row corresponds to a module and each column corresponds to a tissue. The top and bottom number in each cell indicate the correlation coefficient between the module and tissue and p-value of the test, respectively. (PDF 3815 kb) [file 12870_2018_1529_MOESM27_ESM.pdf]
