# Supplementary material for: Comparative functional genomics analysis of bHLH gene family in rice, maize and wheat
Source: BMC Plant Biol. 2018 Nov 29;18:309. doi: 10.1186/s12870-018-1529-5 (PMC6267037; doi:10.1186/s12870-018-1529-5)
Supplement: Supplementary file 11 — Figure S6. Arrangement of secondary structure elements in AtMYC3 (5–242). Secondary structure elements overlaid on the sequence alignment of bHLH N-terminal proteins belonging to three subfamilies III(d + e), XIII and IIIf. (PDF 517 kb) [file 12870_2018_1529_MOESM11_ESM.pdf]

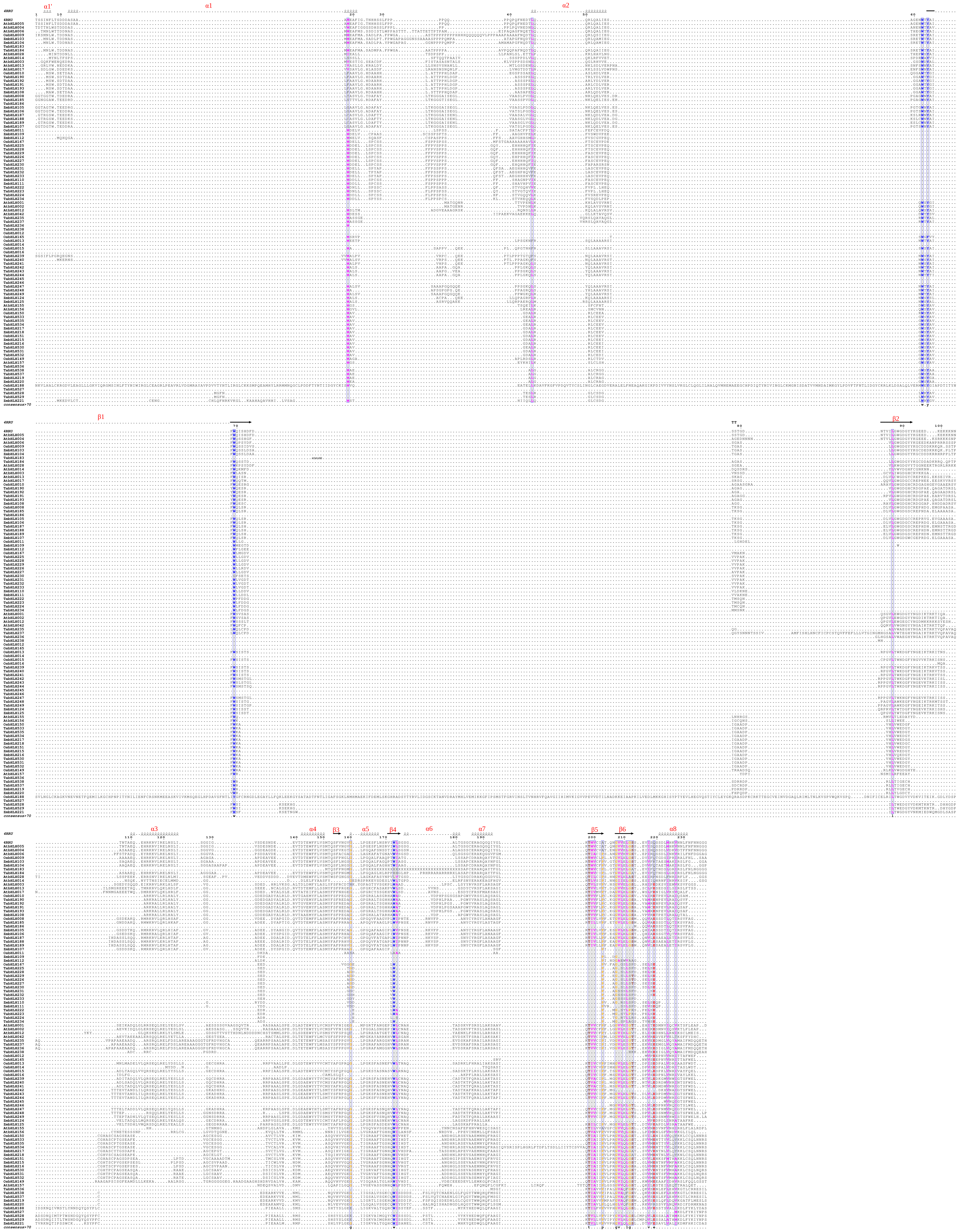

Figure S6. Arrangement of secondary structure elements in AtMYC3 (5–242). Secondary structure elements overlaid on the sequence alignment of bHLH N-terminal proteins belonging to three subfamilies III(d+e), XIII and IIIf.
