# Supplementary material for: Comparative functional genomics analysis of bHLH gene family in rice, maize and wheat
Source: BMC Plant Biol. 2018 Nov 29;18:309. doi: 10.1186/s12870-018-1529-5 (PMC6267037; doi:10.1186/s12870-018-1529-5)
Supplement: Supplementary file 26 — Figure S9. Analysis of network topology for various soft-thresholding powers. a. The scale-free fit index (y-axis) as a function of the soft-thresholding power (x-axis). b. The mean connectivity (degree, y-axis) as a function of the soft-thresholding power (x-axis). (PDF 469 kb) [file 12870_2018_1529_MOESM26_ESM.pdf]

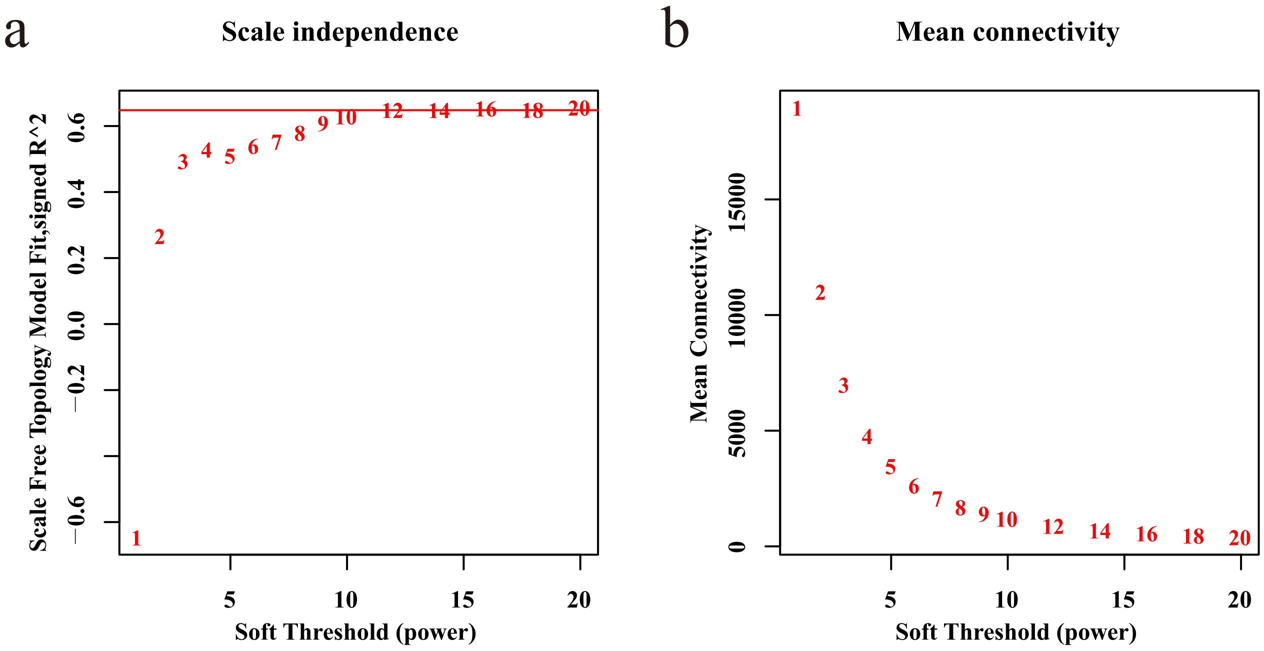

Figure S9. Analysis of network topology for various soft-thresholding powers. a. The scale-free fit index (y-axis) as a function of the soft-thresholding power (x-axis). b. The mean connectivity (degree, y-axis) as a function of the soft-thresholding power (x-axis).
