# Supplementary material for: Comparative functional genomics analysis of bHLH gene family in rice, maize and wheat
Source: BMC Plant Biol. 2018 Nov 29;18:309. doi: 10.1186/s12870-018-1529-5 (PMC6267037; doi:10.1186/s12870-018-1529-5)
Supplement: Supplementary file 31 — Figure S11. Plyhogenetic analysis of 140 TaDEFLs, 7 AtPDF1s and 6 AtPDF2s. The phylogenetic tree was constructed using MEGA by the NJ method with 1000 bootstrap replications, and the bootstrap values greater than 700 were displayed on the branches. (PDF 411 kb) [file 12870_2018_1529_MOESM31_ESM.pdf]

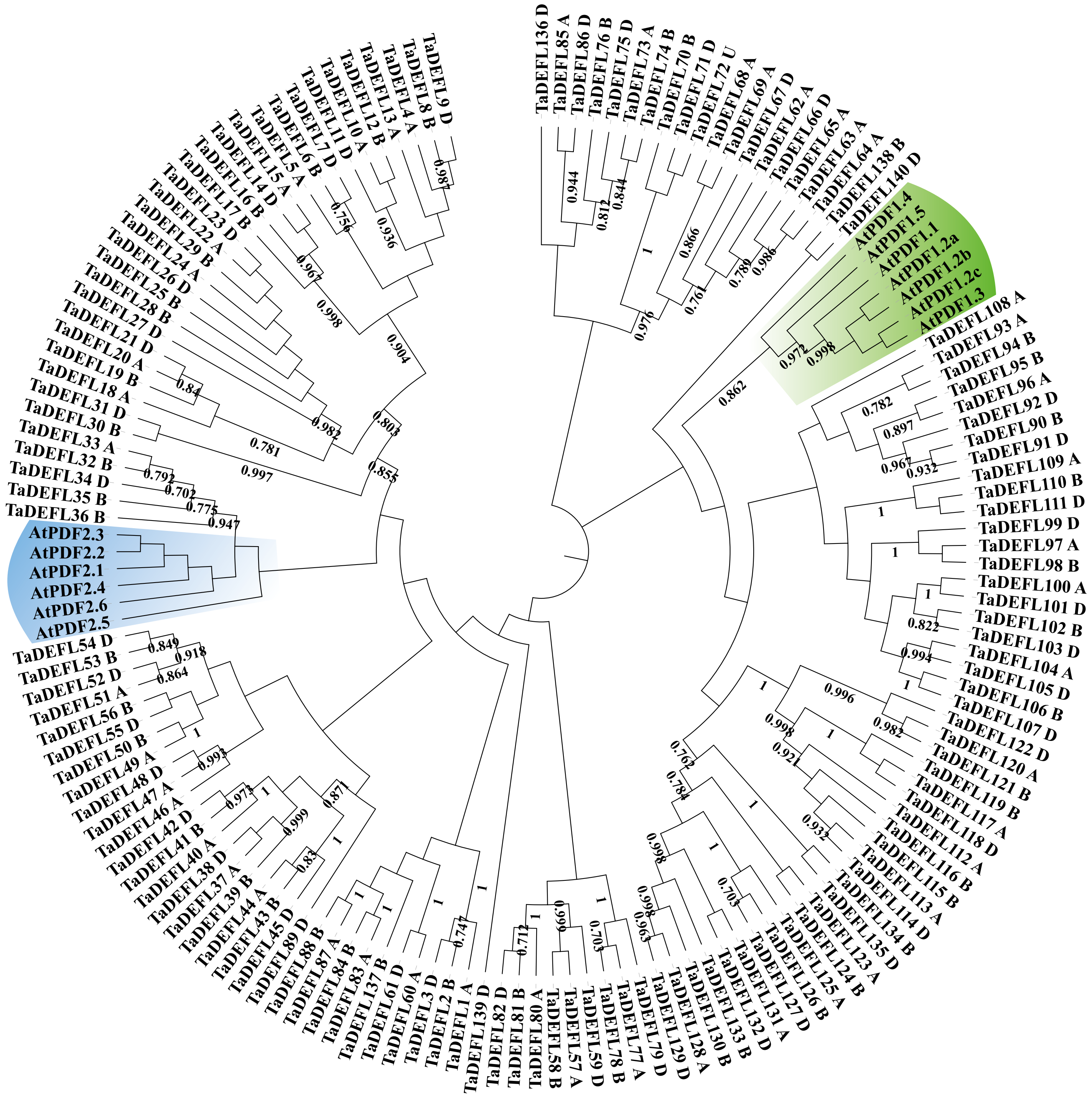

Figure S11. Plyhogenetic analysis of 140 TaDEFLs, 7 AtPDF1s and 6 AtPDF2s. The phylogenetic tree was constructed using MEGA by the NJ method with 1000 bootstrap replications, and the bootstrap values greater than 700 were displayed on the branches.
