# Supplementary material for: Comparative functional genomics analysis of bHLH gene family in rice, maize and wheat
Source: BMC Plant Biol. 2018 Nov 29;18:309. doi: 10.1186/s12870-018-1529-5 (PMC6267037; doi:10.1186/s12870-018-1529-5)
Supplement: Supplementary file 32 — Figure S12. Phylogenetic relationship and motif compositions of TaJAZs. a. The phylogenetic tree of TaJAZs constructed using MEGA by the NJ method with 100 bootstrap replicates. b. Domain distribution of TaJAZs was investigated using the MEME web server. c. The consensus sequence of TIFY and Jas motif from TaJAZs. (PDF 414 kb) [file 12870_2018_1529_MOESM32_ESM.pdf]

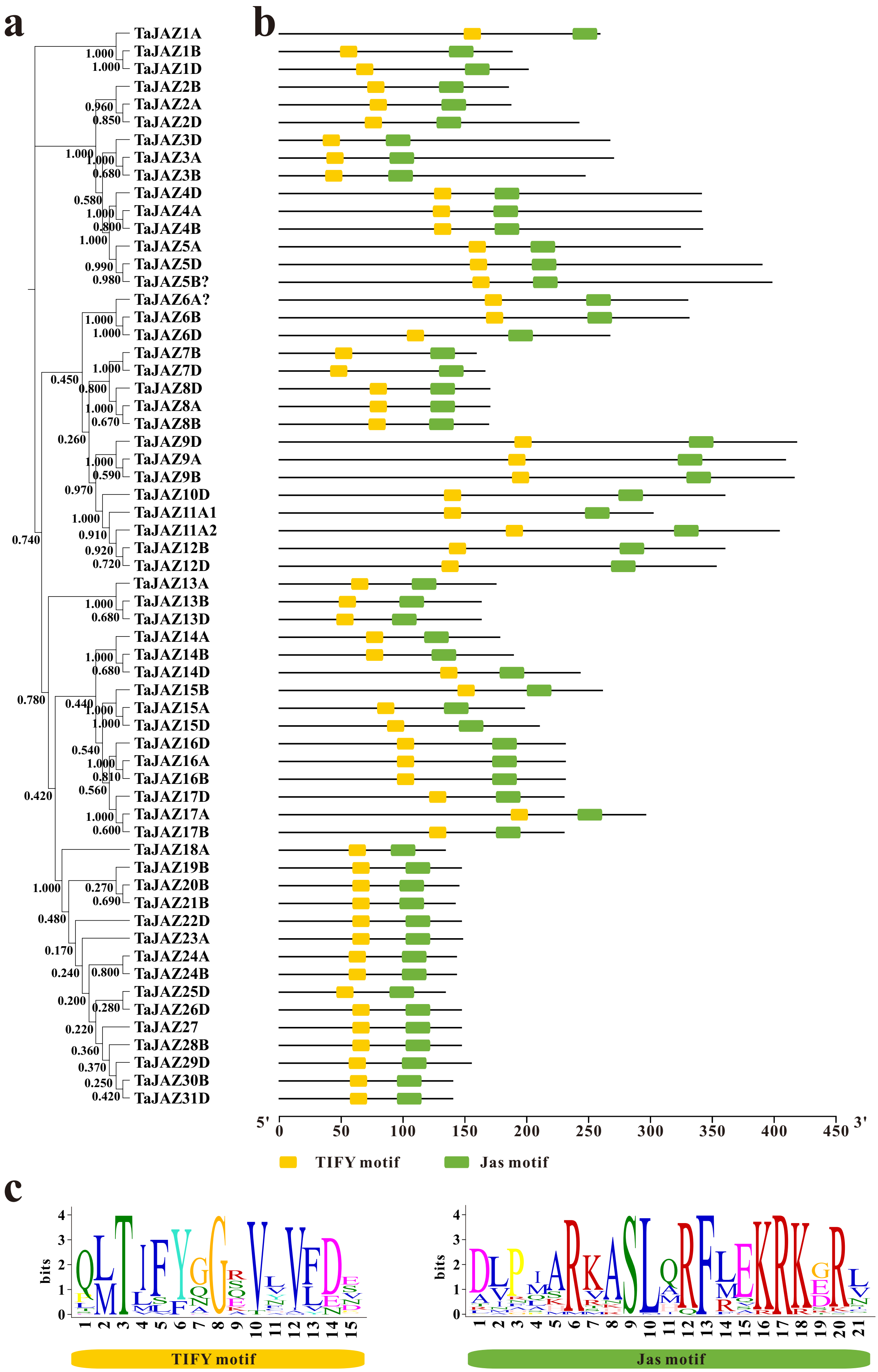

Figure S12. Phylogenetic relationship and motif compositions of TaJAZs. a. The phylogenetic tree of TaJAZs constructed using MEGA by the NJ method with 100 bootstrap replicates. b. Domain distribution of TaJAZs was investigated using the MEME web server. c. The consensus sequence of TIFY and Jas motif from TaJAZs.
