# Supplementary material for: Comparative functional genomics analysis of bHLH gene family in rice, maize and wheat
Source: BMC Plant Biol. 2018 Nov 29;18:309. doi: 10.1186/s12870-018-1529-5 (PMC6267037; doi:10.1186/s12870-018-1529-5)
Supplement: Supplementary file 8 — Figure S4. Phylogenetic relationships and gene structural features of ZmbHLHs. The phylogenetic tree was constructed with MEGA 6.0 based on a multiple alignment of 231 amino acid sequences of maize bHLH domain. For better visualization and comparison, all introns were displayed in the same length. (PDF 1617 kb) [file 12870_2018_1529_MOESM8_ESM.pdf]

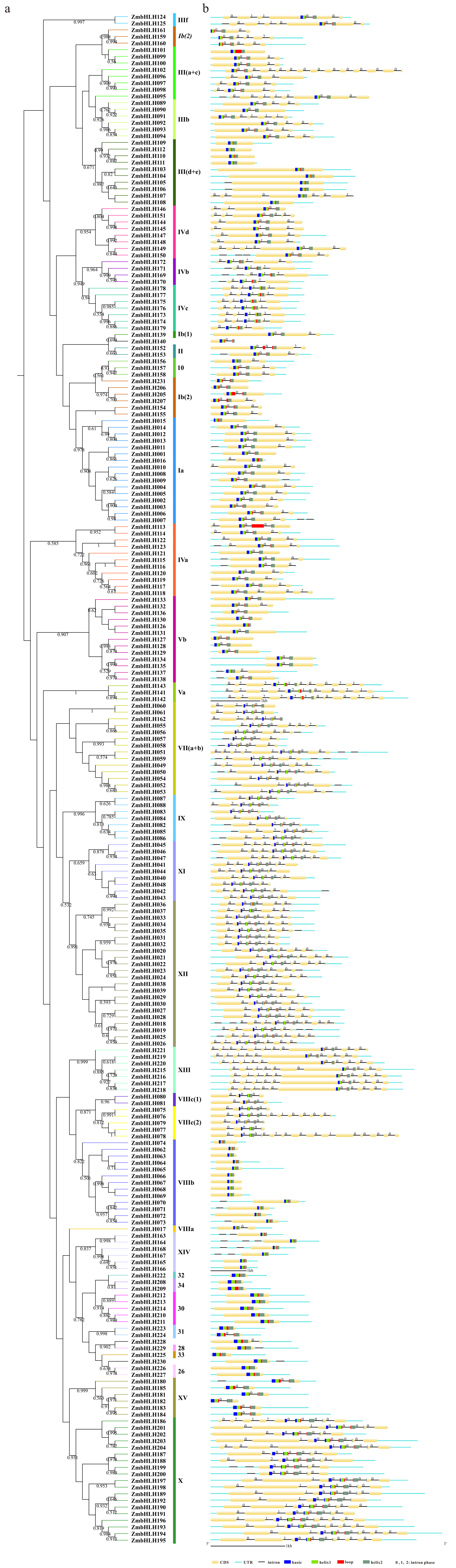

Figure S4. Phylogenetic relationships and gene structural features of *ZmbHLHs*. The phylogenetic tree was constructed with MEGA 6.0 based on a multiple alignment of 231 amino acid sequences of maize bHLH domain. For better visualization and comparison, all introns were displayed in the same length.
