# Supplementary material for: Comparative functional genomics analysis of bHLH gene family in rice, maize and wheat
Source: BMC Plant Biol. 2018 Nov 29;18:309. doi: 10.1186/s12870-018-1529-5 (PMC6267037; doi:10.1186/s12870-018-1529-5)
Supplement: Supplementary file 10 — Figure S5. Architecture of protein conserved motifs. Motifs are graphically represented as colored boxes drawn to scale for bHLH proteins. (PDF 19037 kb) [file 12870_2018_1529_MOESM10_ESM.pdf]

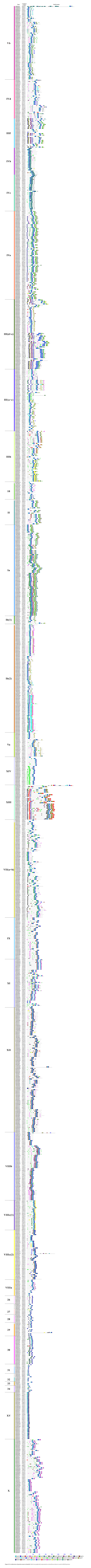

Figure S5. Architecture of protein conserved motifs. Motifs are graphically represented as colored boxes drawn to scale for bHLH proteins.
