# Supplementary material for: Comparative functional genomics analysis of bHLH gene family in rice, maize and wheat
Source: BMC Plant Biol. 2018 Nov 29;18:309. doi: 10.1186/s12870-018-1529-5 (PMC6267037; doi:10.1186/s12870-018-1529-5)
Supplement: Supplementary file 18 — Figure S8. Heatmap showing log2-transformed (FPKM+1) values for OsbHLHs in 7 tissues. (PDF 850 kb) [file 12870_2018_1529_MOESM18_ESM.pdf]

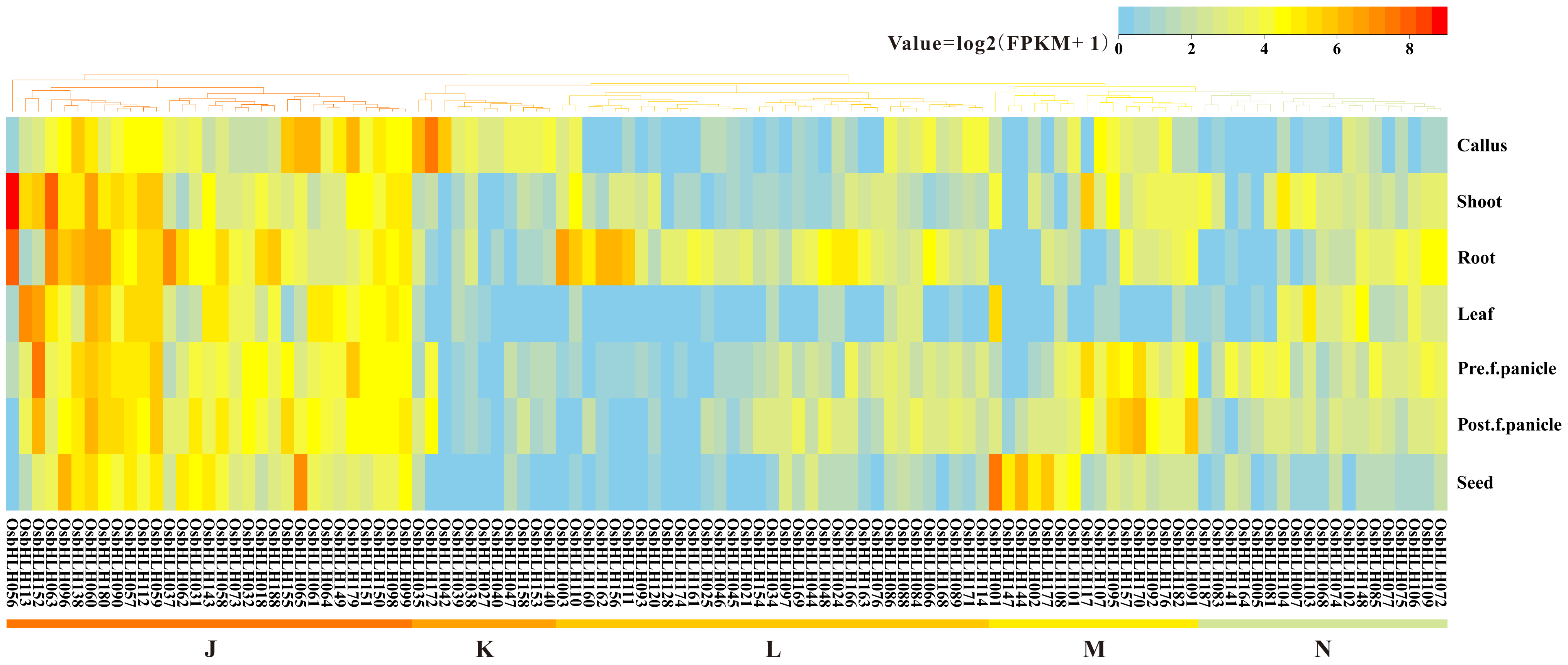

Figure S8. Heatmap showing log2-transformed (FPKM+1) values for OsbHLHs in 7 tissues.
